# Cognition–Eye–Brain Connection in Alzheimer's Disease Spectrum Revealed by Multimodal Imaging

**DOI:** 10.1002/jmri.70003

**Published:** 2025-06-29

**Authors:** Yan Shi, Tingting Shen, Su Yan, Jianwen Liang, Tianyunxi Wei, Yijin Huang, Rong Gao, Ning Zheng, Renpuchi Ci, Min Zhang, Xiaoying Tang, Yuanyuan Qin, Wenzhen Zhu

**Affiliations:** ^1^ Department of Radiology Tongji Hospital, Tongji Medical College, Huazhong University of Science and Technology Wuhan China; ^2^ Department of Electronic and Electrical Engineering Southern University of Science and Technology Shenzhen China; ^3^ Department of Neurology Tongji Hospital, Tongji Medical College, Huazhong University of Science and Technology Wuhan China; ^4^ Clinical & Technical Support Philips Healthcare Wuhan China; ^5^ Department of Neurology Shanxi Bethune Hospital, Shanxi Academy of Medical Sciences, Tongji Shanxi Hospital, Third Hospital of Shanxi Medical University Taiyuan China

**Keywords:** Alzheimer's disease spectrum, brain, cognition, retina

## Abstract

**Background:**

The connection between cognition, eye, and brain remains inconclusive in Alzheimer's disease (AD) spectrum disorders.

**Purpose:**

To explore the relationship between cognitive function, retinal biometrics, and brain alterations in the AD spectrum.

**Study Type:**

Prospective.

**Subjects:**

Healthy control (HC) (*n* = 16), subjective cognitive decline (SCD) (*n* = 35), mild cognitive impairment (MCI) (*n* = 18), and AD group (*n* = 7).

**Field Strength/Sequence:**

3‐T, 3D T1‐weighted Brain Volume (BRAVO) and resting‐state functional MRI (fMRI).

**Assessment:**

In all subgroups, cortical thickness was measured from BRAVO and segmented using the Desikan–Killiany–Tourville (DKT) atlas. The fractional amplitude of low‐frequency fluctuations (FALFF) and regional homogeneity (ReHo) were measured in fMRI using voxel‐based analysis. The eye was imaged by optical coherence tomography angiography (OCTA), with the deep learning model FARGO segmenting the foveal avascular zone (FAZ) and retinal vessels. FAZ area and perimeter, retinal blood vessels curvature (RBVC), thicknesses of the retinal nerve fiber layer (RNFL) and ganglion cell layer‐inner plexiform layer (GCL‐IPL) were calculated. Cognition–eye–brain associations were compared across the HC group and each AD spectrum stage using multivariable linear regression.

**Statistical Tests:**

Multivariable linear regression analysis. Statistical significance was set at *p* < 0.05 with FWE correction for fMRI and *p* < 1/62 (Bonferroni‐corrected) for structural analyses.

**Results:**

Reductions of FALFF in temporal regions, especially the left superior temporal gyrus (STG) in MCI patients, were linked to decreased RNFL thickness and increased FAZ area significantly. In AD patients, reduced ReHo values in occipital regions, especially the right middle occipital gyrus (MOG), were significantly associated with an enlarged FAZ area. The SCD group showed widespread cortical thickening significantly associated with all aforementioned retinal biometrics, with notable thickening in the right fusiform gyrus (FG) and right parahippocampal gyrus (PHG) correlating with reduced GCL‐IPL thickness.

**Data Conclusion:**

Brain function and structure may be associated with cognition and retinal biometrics across the AD spectrum. Specifically, cognition–eye–brain connections may be present in SCD.

**Evidence Level:**

2.

**Technical Efficacy:**

3.

**Summary**

- Plain language summary
  ○ This study aimed to investigate the connection between cognition, eye, and brain across Alzheimer's disease (AD) spectrum disorder.
  ○ Researchers analyzed brain MRI, retinal imaging, and cognitive scores from 76 participants, including healthy controls and individuals with subjective cognitive decline (SCD), mild cognitive impairment (MCI), and AD.
  ○ Reduced brain activity in MCI and AD correlated with retinal thinning and an enlarged foveal avascular zone.
  ○ In SCD, widespread cortical thickening linked to retinal changes. Multimodal imaging revealed significant correlations among cognitive function, retinal biometrics, and brain alterations, offering a practical framework for early detection of the AD spectrum.

## 1 Introduction

Alzheimer's disease (AD) is a neurodegenerative disorder characterized by progressive cognitive decline, and it is the most common type of dementia among the elderly [1]. Specifically, AD spectrum (ADS) disorders encompass stages from preclinical to severe dementia, classified into six clinical stages by the National Institute on Aging and the National Institute on Aging and the Alzheimer's Association (NIA‐AA) according to the 2018 criteria [2]. Subjective cognitive decline (SCD) is the earliest clinical manifestation, while mild cognitive impairment (MCI) serves as a transitional stage between normal aging and dementia [3]. Typically, AD and MCI patients show specific changes in brain structure and function, particularly in the posterior cingulate/precuneus and para‐hippocampal gyrus, which are considered early biomarkers for cognitive impairment [4]. Liu et al. and Zhang et al. discovered that the regional homogeneity (ReHo) values, computed via Kendall's concordance coefficient (KCC) among 26 adjacent voxels, were reduced in the default mode network (DMN) regions of MCI patients [5, 6]. Some studies have reported subtle changes in SCD patients [7, 8], but the conclusions remain controversial. For instance, Risacher et al. [7] observed no cortical structure changes in SCD, whereas Imabayashi et al. [8] found enlargement of the medial temporal lobe.

The eyes share embryonic origins with the central nervous system [9]. The retina, as a part of the central nervous system, shares many structural and functional similarities with the brain [10]. Optical coherence tomography angiography (OCTA) is a non‐invasive retinal imaging technology that can conduct rapid quantitative dynamic scanning analysis of retinal blood flow and measure the thickness of various layers of the retina [11]. Recent studies have shown that OCTA can detect retinal thinning and vascular changes in preclinical AD [11, 12]. In the retinas of AD patients, key pathological biomarkers, namely amyloid‐β (Aβ) plaques and neurofibrillary tangles (NFTs) composed of tau protein, can be detected [13]. In AD animal models, retinal Aβ plaques can be detected earlier than those in the brain and continuously accumulate as the disease progresses [14].

The connection between cognitive, retinal, and brain alterations remains inconclusive. Recent studies showed a correlation between alterations of retinal structure and brain structure in AD and MCI patients [15, 16, 17]. For example, Den Haan et al. found that macular thickness was related to parietal atrophy in AD patients [15]. However, Zhao et al. found that variations in retinal parameters were not associated with gray matter volume or cerebral blood flow (CBF) in patients with AD [16]. Conversely, a study by Gao et al. found a positive correlation between macular ganglion cell complex thickness and CBF in SCD [17].

Furthermore, some studies tried to explore the relationship between the retina and cognitive function directly [18, 19]. Although early changes in retinal thickness have been observed in AD patients with subtle memory disturbances, Cipollini et al. suggested that the correlations between retinal thinning and cognitive performance warrant further investigation [18]. Compared to studies on brain structure, there was less research on the correlation between brain function and ophthalmic indicators, making it even more challenging to draw definitive conclusions [20]. Since brain structure and function may drive cognitive changes, investigating the relationship between cognitive, retinal, and brain structure and function alterations is critical for the understanding of cognition–eye–brain mechanisms in the progression of ADS. However, prior studies have examined either brain–retina or retina–cognition relationships [15, 19], while none have examined the interconnected brain–retina–cognition interactions—a critical gap that motivates our integrated analysis of their interplay during ADS progression.

Against this background, this study aimed to elucidate the cognition–eye–brain connections in ADS disorders by combined neuropsychological assessments of cognitive function, OCTA‐derived retinal biometrics, and brain function and structure measured by MRI.

## 2 Materials and Methods

**FIGURE 1:**
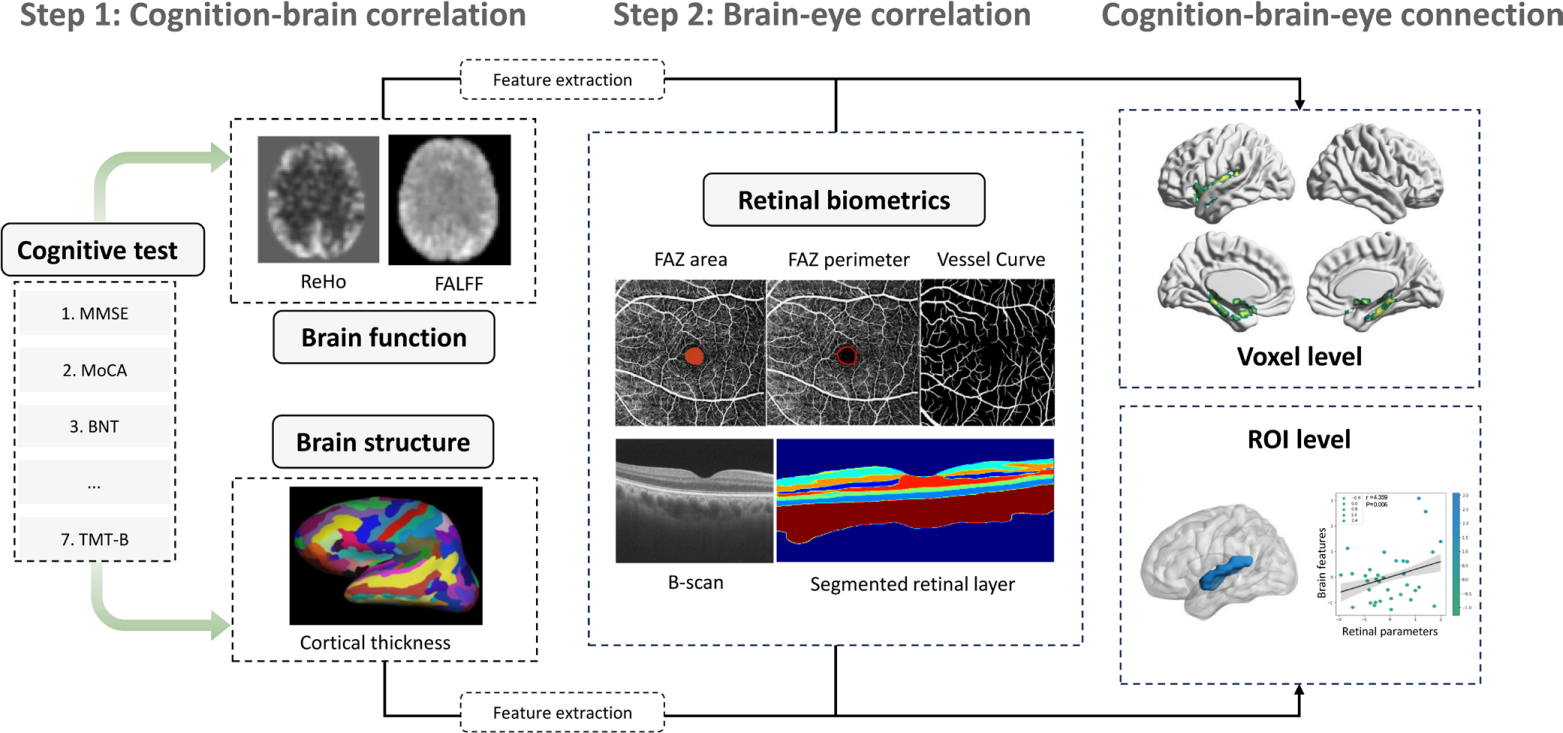
Flow charts of two‐step multivariable linear regression model used for cognition–eye–brain connection analysis. BNT, Boston Naming Test; FALFF, fractional amplitude of low frequency fluctuations; FAZ, foveal avascular zone; MMSE, Mini‐Mental State Examination; MoCA, Montreal Cognitive Assessment; ReHo, regional homogeneity; TMT‐B, Trail Making Test‐part B.

This prospective monocentric study was approved by the local institutional review board (TJ‐IRB202401097). Written informed consent was obtained from all participants. Figure 1 outlines the work design and the overall analytical approach.

### 2.1 Participants and Study Design

This study included four groups: healthy control (HC), SCD, MCI, and AD. The SCD patients met the SCD‐plus diagnostic criteria proposed by Jessen et al. [3]. The MCI patients followed the criteria proposed by Petersen [21]. AD patients met the “probable AD” criteria from the 2011 NIA‐AA guidelines [22]. Exclusion criteria included: [1] eye diseases (such as cataract, glaucoma, and uveitis) or random intraocular pressure (IOP) > 21 mmHg; [2] subjects with best‐corrected visual acuity (BCVA) of < 0.5 (decimal notation); [3] cognitive impairment caused by mental illness or medication; [4] major mental illnesses (such as depression, anxiety disorder). A total of 76 participants were included: 16 in the HC group, 35 in SCD, 18 in MCI, and 7 in the AD group.

General demographic information was collected, including age, sex, years of education, and the presence of hypertension or diabetes, along with a detailed medical history of eye diseases and neurological conditions. All participants were right‐handed. Every participant underwent a comprehensive neuropsychological assessment by a memory specialist, which included the Mini‐Mental State Examination (MMSE), Montreal Cognitive Assessment (MoCA), Boston Naming Test (BNT), Clock Drawing Test (CDT), Backward‐Digit Span Test (BDST), as well as parts A and B of the Trail Making Test (TMT‐A and TMT‐B).

### 2.2 MRI Acquisition

Both structural MRI and resting‐state functional MRI (fMRI) were acquired with a 3‐T MRI scanner (Discovery 750; GE Healthcare, Chicago, IL, USA) in the supine position. Imaging extended from the skull vertex to its base.

Resting‐state fMRI employed a gradient‐echo echo‐planar imaging (EPI) sequence with the following parameters: repetition time (TR)/echo time (TE) = 2000/30 ms, data matrix = 64 × 64, field of view (FOV) = 220 × 220 mm, slice thickness = 3 mm, flip angle = 90°, with 36 slices and 185 volumes. For structural MRI, a 3D T1‐weighted BRAin Volume (BRAVO) sequence was acquired with the following parameters: TR/TE = 7.1/2.7 ms, FOV = 256 × 256 mm, matrix = 256 × 256, number of excitations (NEX) = 1, slice thickness = 1 mm.

### 2.3 Optical Coherence Tomography Angiography

All participants underwent a comprehensive ophthalmic examination, including the evaluation of BCVA and IOP using the NT‐510 device (NIDEK, Gamagori, Aichi, Japan). Ultra‐wide‐field swept‐source OCTA images were acquired using the VG200S device (SMI, Henan, China), with a 1050 nm central wavelength and a scanning speed of 200,000 A‐scans per second. Each scan included 512 vertical B‐scans, with each B‐scan consisting of 512 A‐scans in the horizontal direction, with four repetitions per location. Confocal laser eye‐tracking technology minimized motion artifacts.

All OCTA operations were performed by an ophthalmologist (5 years of specialized clinical experience). The device was adjusted to a suitable height according to the subject's height. One eye was selected, adjusting the scanning head for the clearest fundus image. Scanning modes were selected as Angio 3 × 3, Angio 6 × 6, and ONH Angio 6 × 6. The procedure was repeated for the other eye. Quality assessment ensured excluding low‐quality images (quality index < 6) or those with motion artifacts. Right eye data were primarily analyzed, with left eye data substituted when deemed ineligible. All images met the quality standards, and no images were excluded due to quality issues.

For ophthalmic indicators, the area and perimeter of the foveal avascular zone (FAZ), a capillary‐free region in the macula; retinal blood vessel curve (RBVC); the thickness of the retinal nerve fiber layer (RNFL), the innermost layer of the retina; and the thickness of the ganglion cell layer‐inner plexiform layer (GCL‐IPL), the combined layer of the retina, consisting of the ganglion cell layer and the inner plexiform layer were extracted for analyses.

### 2.4 Data Preprocessing

The preprocessing of brain structural images (3D T1‐weighted BRAVO images) was performed using FreeSurfer software (version 7.4.0, https://surfer.nmr.mgh.harvard.edu) and the resting‐state fMRI data were preprocessed with data processing assistant (DPARSF version 8.0, https://www.fil.ion.ucl.ac.uk/spm/), a MATLAB toolbox (version R2020a, https://github.com/niccolobiasi/CardioMat) based on Statistical Parametric Mapping (SPM12, https://www.fil.ion.ucl.ac.uk/spm/software/spm12/). The first 10 volumes are discarded. The remaining 175 functional sequences underwent slice timing correction and motion realignment. None of the subjects was excluded based on translation > 3 mm or rotation > 3° in any direction.

Next, the skull was stripped from individual structural images using FreeSurfer, and co‐registered to the average volume of resting‐state fMRI. Cortical thickness was segmented using the Desikan–Killiany–Tourville (DKT) atlas [23]. The corrected resting‐state fMRI images were spatially normalized to the Montreal Neurological Institute (MNI) space using the Diffeomorphic Anatomical Registration Through Exponentiated Lie Algebra (DARTEL, DPABI version 8.0_231111 toolkit, https://rfmri.org/dpabi) toolbox and then resampled to 3‐mm isotropic voxels. After removing the linear trend, nuisance signals were regressed out. These included the Friston 24‐head motion parameters, mean global signal, white matter signals, and cerebrospinal fluid signals. The residual time series were used for analyses.

The FAZ is crucial for diagnosing various eye‐related diseases [24]. We used the deep learning model FARGO (https://github.com/lkpengcs/FARGO) [25], based on PyTorch (version 1.8), for accurate segmentation of the FAZ and retinal vessels. For OCTA images centered around the macula, we selected the anterior vascular complex layer, which contains arteriovenous networks. Its high contrast with surrounding tissues enhances the visibility of the FAZ, making it easier to identify. Additionally, the distinct shape and texture features of the blood vessels allow for relatively easy segmentation and recognition in the image, a process that was carried out automatically.

After obtaining the segmented vessels, we used Python image processing and analysis libraries (OpenCV, version 4.6.0.66; scikit‐image, version 0.21) to analyze the data further. Specifically, we applied contour detection to calculate the area of connected regions and remove small objects. Vascular boundaries were determined by 3 × 3 kernel convolution, and vessels curvature was estimated by calculating the gradients and second‐order gradients of the centerline coordinates. The area and perimeter of the FAZ were calculated using Python morphological methods. In addition, we used a TensorFlow‐based OCT retinal layer segmenter (version 2.4.1) to accurately segment OCTA images into different retinal layers, such as RNFL and GCL‐IPL. This enabled the calculation of the average thickness for each layer. To ensure reliability, all segmentation results were also reviewed by the same ophthalmologist.

### 2.5 Metrics Calculation

To measure regional spontaneous brain activity, we used the DPARSF (https://rfmri.org/DPARSF) toolbox to compute two metrics in a voxel‐wise manner: Fractional amplitude of low frequency fluctuations (FALFF, calculated as the ratio of low‐frequency power to total power spectrum, indexing regional spontaneous neural activity) and ReHo, both within the 0.01–0.1 Hz band. In detail, FALFF maps were generated by transforming each voxel's time series into the frequency domain using a fast Fourier transform, calculating the square root of the power spectrum, and averaging data across the specified frequency band. Moreover, ReHo was calculated by the KCC [26] between each voxel and its 26 neighboring voxels, constructing a voxel‐wise ReHo map.

Regarding brain structure, cortical thickness was quantified using Freesurfer segmentation based on DKT atlas, including 62 regions of interest (ROIs; 31 per hemisphere), followed by spatial smoothing with a Gaussian kernel of full width at half maximum (FWHM) of 4 mm to reduce noise and improve the signal‐to‐noise ratio. All metric maps were divided by the mean of all voxels in the brain region to obtain standardized metrics.

### 2.6 Statistical Analysis

Research data including demographics were analyzed using SPSS software (version 12; IBM Corp.; Armonk, NY, USA). The Shapiro–Wilk test assessed normality, and Levene's test was used to check for variance homogeneity. Continuous variables were compared among the four groups using one‐way analysis of variance (ANOVA) or Kruskal–Wallis test for non‐normally distributed data or unequal variances. Categorical variables were compared with *χ* ^2^ tests.

A multivariable linear regression model was used to analyze associations between neuroimaging markers (cortical thickness; FALFF, and ReHo), cognitive function (MMSE, MoCA, BNT, CDT, BDST, TMT‐A, and TMT‐B) and ophthalmic biomarkers (FAZ area/perimeter, RBVC, RNFL thickness, and GCL‐IPL thickness) across the HC group and each ADS stage, controlling for age, sex, education, hypertension, and diabetes. Cortical thickness analyses additionally controlled for estimated total intracranial volume (ETIV).

To examine the correlation between brain functional indicators (FALFF and ReHo) and cognitive function, as well as ophthalmic biometrics, we conducted a two‐stage analysis. In the first stage, we modeled the association between brain functional metrics and cognitive indicators within a gray matter mask created by thresholding (a threshold of 0.2) [27] the mean gray matter probability map of all subjects. In the second stage, the relationship between brain functional metrics and ophthalmic indicators was modeled within the mask of statistically significant voxels from the first stage.

Similarly, to identify brain structural regions related to cognitive indicators and ophthalmic biometrics, we adopted a two‐stage analysis. In the first stage, all cortical regions defined by the DKT atlas were used, and cortical regions significantly associated with cognition were selected. In the second stage, only the cortical regions significant in the first stage were considered. The complete multivariable linear regression model was formulated as follows:

$$\begin{array}{c} Y=\beta_{0}+\beta_{1}\times x_{\mathrm{obj}}+\beta_{2}\times x_{\mathrm{age}}+\beta_{3}\times x_{\mathrm{sex}}+\beta_{4}\times x_{\mathrm{edu}}\\ \;+\beta_{5}\times x_{\mathrm{hyp}}+\beta_{6}\times x_{\mathrm{dia}}+\beta_{7}\times x_{\text{ETIV}} \end{array}$$

where *Y* was the dependent variable, including cognitive indicators or ophthalmic biometrics; $x_{\mathrm{obj}}$ represented FALFF, ReHo, or gray matter cortical thickness; $x_{\mathrm{age}}$, $x_{\mathrm{sex}}$, $x_{\mathrm{edu}}$, $x_{\mathrm{hyp}}$, $x_{\mathrm{dia}}$ and $x_{\text{ETIV}}$ represented covariates, including age, sex, years of education, hypertension status, diabetes status, and ETIV, respectively; and $\beta_{0}$ to $\beta_{7}$ were model parameters. Statistical significance was set at *p* < 0.05 with FWE correction for fMRI and *p* < 1/62 (Bonferroni‐corrected) for structural analyses.

## 3 Results

### 3.1 Demographic Characteristics

The demographic and neuropsychological data of the HC, SCD, MCI, and AD groups are shown in Table 1. There were no significant differences in age, sex, and education between the four groups (*p* = 0.797, *p* = 0.262, and *p* = 0.481). Significant differences were found between the groups for the scores of MMSE, MoCA, BNT, CDT, BDST, TMT‐A, and TMT‐B.

**TABLE 1:** Demographic and behavioral characteristics of the participants.

Demographics	NC	SCD	MCI	AD	*χ* ^2^/*F*/K–W	*p*‐Value
Sex, m/f	5/11	7/28	8/10	3/4	3.996	0.262
Age, y, (SD)	66.63 (4.559)	65.91 (4.161)	66.22 (5.264)	64.57 (5.192)	0.339	0.797
Education, y, M (P25, P75)	12 (12, 15)	12 (12, 15)	12 (9, 12)	12 (9, 12)	7.272	0.481
Hypertension, yes/no	2/14	15/20	5/13	1/6	5.922	0.115
Diabetes, yes/no	2/14	2/33	3/15	0/7	2.625	0.453
MMSE, M (P25, P75)	29 (27, 29)	29 (28, 30)	28 (25, 29)	17 (15, 19)	27.309	< 0.001
MoCA, M (P25, P75)	25.5 (24.25, 27)	26 (24, 28)	23 (19.75, 25)	14 (12, 15)	32.679	< 0.001
BNT, M (P25, P75)	26 (23.5, 27.75)	27 (26, 28)	23.5 (21, 27)	21 (20, 23)	23.034	< 0.001
CDT, M (P25, P75)	4 (3.25, 4)	4 (4, 4)	4 (4, 4)	1 (1, 4)	12.486	0.006
BDST, M (P25, P75)	5 (4.25, 5)	5 (3, 6)	4 (3, 5)	4 (3, 4)	10.572	0.014
TMT‐A, M (P25, P75)	43.5 (37.25, 62.25)	45 (35, 52)	56.5 (47, 68.75)	120 (74, 150)	26.814	< 0.001
TMT‐B, M (P25, P75)	68.5 (52.25, 104.5)	67 (51, 80)	89.5 (75.75, 135)	169 (141, 235.5)	19.618	< 0.001

*Note*: Data are expressed as mean (SD) or percentage (%).

Abbreviations: *χ* ^2^, Chi‐square test; AD, Alzheimer's disease; BDST, Backward‐Digit Span Test; BNT, Boston Naming Test; CDT, Clock Drawing Test; f, Female; *F*, F‐test; K–W, Kruskal–Wallis test; m, male; M, median; (P25, P75), interquartile range; MCI, mild cognitive impairment; MMSE, Mini‐Mental State Examination; MoCA, Montreal Cognitive Assessment; NC, normal control; SCD, subjective cognitive decline; SD, standard deviation; TMT‐A, Trail Making Test‐part A; TMT‐B, Trail Making Test‐part B.

### 3.2 Cognition–Eye–Brain Function Connection

The two‐step logistic regression model revealed no significant cognition–eye–brain function connection in SCD and HC groups (*p*_FWE_ > 0.05, *p*‐values detailed in Tables S1–S10).

**FIGURE 2:**
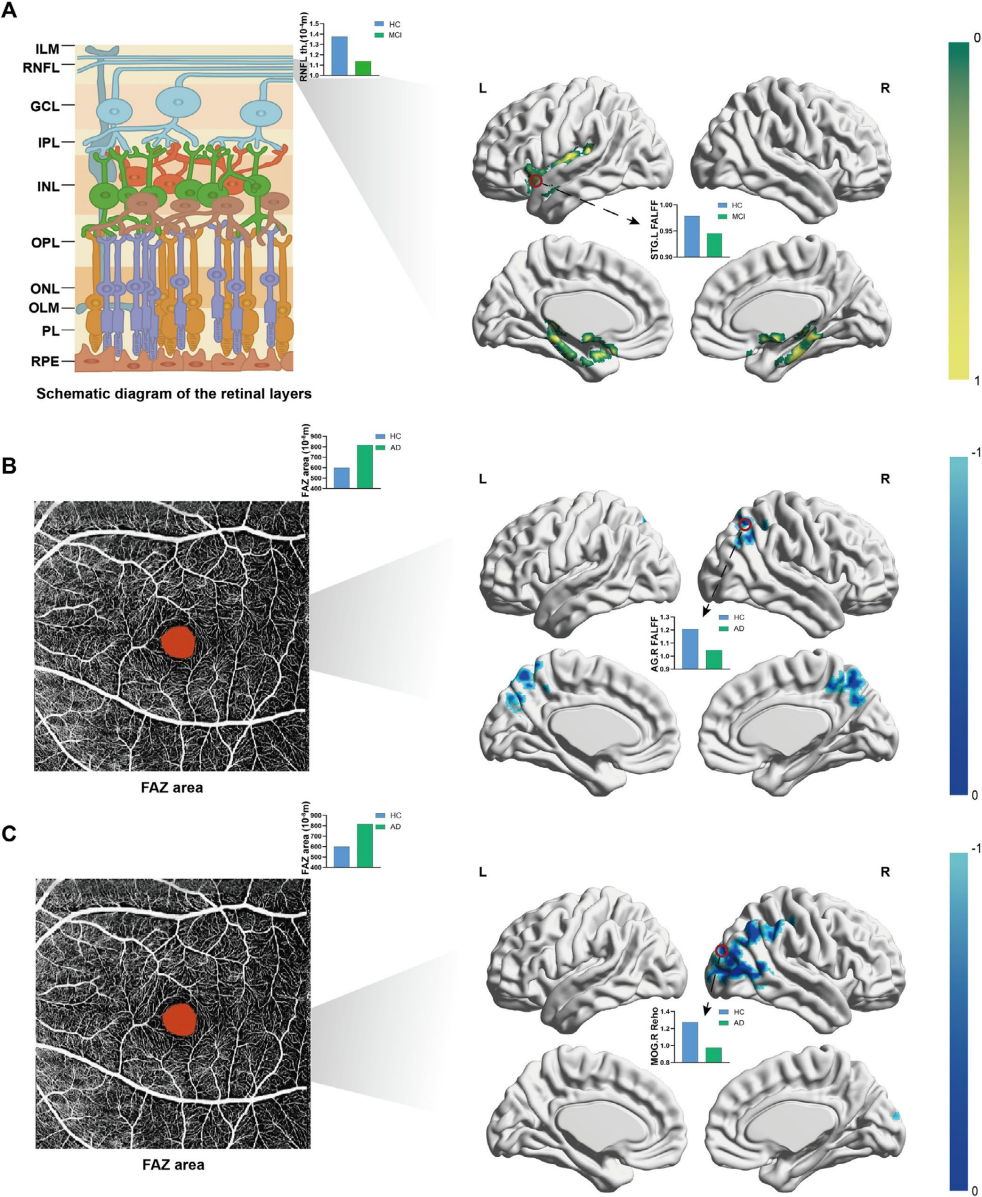
Cognition–eye–brain function connection. (A) In MCI and HC groups, among the brain regions significantly correlated with the BDST score, those where the FALFF value was positively correlated with RNFL thickness are colored in green and yellow, with the left STG as the peak location (red circle). (B) In AD and HC groups, among the brain regions significantly correlated with TMT‐A score, those where the FALFF values were negatively correlated with FAZ area are highlighted in blue, with the right AG as the peak. (C) In AD and HC groups, among the brain regions significantly correlated with TMT‐B score, those where the ReHo value were negatively correlated with FAZ area are highlighted in blue, with the MOG as the peak location. AG, angular gyrus; BDST, Backward‐Digit Span Test; FALFF, fractional amplitude of low frequency fluctuations; FAZ, foveal avascular zone; L, left; MOG, middle occipital gyrus; R, right; RNFL, retinal nerve fiber layer; ReHo, regional homogeneity; STG, superior temporal gyrus; TMT‐A, Trail Making Test‐part A; TMT‐B, Trail Making Test‐part B.

When analyzing the MCI and HC groups together, among the brain regions significantly correlated with the BDST score, those where the FALFF values were positively correlated with RNFL thickness are highlighted in Figure 2A. The left superior temporal gyrus (STG) serves as the peak location of this association. Hence, compared to the HC group, the FALFF values in temporal regions, especially the left STG, were decreased in the MCI group, along with significantly decreased RNFL thickness.

When analyzing the AD and HC groups together, among the brain regions where the FALFF value was significantly associated with the TMT‐A score, those negatively correlated with the FAZ area are highlighted in Figure 2B. The right angular gyrus (AG) serves as the peak location. Similarly, regions where the ReHo value was significantly correlated with the TMT‐B score showed a negative correlation with the FAZ area in Figure 2C, with the right middle occipital gyrus (MOG) as the peak location. Hence, in patients with AD, decreased FALFF in parietal regions, especially in the right AG, was significantly associated with an increase in the FAZ area. Similarly, reduced ReHo values in occipital regions, especially in the right MOG, were significantly associated with an enlarged FAZ area.

### 3.3 Cognition–Eye–Brain Structure Connection

**FIGURE 3:**
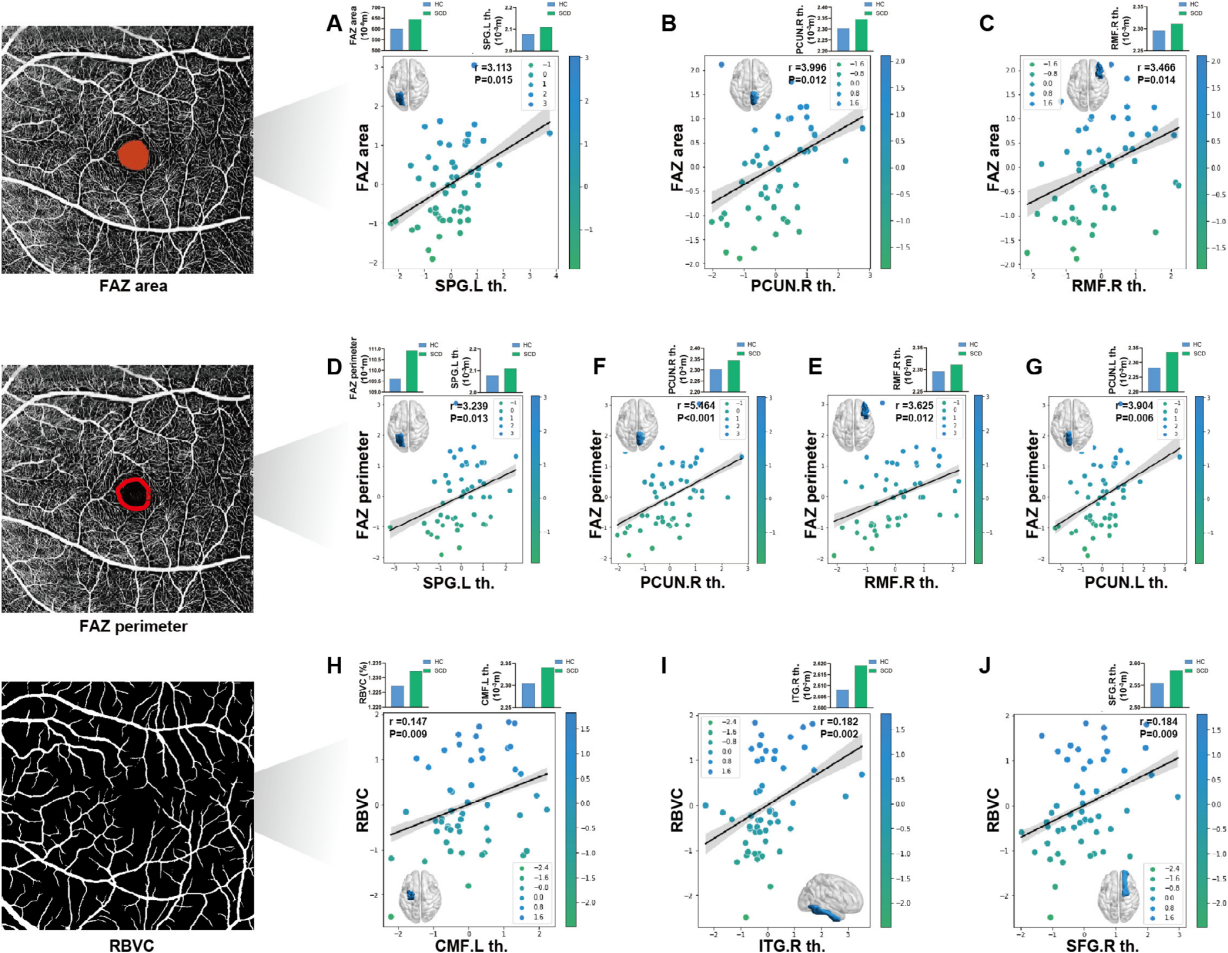
Cognition–retinal vessel–brain structure correlation in SCD and HC group. Among the brain regions that significantly correlated with neuropsychological scores, those whose cortical thickness were significantly correlated with FAZ area are shown in (A)–(C). (D)–(G) The correlation with FAZ perimeter, (H)–(J) for RBVC. CMF, caudal middle frontal; FAZ, foveal avascular zone; ITG, inferior temporal gyrus; L, left; PCUN, precuneus; R, right; RBVC, retinal blood vessel curve; RMF, rostral middle frontal; SFG, superior frontal gyrus; SPG, superior parietal gyrus.

**FIGURE 4:**
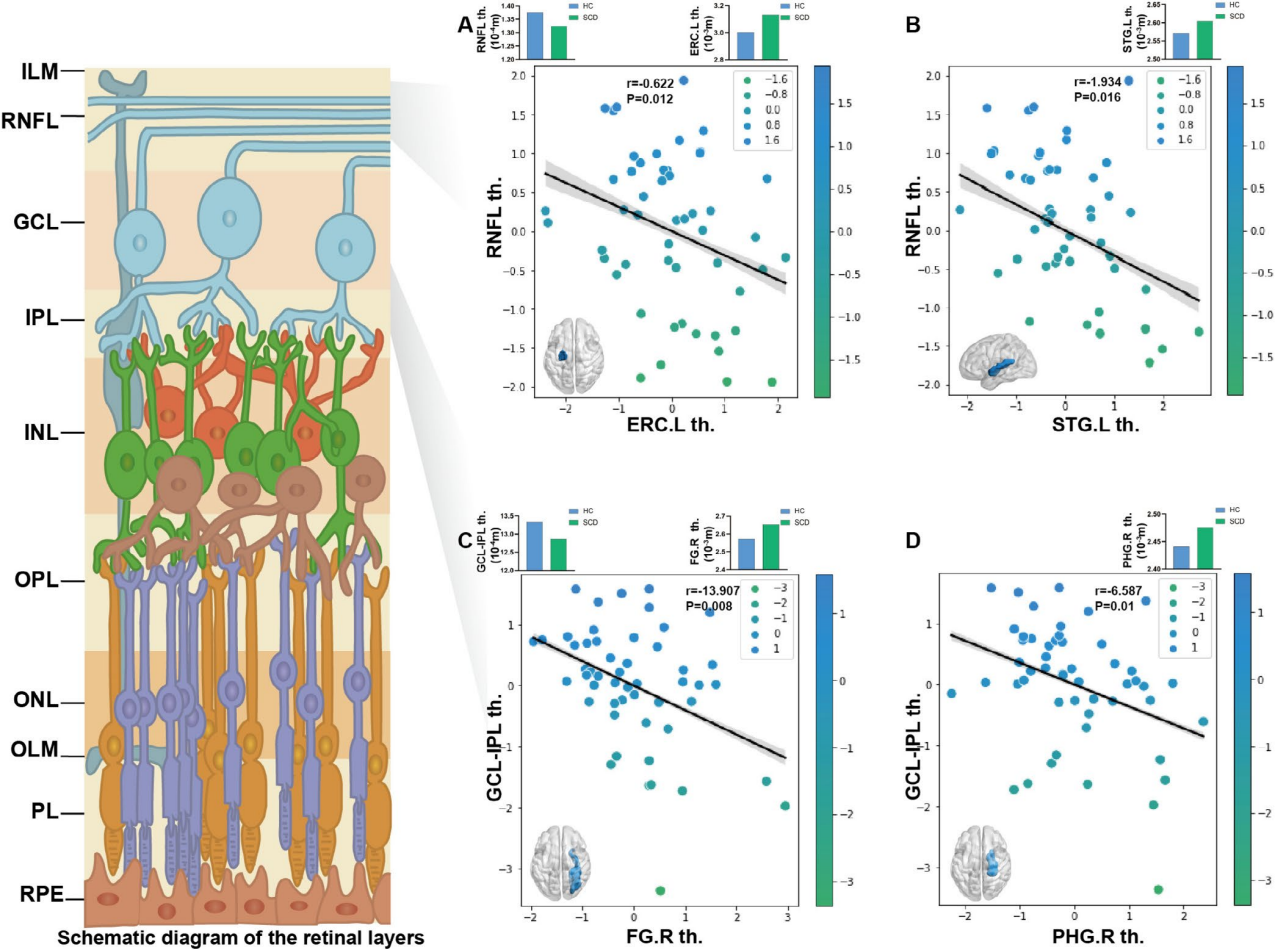
Cognition–retinal thickness–brain structure correlation in SCD and HC group. Among the brain regions that significantly correlated with neuropsychological scores, those whose cortical thickness were significantly correlated with RNFL thickness are shown in (A,B) and (C,D) for GCL‐IPL thickness. ERC, entorhinal cortex; FG, fusiform gyrus; GCL, ganglion cell layer; IPL, inner plexiform layer; L, left; PHG, parahippocampal gyrus; R, right; RNFL, retinal nerve fiber layer; STG, superior temporal gyrus.

In the SCD and HC group, the two‐step logistic regression model revealed significant cognition–eye–brain structure correlations for the MMSE, MoCA, BNT, CDT, TMT‐A, and TMT‐B scores at the ROI level. Specifically, the cortical thickness of the left superior parietal gyrus (SPG), right precuneus (PCUN), and right rostral middle frontal (RMF) was significantly positively correlated with both the FAZ area (*r* = 3.113; *r* = 3.996; and *r* = 3.466) (Figure 3A–C) and FAZ perimeter (*r* = 3.239; *r* = 5.464; and *r* = 3.625) (Figure 3D–F), while the cortical thickness of the left PCUN was only significantly positively correlated with the FAZ perimeter (*r* = 3.904) (Figure 3G). Additionally, the cortical thickness of the left caudal middle frontal (CMF), right inferior temporal gyrus (ITG), and right superior frontal gyrus (SFG) was significantly positively correlated with the RBVC (*r* = 0.147; *r* = 0.182; and *r* = 0.184) (Figure 3H–J). Moreover, the cortical thickness of the left entorhinal cortex (ERC) and left STG was significantly negatively correlated with RNFL thickness (*r* = −0.622; *r* = −1.934) (Figure 4A,B). The cortical thickness of the right fusiform gyrus (FG) and the right parahippocampal gyrus (PHG) was significantly negatively correlated with the GCL‐IPL thickness (*r* = −13.907; *r* = −6.587) (Figure 4C,D).

**FIGURE 5:**
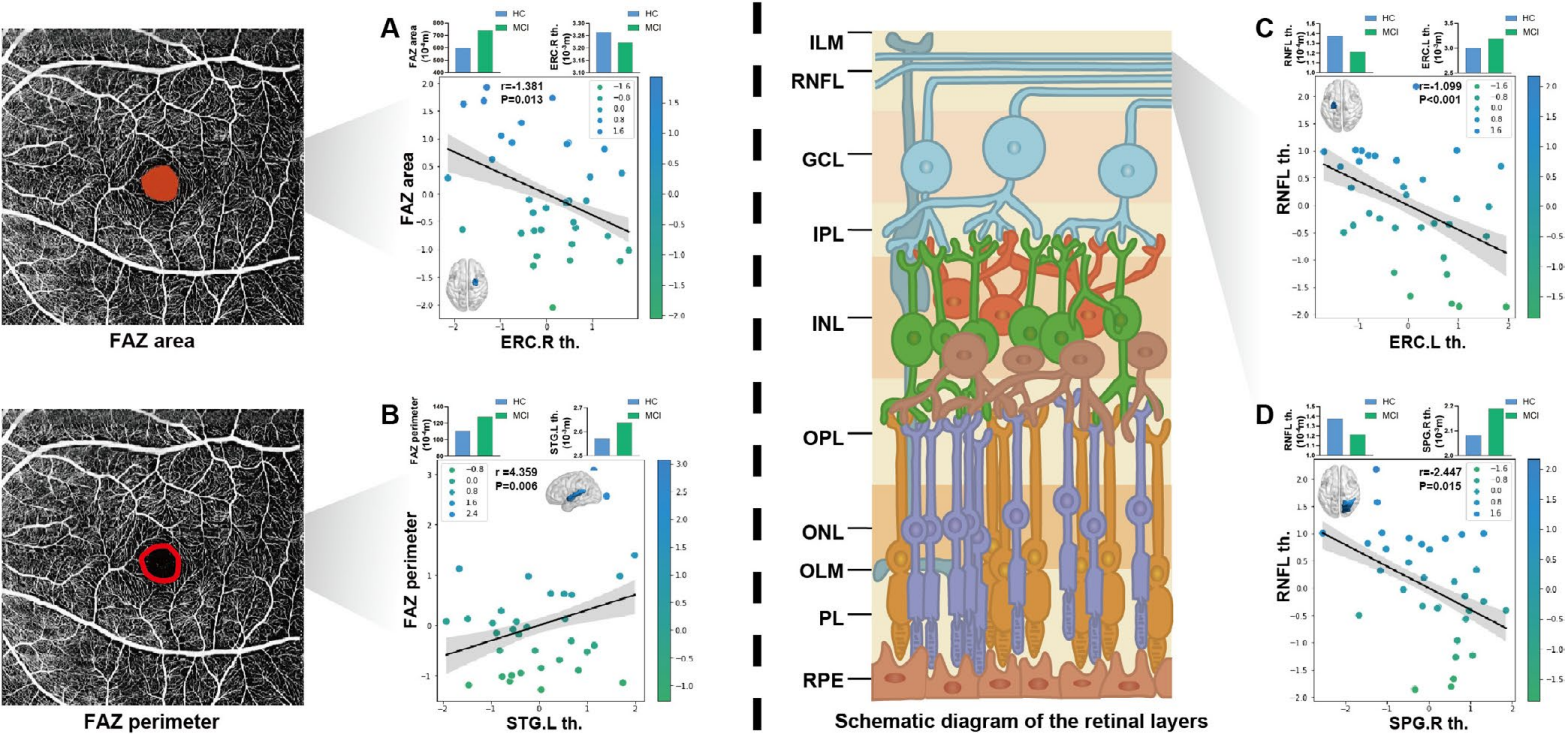
Cognition–eye–brain structure correlation in MCI and HC group. (A) Among the brain regions significantly correlated with neuropsychological scores, the cortical thickness of the right ERC was negatively correlated with the FAZ area. (B) The left STG was positively correlated with the FAZ perimeter. (C,D) The cortical thickness of the left ERC and the right SPG were all negatively correlated RNFL thickness. ERC, entorhinal cortex; FAZ, foveal avascular zone; L, left; R, right; RNFL, retinal nerve fiber layer; SPG, superior parietal gyrus; STG, superior temporal gyrus.

When analyzing the MCI and HC groups together, among the brain regions significantly correlated with MMSE, MoCA, CDT, and TMT‐A scores, the cortical thickness of the right ERC was significantly negatively correlated with the FAZ area (*r* = −1.381) (Figure 5A). The left STG thickness was significantly positively correlated with the FAZ perimeter (*r* = 4.359) (Figure 5B). There was no significant correlation between the cortical thickness and RBVC (*p* > 0.05, *p*‐values detailed in Tables S11 and S12). The cortical thickness of the left ERC and the right SPG was significantly negatively correlated with RNFL thickness (*r* = −1.099; *r* = −2.447) (Figure 5C,D).

**FIGURE 6:**
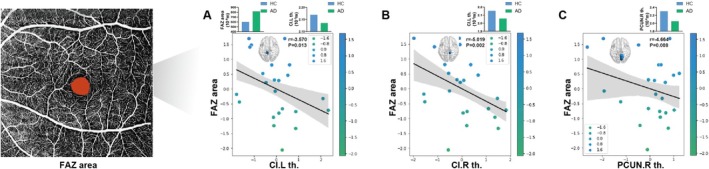
Cognition–eye–brain structure correlation in AD and HC group. Among the brain regions significantly correlated with neuropsychological scores, the cortical thickness of the left CI (A), the right CI (B) and the right PCUN (C) were all negatively correlated with the FAZ area. CI, cingulate isthmus; FAZ, foveal avascular zone; L, left; PCUN, precuneus; R, right.

When analyzing the AD and HC groups together, among the brain regions significantly correlated with MMSE, MoCA, CDT, DST, TMT‐A, and TMT‐B scores, the cortical thickness of the left cingulate isthmus (CI), the right CI, and the right PCUN was significantly negatively correlated with the FAZ area (*r* = −3.57; *r* = −5.019; and *r* = −4.664) (Figure 6A–C). There was no significant correlation between the cortical thickness and RBVC, nor with the thickness of the RNFL or GCL‐IPL (*p* > 0.05, *p*‐values detailed in Tables S13–S18).

## 4 Discussion

Using multimodal imaging and a two‐step linear regression model, this study offers insights into cognition–eye–brain connections across ADS disorders. Specifically, we found enlarged FAZ area/perimeter, increased RBVC, and thinned RNFL/GCL‐IPL in ADS. Significant correlations between brain function (FALFF and ReHo) and retinal biometrics were found in MCI and AD groups, but not in the SCD group. However, we observed widespread cortical thickening in the SCD group, which correlated with changes in retinal biomarkers, suggesting early structural adaptations of the brain.

In line with prior studies [17, 24], this study demonstrated enlarged FAZ areas and perimeters in ADS. Previous studies have shown that in AD patients, Aβ plaques, fibrillary tau, and neuroinflammation are present in brain regions and the retina [13, 28]. The widespread accumulation of Aβ exerts pressure on the retinal vascular network [29], causing insufficient blood supply and ischemia, reflected by an enlargement of the FAZ. In the SCD group, we found increased RBVC as compared to the HC group. Previous studies showed that patients with diabetes and hypertension had reduced retinal vascular density and increased vascular tortuosity [30, 31]. Patients with ADS disorders also exhibit decreased retinal vascular density [11], with Aβ deposition occurring in the retinal vessel walls, surrounding areas, and along the vessels [29]. These findings suggested that impaired Aβ deposition and clearance may damage retinal blood vessels, reducing retinal vascular density [32]. Although there is a lack of research on retinal vascular tortuosity changes in AD, we speculated that Aβ deposition may also make the vessels more twisted. The current study revealed a relationship between RBVC and brain structure in SCD patients, providing perspectives for future studies of retinal vascular changes in ADS disorders.

These results indicate that GCL‐IPL and RNFL are generally thinned across ADS disorders. Recent studies have found significant associations between AD diagnosis and retinal thinning, particularly in the inner retinal layers [11, 12]. The GCL‐IPL contains retinal ganglion cell (RGC) bodies, and the RNFL is primarily formed by their axons [33]. Postmortem studies in AD patients have shown RGC loss and axonal degeneration [34, 35]. Additionally, subtle retinal thickness changes in the macula are present in SCD and may correlate with Aβ accumulation [36]. These alterations in GCL‐IPL and RNFL thickness are likely related to RGC loss, axonal degeneration, and retinal Aβ deposition.

In terms of brain function, we found that decreased FALFF in temporal regions (especially the left STG) in MCI patients and occipital regions (especially the right AG) in AD patients were linked to reduced RNFL thickness and increased FAZ areas. In AD patients, reduced ReHo values in the right MOG were also associated with an increase in the FAZ area. In the current study, the decreases of FALFF and ReHo in AD and MCI were closely connected to FAZ area enlargement and RNFL thinning. A recent study revealed that structural and microvascular changes of the retina were associated with neural changes in the brain as measured by functional connectivity density in patients with neuromyelitis optica spectrum disorder (NMOSD) [37]. A population‐based prospective epidemiological study has indicated that thinner RNFL around the optic disc at baseline was significantly associated with an increased risk of dementia [38]. The connection of brain function and ophthalmic biometrics could thus provide new evidence on brain–eye mechanisms in ADS disorders. However, no significant cognition–eye–brain function connection was observed in the SCD group in our study.

In contrast, brain structure showed stronger connections with retinal biometrics in SCD relative to brain function. Specifically, the SCD group showed widespread cortical thickening, correlating with changes in several retinal biometrics, including increased FAZ area/perimeter, RBVC, and RNFL thinning. Notably, thickening in the right FG and right PHG was associated with reduced GCL‐IPL thickness, which was not observed in AD or MCI patients. This may suggest that brain structure is more vulnerable than brain function in prodromal AD [39]. Using amyloid PET imaging for pathological stratification, Imabayashi et al. observed an increase in the medial temporal lobe volume in preclinical AD patients [8]. However, another study found that while SCD exhibited higher Aβ accumulation levels, they did not show cortical atrophy [7]. This supports the hypothesis that the impact of Aβ on brain structure may follow a nonlinear dynamic pattern: initial low levels of Aβ could trigger compensatory thickening, whereas excessive accumulation may eventually lead to degenerative atrophy [40]. Thus, we speculate that cortical thickening in SCD may represent a compensatory response to early cognitive changes. Due to its high metabolic demands and oxygen consumption, the GCL‐IPL layer is particularly vulnerable to hypoxia and insufficient blood perfusion [41]. The vulnerability of GCL‐IPL makes it potentially closely correlated with brain structure in SCD, the earliest clinical manifestation of ADS disorders.

In AD and MCI patients, the atrophy of bilateral CI, right PCUN, and right ERC was associated with FAZ area enlargement. Previous studies have shown reduced cerebral perfusion in the temporal lobe and PCUN in AD patients, leading to atrophy in these regions [42, 43]. In AD patients, previous work has revealed that there is a correlation between the decrease in retinal vascular density and cerebral ischemia [44]. The reduction in retinal vascular density could lead to ischemic conditions in the retina, which are reflected by an enlargement of the FAZ area. This could explain the correlation between FAZ area and brain region changes observed in AD and MCI patients.

In MCI patients, thickening of the left STG, left ERC, and right SPG was associated with RNFL thinning. In this context, previous studies found correlations between RNFL thickness and temporal lobe volume in cognitively impaired individuals and normal older adults [45, 46]. Sung et al. demonstrated a widespread correlation between retinal and brain structure in patients with Parkinson's disease [47]. The visual association cortex in the temporo‐parietal region is particularly vulnerable to the deposition of NFTs and Aβ [28], which may disrupt the connections within the visual pathways and lead to retrograde optic nerve degeneration. This could explain the correlation between the temporo‐parietal region and RNFL structural changes in MCI patients.

## 5 Limitations

This study had a cross‐sectional design and a small sample size, particularly for the AD group. This is primarily due to the strict exclusion criteria and the need for ocular measurement compatibility. Second, the lack of Aβ or tau‐related biomarkers prevented analysis of their relationship with ocular measurements; thus, we cannot conclude whether changes observed in ophthalmic examinations are specific to AD. Third, while the DKT atlas was used for brain segmentation, the structural and functional heterogeneity of brain subregions may have diluted potential associations. Future research could use repeated ocular and brain imaging scans with a longitudinal study design and incorporate subregion‐level analysis to explore the causal relationships between these factors.

## 6 Conclusion

Using multimodal imaging, we found that brain function and structure were significantly correlated with cognition and retinal biometrics across ADS disorders. Notably, brain structure may exhibit pronounced cognition–eye–brain connections in SCD, while brain function did not. Our findings emphasize the clinical potential of non‐invasive multimodal biomarkers, such as retinal and brain structure/function measures, for early diagnosis and monitoring of neurodegenerative diseases. Specifically, the correlations in SCD could help identify at‐risk individuals before cognitive decline. Furthermore, combined retinal and brain imaging also offers a powerful tool for risk stratification, enabling timely interventions to prevent progression to AD.

## Author Contributions

Min Zhang, Xiaoying Tang, Yuanyuan Qin, and Wenzhen Zhu – designed the study. Su Yan, Rong Gao, and Renpuchi Ci – collected data. Yan Shi, Tingting Shen, Jianwen Liang, Tianyunxi Wei, and Yijin Huang – methodology. Yan Shi – original draft. Xiaoying Tang, Yuanyuan Qin, and Ning Zheng – reviewed and revised the manuscript. The order of co‐first authors was assigned by the contribution to this study.

## Ethics Statement

The study was approved by the ethics committee of Tongji Hospital, Tongji Medical College, and the Huazhong University of Science and Technology (TJ‐IRB202401097).

## Conflicts of Interest

The authors declare no conflicts of interest.

## Patient Statement

Written informed consent was obtained from all participants.

## Supporting information


**TABLE S1:** There was no significant correlation between the cognition–FAZ area and FALFF in HC and SCD patients.
**TABLE S2:** There was no significant correlation between the cognition–FAZ perimeter and FALFF in HC and SCD patients.
**TABLE S3:** There was no significant correlation between the cognition–GCL‐IPL and FALFF in HC and SCD patients.
**TABLE S4:** There was no significant correlation between the cognition–RNFL and FALFF in HC and SCD patients.
**TABLE S5:** There was no significant correlation between the cognition–RBVC and FALFF in HC and SCD patients.
**TABLE S6:** There was no significant correlation between the cognition–FAZ area and ReHo in HC and SCD patients.
**TABLE S7:** There was no significant correlation between the cognition–FAZ perimeter and ReHo in HC and SCD patients.
**TABLE S8:** There was no significant correlation between the cognition–GCL‐IPL and ReHo in HC and SCD patients.
**TABLE S9:** There was no significant correlation between the cognition–RNFL and ReHo in HC and SCD patients.
**TABLE S10:** There was no significant correlation between the cognition–RBVC and ReHo in HC and SCD patients.
**TABLE S11:** There was no significant correlation between cortical thickness of the left hemisphere and RBVC in HC and MCI.
**TABLE S12:** There was no significant correlation between cortical thickness of the right hemisphere and RBVC in HC and MCI.
**TABLE S13:** There was no significant correlation between cortical thickness of the left hemisphere and RBVC in HC and AD.
**TABLE S14:** There was no significant correlation between cortical thickness of the right hemisphere and RBVC in HC and AD.
**TABLE S15:** There was no significant correlation between cortical thickness of the left hemisphere and RNFL in HC and AD.
**TABLE S16:** There was no significant correlation between cortical thickness of the right hemisphere and RNFL in HC and AD.
**TABLE S17:** There was no significant correlation between cortical thickness of the left hemisphere and GCL‐IPL in HC and AD.
**TABLE S18:** There was no significant correlation between cortical thickness of the right hemisphere and GCL‐IPL in HC and AD.

## Data Availability

The original datasets in this study are available from the corresponding author upon reasonable request.
