# Supplementary material for: Cognition–Eye–Brain Connection in Alzheimer's Disease Spectrum Revealed by Multimodal Imaging
Source: J Magn Reson Imaging. 2025 Jun 29;62(4):1119–30. doi: 10.1002/jmri.70003 (PMC12435140; doi:10.1002/jmri.70003)
Supplement: Supplementary file 1 — TABLE S1: There was no significant correlation between the cognition–FAZ area and FALFF in HC and SCD patients. TABLE S2: There was no significant correlation between the cognition–FAZ perimeter and FALFF in HC and SCD patients. TABLE S3: There was no significant correlation between the cognition–GCL‐IPL and FALFF in HC and SCD patients. TABLE S4: There was no significant correlation between the cognition–RNFL and FALFF in HC and SCD patients. TABLE S5: There was no significant correlation between the cognition–RBVC and FALFF in HC and SCD patients. TABLE S6: There was no significant correlation between the cognition–FAZ area and ReHo in HC and SCD patients. TABLE S7: There was no significant correlation between the cognition–FAZ perimeter and ReHo in HC and SCD patients. TABLE S8: There was no significant correlation between the cognition–GCL‐IPL and ReHo in HC and SCD patients. TABLE S9: There was no significant correlation between the cognition–RNFL and ReHo in HC and SCD patients. TABLE S10: There was no significant correlation between the cognition–RBVC and ReHo in HC and SCD patients. TABLE S11: There was no significant correlation between cortical thickness of the left hemisphere and RBVC in HC and MCI. TABLE S12: There was no significant correlation between cortical thickness of the right hemisphere and RBVC in HC and MCI. TABLE S13: There was no significant correlation between cortical thickness of the left hemisphere and RBVC in HC and AD. TABLE S14: There was no significant correlation between cortical thickness of the right hemisphere and RBVC in HC and AD. TABLE S15: There was no significant correlation between cortical thickness of the left hemisphere and RNFL in HC and AD. TABLE S16: There was no significant correlation between cortical thickness of the right hemisphere and RNFL in HC and AD. TABLE S17: There was no significant correlation between cortical thickness of the left hemisphere and GCL‐IPL in HC and AD. TABLE S18: There was no significant c [file JMRI-62-1119-s001.docx]

**Table S1. There was no significant correlation between the cognition-FAZ area- FALFF in HC and SCD patients.**

| X (mm) | Y (mm) | Z (mm) | P_FWE_ |
| --- | --- | --- | --- |
| 42 | -45 | -33 | 0.088713 |
| -36 | 27 | -24 | 0.088985 |
| -54 | -66 | 18 | 0.109261 |
| -57 | -66 | 18 | 0.111269 |
| 45 | -12 | 57 | 0.111698 |
| 45 | -9 | 57 | 0.113865 |
| -57 | -63 | 18 | 0.11508 |
| 39 | -48 | -30 | 0.118057 |
| -36 | 24 | -24 | 0.126897 |
| 24 | -21 | 72 | 0.132508 |
| -9 | -99 | 15 | 0.136137 |
| 42 | -42 | -30 | 0.13867 |
| -60 | -60 | 18 | 0.146129 |
| 42 | -45 | -27 | 0.159673 |
| 42 | -42 | -27 | 0.159886 |
| -57 | -57 | 15 | 0.188858 |
| 0 | -75 | -18 | 0.190108 |
| 39 | -45 | -36 | 0.19215 |
| 18 | -84 | -30 | 0.193659 |
| -42 | -42 | -24 | 0.195333 |
| 27 | -60 | -27 | 0.20531 |
| -60 | -27 | 3 | 0.22326 |
| -57 | -69 | 18 | 0.235165 |
| 30 | 39 | 45 | 0.241448 |
| 39 | -45 | -27 | 0.241992 |
| 39 | -42 | -30 | 0.242276 |
| 30 | 42 | 45 | 0.253029 |
| -6 | -99 | 9 | 0.253253 |
| 39 | -48 | 57 | 0.258461 |
| -57 | -60 | 15 | 0.259951 |
| -51 | -66 | 18 | 0.261531 |
| -12 | -78 | -15 | 0.263338 |
| 42 | -12 | 24 | 0.275221 |
| -60 | -63 | 18 | 0.285092 |
| 21 | -84 | -30 | 0.299775 |
| -39 | -42 | -24 | 0.304317 |
| 48 | -48 | -36 | 0.314072 |
| -54 | -63 | 18 | 0.319361 |
| -60 | -66 | 18 | 0.331963 |
| 21 | -81 | -30 | 0.338171 |
| 24 | -24 | 72 | 0.33979 |
| -57 | -54 | 18 | 0.368243 |
| 33 | 33 | -12 | 0.368539 |
| -3 | -72 | -15 | 0.375003 |
| -6 | -72 | -12 | 0.379703 |
| 0 | -78 | -21 | 0.383411 |
| -60 | -27 | 6 | 0.388176 |
| -3 | -75 | -18 | 0.393358 |
| 27 | -60 | -24 | 0.39769 |
| -60 | -69 | 18 | 0.398079 |
| -57 | -57 | 21 | 0.413749 |
| -54 | -69 | 18 | 0.419475 |
| 24 | -18 | 72 | 0.423608 |
| -6 | -99 | 15 | 0.426791 |
| -57 | -63 | 15 | 0.429905 |
| -3 | -78 | -18 | 0.433363 |
| 48 | -45 | -36 | 0.437205 |
| 24 | -84 | -30 | 0.440125 |
| 21 | -60 | -21 | 0.442876 |
| -6 | -72 | -15 | 0.443564 |
| 27 | -27 | -30 | 0.448477 |
| 36 | -48 | 57 | 0.457651 |
| -3 | -75 | -15 | 0.458 |
| 48 | -48 | -33 | 0.461887 |
| 30 | -60 | -27 | 0.466595 |
| 42 | -72 | -30 | 0.46865 |
| -24 | -90 | 27 | 0.47344 |
| 42 | -69 | -30 | 0.477243 |
| -54 | -63 | 15 | 0.483011 |
| -9 | -39 | 75 | 0.486821 |
| -21 | -87 | 27 | 0.48708 |
| 45 | -15 | 57 | 0.492465 |
| -9 | -72 | -15 | 0.499486 |
| -12 | -75 | -15 | 0.500024 |
| 24 | -60 | -24 | 0.500191 |
| 60 | 6 | 27 | 0.501742 |
| 42 | -12 | 57 | 0.502165 |
| 24 | -21 | 69 | 0.502958 |
| -39 | -39 | -24 | 0.504757 |
| -9 | -99 | 12 | 0.506189 |
| 24 | -18 | 69 | 0.508488 |
| 3 | -78 | -21 | 0.508601 |
| -3 | -72 | -12 | 0.514312 |
| 42 | -48 | -30 | 0.518231 |
| 39 | -42 | -33 | 0.522928 |
| 36 | -45 | -30 | 0.52305 |
| 48 | -45 | -33 | 0.530375 |
| -15 | -78 | -15 | 0.540252 |
| 21 | -24 | 72 | 0.546382 |
| 27 | -60 | -21 | 0.548435 |
| -54 | -45 | -9 | 0.548842 |
| -6 | -69 | -12 | 0.561491 |
| 42 | -9 | 57 | 0.574427 |
| 45 | -12 | 60 | 0.575559 |
| 39 | -48 | -33 | 0.580736 |
| 30 | -63 | -27 | 0.58429 |
| -6 | -99 | 12 | 0.584966 |
| -57 | -27 | 3 | 0.592333 |
| 27 | -72 | -51 | 0.594196 |
| 45 | -45 | -30 | 0.594709 |
| -54 | -66 | 15 | 0.6058 |
| 18 | -81 | -30 | 0.610101 |
| -51 | -48 | -9 | 0.614428 |
| 54 | -45 | 51 | 0.614553 |
| -60 | -24 | 3 | 0.614915 |
| 54 | -3 | 0 | 0.614936 |
| -24 | -66 | -51 | 0.617578 |
| 27 | -21 | 72 | 0.619435 |
| -9 | -36 | 78 | 0.62066 |
| 54 | -42 | 51 | 0.623019 |
| -12 | -39 | 75 | 0.625916 |
| -3 | -78 | -21 | 0.627248 |
| -9 | -99 | 9 | 0.653265 |
| 21 | -21 | 72 | 0.653736 |
| -63 | -63 | 18 | 0.655132 |
| 0 | -78 | -24 | 0.659018 |
| 12 | -69 | 57 | 0.66015 |
| 60 | -21 | 0 | 0.662093 |
| 24 | -81 | -30 | 0.665969 |
| -60 | -57 | 18 | 0.670376 |
| -63 | -30 | 9 | 0.671983 |
| 42 | -72 | -33 | 0.673158 |
| 24 | -78 | -9 | 0.681831 |
| 60 | 6 | 30 | 0.692852 |
| 39 | -69 | -54 | 0.695314 |
| 18 | -84 | 21 | 0.698884 |
| -60 | -30 | 6 | 0.704545 |
| -9 | -75 | -15 | 0.706837 |
| -51 | -48 | -12 | 0.715878 |
| 27 | -63 | -24 | 0.72603 |
| -6 | -75 | -15 | 0.726595 |
| -60 | -60 | 15 | 0.732443 |
| 21 | -27 | 72 | 0.746288 |
| 30 | -51 | -60 | 0.748903 |
| -12 | -99 | 15 | 0.750872 |
| 0 | -78 | -18 | 0.752088 |
| -54 | -48 | -12 | 0.755106 |
| -6 | -75 | -18 | 0.755758 |
| 3 | -75 | -21 | 0.756583 |
| 12 | -93 | 18 | 0.757137 |
| 30 | -48 | -57 | 0.758011 |
| 45 | -72 | -33 | 0.758808 |
| -9 | -39 | 78 | 0.761003 |
| 27 | -63 | -27 | 0.762338 |
| 45 | -6 | 57 | 0.764125 |
| -60 | -57 | 15 | 0.764405 |
| 27 | 42 | 42 | 0.765427 |
| 3 | -78 | -24 | 0.765469 |
| -24 | -90 | 24 | 0.765843 |
| -24 | -69 | -51 | 0.767762 |
| 12 | -72 | -51 | 0.76893 |
| 30 | 42 | 42 | 0.770843 |
| 24 | -27 | 72 | 0.772087 |
| 24 | -72 | -51 | 0.774018 |
| 39 | -42 | -27 | 0.777368 |
| -21 | -93 | 21 | 0.785017 |
| -57 | -24 | 3 | 0.785691 |
| 33 | -48 | -57 | 0.791659 |
| 36 | -72 | -54 | 0.792582 |
| 27 | -21 | 69 | 0.793047 |
| 15 | -72 | -51 | 0.794838 |
| -51 | -63 | 15 | 0.794964 |
| 36 | -45 | -33 | 0.801197 |
| -39 | -42 | -27 | 0.802752 |
| 30 | -51 | -57 | 0.803102 |
| 42 | -48 | -33 | 0.803419 |
| 21 | -81 | -27 | 0.807308 |
| 24 | -81 | -27 | 0.810464 |
| 33 | -33 | -36 | 0.814951 |
| -54 | -57 | 18 | 0.816062 |
| 39 | -66 | -54 | 0.816758 |
| -9 | -72 | -12 | 0.817101 |
| -6 | -78 | -18 | 0.821415 |
| 36 | -48 | -57 | 0.82273 |
| 0 | -81 | -24 | 0.8246 |
| 42 | -12 | 60 | 0.824787 |
| 21 | -81 | 39 | 0.825159 |
| 30 | -60 | -24 | 0.826774 |
| 42 | -69 | -33 | 0.831708 |
| 27 | -51 | -60 | 0.832358 |
| -24 | -87 | 27 | 0.832574 |
| 30 | -72 | -51 | 0.833077 |
| -51 | -63 | 18 | 0.84298 |
| 60 | 9 | 30 | 0.846475 |
| -54 | -60 | 18 | 0.847338 |
| 33 | -57 | -30 | 0.85245 |
| 0 | -75 | -21 | 0.857074 |
| -15 | -39 | 72 | 0.857805 |
| -63 | -33 | 9 | 0.85781 |
| -51 | -66 | 15 | 0.857929 |
| -21 | -87 | 30 | 0.858501 |
| 39 | -72 | -54 | 0.86389 |
| 54 | -48 | -27 | 0.864496 |
| -57 | -54 | 21 | 0.865107 |
| 42 | -42 | 36 | 0.866302 |
| 27 | -51 | -57 | 0.86759 |
| -24 | -63 | -54 | 0.867663 |
| 60 | -24 | 0 | 0.868739 |
| -42 | -45 | -24 | 0.870748 |
| 63 | -21 | 0 | 0.871916 |
| -57 | -72 | 18 | 0.875875 |
| -24 | -66 | -54 | 0.876116 |
| -18 | -93 | 21 | 0.883058 |
| -12 | -81 | -15 | 0.884984 |
| -60 | -63 | 15 | 0.885214 |
| -9 | -96 | 15 | 0.888049 |
| 24 | -60 | -21 | 0.889043 |
| 33 | -72 | -54 | 0.897085 |
| 18 | -69 | -51 | 0.899824 |
| -12 | -39 | 72 | 0.900144 |
| 9 | -93 | 18 | 0.902141 |
| -6 | -78 | -15 | 0.902744 |
| -9 | -78 | -15 | 0.903237 |
| -51 | -51 | -12 | 0.905322 |
| -21 | -90 | 24 | 0.905386 |
| -3 | -78 | -24 | 0.906161 |
| -15 | -93 | 21 | 0.906422 |
| 18 | -72 | -51 | 0.908672 |
| 33 | 42 | 45 | 0.910362 |
| -21 | -63 | -54 | 0.911496 |
| -6 | -102 | 15 | 0.912922 |
| -69 | -36 | 6 | 0.91469 |
| 18 | -78 | 45 | 0.917158 |
| 12 | -69 | -51 | 0.917224 |
| 36 | -69 | -54 | 0.917827 |
| -57 | -66 | 15 | 0.918695 |
| 15 | -69 | -51 | 0.919563 |
| 27 | -18 | 69 | 0.921029 |
| 36 | -54 | -57 | 0.922124 |
| -6 | -96 | 18 | 0.925037 |
| 45 | -45 | -33 | 0.927815 |
| 21 | -87 | 21 | 0.929787 |
| -60 | -66 | 15 | 0.929872 |
| 33 | -60 | -54 | 0.929902 |
| -21 | -90 | 27 | 0.931952 |
| 21 | -75 | 45 | 0.934436 |
| -6 | -96 | 9 | 0.934739 |
| 33 | -57 | -54 | 0.93475 |
| -66 | -48 | -18 | 0.93681 |
| -24 | -72 | -51 | 0.93696 |
| -48 | -66 | -36 | 0.937986 |
| 30 | -60 | -21 | 0.940134 |
| -6 | -99 | 18 | 0.942059 |
| 36 | -45 | -36 | 0.942213 |
| -6 | -99 | 6 | 0.944726 |
| 36 | -51 | 57 | 0.946292 |
| 42 | -75 | -54 | 0.946529 |
| 42 | -27 | 42 | 0.947476 |
| 30 | 39 | 42 | 0.947714 |
| 39 | -27 | 42 | 0.94843 |
| 27 | -84 | -45 | 0.948801 |
| 51 | -30 | 9 | 0.950632 |
| 36 | -72 | -51 | 0.950727 |
| 0 | -75 | -15 | 0.951275 |
| 51 | -45 | -36 | 0.953697 |
| -6 | -78 | -21 | 0.960299 |
| 27 | -69 | -51 | 0.960338 |
| 21 | -84 | 21 | 0.961002 |
| 48 | -12 | 57 | 0.961096 |
| 24 | -78 | -6 | 0.962316 |
| 27 | 42 | 45 | 0.963316 |
| -21 | -66 | -51 | 0.963784 |
| -3 | -78 | -15 | 0.964775 |
| -54 | -48 | -9 | 0.9688 |
| -12 | -72 | -15 | 0.971706 |
| 6 | -78 | -24 | 0.973186 |
| 42 | -9 | 60 | 0.973294 |
| 24 | -75 | -6 | 0.974197 |
| 60 | 9 | 27 | 0.974873 |
| 42 | -45 | -36 | 0.976274 |
| 45 | -69 | -33 | 0.977153 |
| -60 | -30 | 9 | 0.977713 |
| -45 | 27 | -21 | 0.978995 |
| -63 | -30 | 6 | 0.979116 |
| -27 | -69 | -51 | 0.979255 |
| 39 | -30 | 45 | 0.979847 |
| 63 | 6 | 39 | 0.979917 |
| 39 | -69 | -51 | 0.981108 |
| 57 | -45 | 51 | 0.981408 |
| 0 | -81 | -21 | 0.985453 |
| -12 | -99 | 12 | 0.985589 |
| -39 | -60 | 39 | 0.985691 |
| 60 | 3 | 30 | 0.986328 |
| -12 | -78 | -18 | 0.987015 |
| -27 | -72 | -51 | 0.987099 |
| 36 | -54 | -60 | 0.987477 |
| 12 | -72 | -48 | 0.987501 |
| -36 | 0 | -51 | 0.987711 |
| 21 | -87 | 24 | 0.987779 |
| 33 | -51 | -57 | 0.987968 |
| -3 | -69 | -12 | 0.988201 |
| 39 | -72 | -51 | 0.988987 |
| -42 | -39 | -24 | 0.990482 |
| 39 | -48 | 54 | 0.991545 |
| -36 | -75 | 3 | 0.992987 |
| 33 | -45 | -57 | 0.993202 |
| 27 | -78 | -9 | 0.994392 |
| -54 | -69 | 15 | 0.994558 |
| 36 | -48 | -54 | 0.994969 |
| 39 | -54 | 33 | 0.995846 |
| 57 | -42 | 51 | 0.99608 |
| 51 | -45 | -27 | 0.996988 |
| -30 | -72 | -51 | 0.997075 |
| 21 | -78 | -3 | 0.997289 |
| 27 | -24 | 72 | 0.998502 |
| 30 | -30 | -33 | 0.99863 |
| 60 | -21 | 3 | 0.999302 |
| -66 | -51 | -18 | 0.999306 |
| 30 | -60 | -54 | 0.999518 |
|  |  |  |  |

Notes: FAZ, Foveal Avascular Zone.

**Table S2. There was no significant correlation between the cognition-FAZ perimeter- FALFF in HC and SCD patients.**

| X (mm) | Y (mm) | Z (mm) | P_FWE_ |
| --- | --- | --- | --- |
| -60 | -27 | 6 | 0.05516 |
| -57 | -57 | 15 | 0.06357 |
| 42 | -45 | -33 | 0.074426 |
| -54 | -66 | 18 | 0.077822 |
| -60 | -60 | 18 | 0.098783 |
| -57 | -60 | 15 | 0.098996 |
| -57 | -63 | 18 | 0.099546 |
| -36 | 27 | -24 | 0.108394 |
| -60 | -30 | 6 | 0.125784 |
| -54 | -63 | 18 | 0.126175 |
| 39 | -45 | -30 | 0.126224 |
| -51 | -63 | 15 | 0.128564 |
| 27 | -27 | -30 | 0.143657 |
| 39 | -45 | -36 | 0.147394 |
| -60 | -63 | 18 | 0.15348 |
| -63 | -30 | 6 | 0.159496 |
| 42 | -72 | -33 | 0.175821 |
| -54 | -63 | 15 | 0.184606 |
| 30 | 42 | 45 | 0.20079 |
| 30 | 39 | 45 | 0.208993 |
| -57 | -66 | 18 | 0.217381 |
| 33 | 42 | 45 | 0.227947 |
| 33 | -48 | -57 | 0.231184 |
| 51 | -30 | 12 | 0.239598 |
| 18 | -78 | 45 | 0.246082 |
| -57 | -54 | 18 | 0.267199 |
| 33 | 30 | -12 | 0.270296 |
| -63 | -63 | 18 | 0.277228 |
| -57 | -27 | 3 | 0.283633 |
| -60 | -57 | 18 | 0.296907 |
| -3 | -81 | -42 | 0.304828 |
| -51 | -63 | 18 | 0.305867 |
| -60 | -57 | 15 | 0.31018 |
| 42 | -45 | -30 | 0.314567 |
| -63 | -30 | 9 | 0.318342 |
| 36 | -48 | -57 | 0.321223 |
| -69 | -21 | 6 | 0.329603 |
| -63 | -15 | 0 | 0.330651 |
| 54 | -3 | 0 | 0.330869 |
| 63 | 0 | 15 | 0.333514 |
| 27 | -30 | -30 | 0.336276 |
| -51 | -66 | 18 | 0.346485 |
| -60 | -60 | 15 | 0.348575 |
| -57 | -63 | 15 | 0.353689 |
| 51 | -33 | 12 | 0.3538 |
| -60 | -24 | 3 | 0.362987 |
| 54 | -30 | 12 | 0.393002 |
| -3 | -78 | -24 | 0.396717 |
| -3 | -78 | -21 | 0.407359 |
| 3 | -78 | -21 | 0.408987 |
| -54 | -60 | 18 | 0.409653 |
| -54 | -60 | 15 | 0.411677 |
| 0 | -78 | -21 | 0.414243 |
| 54 | -45 | 51 | 0.42011 |
| -36 | 24 | -24 | 0.420789 |
| 39 | -51 | -54 | 0.424296 |
| 48 | -30 | 12 | 0.426903 |
| 21 | -78 | 45 | 0.435936 |
| -63 | -21 | 3 | 0.436683 |
| -66 | -21 | 6 | 0.441217 |
| 39 | -42 | -33 | 0.444409 |
| 51 | -30 | 15 | 0.444503 |
| 33 | 39 | 45 | 0.44461 |
| 45 | -12 | 57 | 0.447472 |
| -60 | -30 | 9 | 0.449866 |
| -60 | -66 | 18 | 0.452554 |
| -57 | -54 | 15 | 0.456987 |
| 54 | -33 | 12 | 0.458853 |
| -57 | -57 | 21 | 0.460399 |
| -15 | -78 | -15 | 0.460653 |
| 45 | -9 | 57 | 0.462643 |
| -51 | -60 | 15 | 0.465113 |
| -63 | -27 | 6 | 0.466745 |
| -57 | -54 | 21 | 0.475225 |
| -63 | -12 | -3 | 0.47705 |
| -66 | -18 | 0 | 0.478357 |
| -12 | -51 | 75 | 0.478725 |
| 66 | 0 | 18 | 0.482181 |
| 30 | -48 | -57 | 0.483034 |
| -60 | -63 | 15 | 0.483241 |
| -6 | -69 | -12 | 0.484628 |
| -66 | -15 | 0 | 0.486684 |
| 63 | 0 | 18 | 0.513668 |
| -66 | -9 | -3 | 0.516745 |
| 0 | -78 | -18 | 0.516898 |
| -6 | -99 | 9 | 0.522945 |
| -3 | -81 | -21 | 0.52332 |
| 42 | -69 | -33 | 0.526419 |
| 33 | -33 | -36 | 0.531906 |
| 18 | -78 | 42 | 0.536203 |
| 36 | -51 | -57 | 0.539839 |
| -63 | -24 | 3 | 0.546219 |
| 33 | -51 | -57 | 0.550617 |
| 21 | -81 | 39 | 0.563337 |
| 36 | -54 | -57 | 0.564945 |
| 42 | -45 | -36 | 0.565358 |
| 57 | -30 | 12 | 0.568411 |
| -6 | -78 | -21 | 0.569102 |
| 36 | -51 | -54 | 0.571883 |
| 57 | -45 | 30 | 0.572629 |
| 21 | -78 | 42 | 0.573954 |
| 66 | 0 | 15 | 0.575699 |
| 48 | 30 | -18 | 0.579612 |
| -57 | -66 | 21 | 0.581512 |
| 30 | -51 | -60 | 0.584141 |
| 54 | -42 | 51 | 0.586178 |
| -63 | -18 | 0 | 0.588167 |
| -63 | -63 | 15 | 0.596504 |
| 42 | -75 | -33 | 0.600242 |
| 57 | -45 | 54 | 0.607226 |
| -57 | -27 | 6 | 0.608701 |
| -66 | -18 | 3 | 0.613049 |
| -3 | -81 | -27 | 0.614866 |
| 36 | -54 | -54 | 0.615406 |
| 60 | 6 | 30 | 0.615683 |
| -54 | -66 | 21 | 0.621677 |
| 54 | -30 | 9 | 0.630525 |
| -63 | -9 | 33 | 0.630658 |
| 0 | -81 | -24 | 0.634624 |
| -51 | -60 | 12 | 0.636056 |
| -54 | -45 | 15 | 0.637102 |
| 45 | -72 | -33 | 0.64206 |
| -60 | -54 | 15 | 0.642424 |
| 39 | -54 | -57 | 0.645344 |
| -3 | -78 | -18 | 0.649754 |
| -3 | -81 | -24 | 0.652481 |
| -39 | 24 | -24 | 0.655907 |
| 24 | -24 | 72 | 0.660099 |
| 45 | -69 | -33 | 0.661541 |
| -3 | -72 | -12 | 0.666302 |
| -69 | -18 | 0 | 0.66747 |
| 0 | -78 | -24 | 0.669667 |
| 30 | -51 | -57 | 0.670944 |
| 39 | -54 | -54 | 0.671052 |
| 36 | -54 | -60 | 0.676423 |
| 0 | -81 | -21 | 0.680058 |
| -6 | -72 | -12 | 0.683483 |
| 60 | 9 | 30 | 0.686456 |
| 42 | -72 | -30 | 0.687838 |
| -3 | -72 | -51 | 0.694528 |
| 42 | -54 | -57 | 0.696668 |
| -66 | -9 | 30 | 0.696945 |
| 27 | -51 | -60 | 0.698169 |
| -54 | -57 | 18 | 0.703555 |
| 39 | -51 | -57 | 0.704396 |
| 51 | -3 | 0 | 0.705542 |
| -66 | -18 | 6 | 0.706704 |
| -57 | -24 | 3 | 0.707532 |
| -69 | -18 | 3 | 0.708921 |
| 24 | -66 | 6 | 0.710337 |
| 39 | -69 | -51 | 0.711012 |
| 63 | 3 | 21 | 0.711586 |
| 57 | -45 | 51 | 0.712369 |
| 39 | -69 | -54 | 0.712591 |
| -9 | -75 | -51 | 0.716187 |
| 48 | -30 | 15 | 0.716676 |
| -63 | -9 | -3 | 0.716814 |
| 42 | -75 | 21 | 0.719168 |
| 57 | -33 | 12 | 0.721499 |
| 54 | -30 | 15 | 0.723943 |
| 63 | 12 | 9 | 0.725125 |
| -24 | -90 | 27 | 0.729827 |
| 0 | -75 | -18 | 0.730788 |
| -63 | -18 | 3 | 0.730835 |
| -54 | -45 | -9 | 0.731367 |
| 24 | -21 | 72 | 0.733933 |
| -24 | -66 | -51 | 0.738203 |
| 60 | -21 | 0 | 0.738371 |
| 60 | 12 | 9 | 0.739684 |
| 0 | -72 | -51 | 0.741989 |
| 63 | 3 | 15 | 0.751239 |
| 60 | -15 | 42 | 0.754633 |
| -66 | -3 | 21 | 0.756656 |
| 45 | -54 | -57 | 0.771523 |
| 39 | -48 | -57 | 0.776375 |
| -3 | -69 | -9 | 0.778823 |
| -66 | -21 | 3 | 0.781009 |
| -36 | -75 | 3 | 0.781184 |
| -66 | 0 | 21 | 0.789664 |
| 63 | -15 | 39 | 0.794593 |
| -54 | -69 | 18 | 0.795744 |
| 12 | -69 | 57 | 0.798157 |
| 33 | -48 | -54 | 0.800623 |
| 27 | -51 | -57 | 0.800728 |
| -60 | -15 | 3 | 0.801021 |
| -6 | -99 | 6 | 0.801308 |
| 60 | 6 | 27 | 0.806988 |
| 48 | -78 | -33 | 0.807885 |
| -60 | 15 | 18 | 0.810865 |
| 0 | -75 | -15 | 0.811622 |
| 42 | -51 | -57 | 0.812252 |
| -3 | -81 | -45 | 0.8143 |
| -60 | -27 | 9 | 0.815004 |
| 54 | -45 | 54 | 0.819789 |
| 33 | -54 | -57 | 0.819955 |
| -69 | -36 | 6 | 0.821041 |
| 30 | 42 | 42 | 0.822145 |
| -54 | -57 | 15 | 0.822744 |
| 63 | -21 | 0 | 0.82847 |
| 27 | -30 | -33 | 0.83206 |
| -60 | -69 | 21 | 0.833793 |
| 33 | -54 | -60 | 0.833805 |
| 24 | -63 | 6 | 0.83419 |
| 57 | -45 | 33 | 0.835986 |
| 63 | 3 | 18 | 0.83729 |
| -57 | -69 | 18 | 0.837476 |
| 21 | -78 | 39 | 0.837965 |
| 57 | -48 | 30 | 0.852998 |
| -27 | -69 | -51 | 0.854691 |
| -3 | -81 | -18 | 0.854759 |
| 33 | -45 | -57 | 0.855841 |
| 45 | -57 | -57 | 0.856553 |
| -21 | -87 | 27 | 0.857181 |
| 36 | -57 | -54 | 0.866388 |
| -66 | -6 | 24 | 0.870918 |
| 42 | -9 | 60 | 0.874865 |
| 42 | -69 | -30 | 0.876519 |
| -63 | -27 | 3 | 0.880362 |
| -12 | -75 | -51 | 0.882325 |
| 36 | -48 | -54 | 0.882405 |
| 45 | -75 | -33 | 0.884687 |
| -51 | -48 | -12 | 0.884881 |
| 51 | -33 | 15 | 0.894241 |
| -24 | -69 | -51 | 0.894614 |
| 54 | -33 | 15 | 0.897483 |
| 42 | -42 | -33 | 0.901635 |
| -27 | -72 | -51 | 0.904847 |
| -9 | -99 | 15 | 0.905475 |
| -6 | -69 | -9 | 0.908827 |
| -9 | -99 | 9 | 0.910665 |
| -15 | -78 | -18 | 0.915677 |
| -60 | -66 | 21 | 0.916283 |
| 60 | 3 | 27 | 0.917016 |
| -60 | -54 | 18 | 0.917053 |
| -3 | -84 | -42 | 0.917175 |
| 54 | -45 | 48 | 0.920025 |
| -66 | -6 | 27 | 0.920203 |
| 6 | -72 | -15 | 0.926231 |
| -6 | -75 | -51 | 0.927128 |
| 3 | -81 | -30 | 0.927342 |
| -3 | -81 | -36 | 0.930106 |
| 60 | 9 | 27 | 0.932555 |
| -63 | -51 | 15 | 0.933706 |
| 33 | -57 | -54 | 0.93378 |
| -63 | -48 | 15 | 0.937586 |
| 18 | -69 | 54 | 0.939478 |
| 42 | -57 | -57 | 0.939515 |
| 42 | -72 | 21 | 0.940051 |
| -12 | -51 | 78 | 0.941364 |
| -6 | -72 | -51 | 0.941826 |
| 39 | -66 | -54 | 0.942515 |
| -15 | 24 | 63 | 0.942564 |
| -63 | -6 | 33 | 0.942635 |
| 45 | -78 | -33 | 0.942886 |
| -3 | -72 | -9 | 0.944773 |
| -6 | -102 | 6 | 0.945514 |
| 0 | -78 | -15 | 0.947483 |
| -3 | -75 | -51 | 0.947601 |
| -24 | -51 | -57 | 0.947632 |
| 60 | -24 | 0 | 0.94774 |
| 24 | -78 | -6 | 0.951518 |
| 39 | -48 | -54 | 0.951757 |
| -12 | -54 | 75 | 0.9533 |
| -51 | -48 | -9 | 0.954562 |
| -51 | -45 | -6 | 0.955034 |
| 24 | -27 | 72 | 0.959647 |
| -60 | -69 | 18 | 0.960532 |
| 27 | -84 | -45 | 0.961055 |
| -6 | -72 | -9 | 0.963343 |
| 57 | -48 | 33 | 0.966318 |
| 54 | 12 | 18 | 0.966744 |
| 30 | -54 | -60 | 0.967129 |
| 39 | -57 | -54 | 0.970437 |
| -3 | -69 | -51 | 0.970717 |
| 18 | -81 | 45 | 0.970986 |
| -27 | -51 | -57 | 0.971705 |
| -63 | -60 | 18 | 0.974341 |
| -66 | -12 | 27 | 0.975108 |
| -60 | -48 | 15 | 0.976519 |
| 3 | -78 | -18 | 0.97841 |
| 39 | -45 | -27 | 0.978425 |
| 6 | -78 | -21 | 0.978481 |
| -45 | 27 | -21 | 0.978606 |
| 39 | -3 | 6 | 0.979023 |
| -12 | -78 | -18 | 0.980069 |
| 48 | -30 | 9 | 0.980157 |
| 57 | -42 | 51 | 0.980984 |
| -66 | -15 | 3 | 0.983361 |
| -3 | -72 | -15 | 0.98424 |
| 45 | 33 | -21 | 0.98442 |
| -9 | -72 | -51 | 0.985386 |
| 39 | -6 | 6 | 0.986397 |
| 42 | -45 | -27 | 0.986459 |
| 57 | 6 | -6 | 0.986688 |
| 18 | -84 | -30 | 0.987904 |
| -9 | -57 | -3 | 0.98842 |
| 57 | -30 | 9 | 0.99169 |
| -48 | -63 | 15 | 0.993466 |
| 42 | -9 | 57 | 0.994735 |
| 39 | -48 | 57 | 0.995133 |
| 51 | -30 | 9 | 0.995249 |
| -60 | -18 | 3 | 0.997713 |
| -6 | -78 | -24 | 0.998748 |
| -57 | -15 | 3 | 0.999103 |
| -18 | -54 | 75 | 0.999224 |
|  |  |  |  |

Notes: FAZ, Foveal Avascular Zone.

**Table S3. There was no significant correlation between the cognition-GCL-IPL - FALFF in HC and SCD patients.**

| X (mm) | Y (mm) | Z (mm) | P_FWE_ |
| --- | --- | --- | --- |
| 54 | 3 | 45 | 0.051603 |
| -9 | -6 | 72 | 0.055482 |
| 0 | 12 | 48 | 0.056846 |
| -6 | -84 | -39 | 0.059353 |
| -33 | 39 | 3 | 0.061582 |
| -63 | -39 | -3 | 0.072032 |
| -63 | -36 | -3 | 0.072739 |
| 27 | 6 | 69 | 0.075684 |
| -27 | 30 | 3 | 0.087685 |
| -30 | -36 | -51 | 0.089841 |
| 24 | 6 | 69 | 0.090929 |
| -15 | 60 | 6 | 0.105946 |
| 60 | -63 | 9 | 0.129413 |
| -3 | 15 | 45 | 0.132077 |
| 60 | -60 | 9 | 0.132246 |
| 12 | 48 | 42 | 0.144812 |
| 24 | -6 | -45 | 0.156512 |
| 24 | 9 | 69 | 0.173401 |
| -45 | -18 | -39 | 0.175755 |
| -42 | -3 | -30 | 0.180369 |
| 27 | 9 | 69 | 0.195435 |
| -3 | 12 | 48 | 0.203593 |
| -33 | -33 | -33 | 0.21618 |
| -6 | -87 | -39 | 0.217732 |
| -30 | -33 | -33 | 0.218084 |
| -33 | 18 | 6 | 0.21827 |
| 66 | -48 | -21 | 0.222412 |
| 69 | -48 | -15 | 0.226856 |
| 12 | 45 | 39 | 0.239675 |
| -63 | -39 | -6 | 0.240516 |
| -27 | -39 | -51 | 0.248445 |
| 57 | -63 | -12 | 0.253382 |
| -30 | 18 | 6 | 0.255233 |
| 3 | 24 | 57 | 0.256366 |
| -45 | -18 | -36 | 0.258064 |
| 6 | 39 | 39 | 0.267199 |
| 63 | -48 | -18 | 0.267519 |
| -66 | -36 | -3 | 0.272122 |
| -9 | -9 | 72 | 0.274907 |
| 66 | -51 | -18 | 0.277232 |
| 0 | 15 | 45 | 0.278192 |
| 60 | -60 | 12 | 0.279477 |
| 69 | -39 | -9 | 0.287043 |
| 0 | 12 | 51 | 0.302826 |
| 57 | -60 | 9 | 0.307586 |
| 30 | 30 | 3 | 0.312629 |
| -6 | -87 | -42 | 0.337045 |
| -63 | -36 | -6 | 0.346121 |
| 63 | -66 | 9 | 0.346322 |
| -27 | 45 | -21 | 0.35439 |
| 9 | 42 | 33 | 0.356151 |
| -33 | 0 | -30 | 0.360315 |
| 39 | -12 | -27 | 0.362259 |
| 69 | -45 | -18 | 0.363461 |
| -39 | -3 | -30 | 0.364081 |
| -9 | 60 | 24 | 0.370008 |
| -30 | -39 | -51 | 0.371456 |
| 0 | 15 | 48 | 0.37153 |
| -63 | -42 | -3 | 0.375974 |
| 54 | 3 | 42 | 0.378585 |
| -27 | 0 | -33 | 0.385506 |
| -66 | -36 | -6 | 0.386347 |
| -6 | -84 | -42 | 0.388555 |
| 66 | -39 | -9 | 0.388981 |
| -33 | 39 | 6 | 0.389646 |
| 66 | -45 | -18 | 0.391419 |
| 57 | -60 | -12 | 0.393362 |
| -33 | 3 | -33 | 0.397871 |
| 6 | 42 | 36 | 0.399338 |
| -42 | -45 | -54 | 0.410144 |
| 42 | -12 | -42 | 0.411394 |
| 69 | -42 | -9 | 0.41261 |
| -6 | -6 | 72 | 0.413344 |
| -33 | 3 | -30 | 0.417914 |
| 63 | -63 | 9 | 0.41966 |
| -63 | -42 | -6 | 0.422791 |
| -27 | -3 | -33 | 0.427184 |
| -42 | -9 | -51 | 0.436481 |
| -3 | 12 | 45 | 0.441626 |
| 72 | -42 | -9 | 0.450471 |
| -36 | 30 | 42 | 0.451381 |
| 27 | 0 | 72 | 0.454488 |
| -30 | 42 | -18 | 0.457701 |
| 21 | -81 | 54 | 0.457745 |
| -42 | -6 | -30 | 0.46187 |
| 0 | 63 | -18 | 0.471798 |
| -18 | 15 | 57 | 0.476461 |
| -33 | 0 | -33 | 0.481191 |
| -15 | 60 | 9 | 0.482004 |
| -39 | 9 | 9 | 0.484398 |
| 3 | 12 | 48 | 0.491563 |
| -27 | 45 | 36 | 0.491939 |
| -60 | -39 | -3 | 0.49802 |
| 9 | -27 | 42 | 0.498853 |
| -30 | 0 | -33 | 0.500609 |
| -6 | 69 | 0 | 0.500626 |
| 6 | -33 | 48 | 0.505001 |
| -36 | 0 | -30 | 0.505792 |
| -36 | 30 | 39 | 0.507147 |
| 24 | 6 | 66 | 0.514415 |
| 15 | 60 | -18 | 0.515381 |
| 12 | 48 | 39 | 0.522712 |
| 36 | -12 | -27 | 0.531346 |
| -30 | -30 | -33 | 0.536809 |
| -39 | -6 | -30 | 0.536913 |
| -33 | -33 | -36 | 0.54091 |
| 69 | -39 | -12 | 0.540942 |
| 21 | 57 | -21 | 0.543709 |
| 69 | -45 | -12 | 0.547405 |
| -33 | 30 | 42 | 0.550367 |
| -3 | 15 | 48 | 0.553212 |
| -48 | 12 | -39 | 0.557507 |
| -21 | 15 | 57 | 0.566086 |
| 15 | 63 | -21 | 0.567477 |
| 72 | -39 | -9 | 0.572548 |
| -33 | 15 | 6 | 0.57329 |
| 21 | -69 | -39 | 0.577544 |
| 60 | -57 | -12 | 0.577651 |
| 60 | -57 | 9 | 0.578076 |
| 63 | -57 | 9 | 0.582271 |
| 24 | -6 | -48 | 0.590176 |
| -66 | -39 | -3 | 0.590688 |
| 60 | -57 | 12 | 0.591123 |
| 6 | 39 | 36 | 0.592334 |
| -42 | -6 | -51 | 0.593322 |
| 60 | -63 | 12 | 0.594799 |
| 9 | 42 | 36 | 0.606465 |
| -30 | 21 | 6 | 0.60692 |
| -42 | -48 | -51 | 0.612094 |
| -69 | -36 | -6 | 0.612932 |
| -30 | 33 | 42 | 0.615176 |
| 72 | -39 | -12 | 0.615688 |
| 60 | -66 | 9 | 0.61609 |
| -30 | -3 | -33 | 0.61635 |
| -27 | -36 | -54 | 0.616704 |
| 12 | 45 | 42 | 0.617717 |
| 60 | -63 | -12 | 0.618272 |
| 69 | -36 | -9 | 0.619819 |
| -60 | 3 | 24 | 0.62044 |
| -60 | -36 | -3 | 0.623438 |
| -18 | 15 | 54 | 0.625976 |
| -15 | -18 | 78 | 0.626354 |
| -3 | 54 | -12 | 0.630902 |
| 66 | -39 | -12 | 0.633898 |
| -69 | -36 | -3 | 0.637434 |
| 60 | -60 | -12 | 0.641884 |
| 9 | 39 | 36 | 0.642289 |
| -6 | 72 | 0 | 0.646303 |
| -54 | 3 | -33 | 0.649999 |
| -39 | 0 | -30 | 0.651919 |
| 39 | -3 | -30 | 0.652968 |
| -24 | 45 | -21 | 0.653024 |
| -66 | -39 | -6 | 0.65675 |
| -36 | 18 | -45 | 0.658027 |
| 24 | 3 | 69 | 0.658948 |
| 6 | 42 | 39 | 0.663578 |
| -9 | -9 | 69 | 0.667546 |
| -33 | 30 | 39 | 0.674435 |
| 63 | -42 | -18 | 0.675381 |
| -30 | 12 | 60 | 0.676646 |
| 27 | 3 | 69 | 0.678146 |
| -57 | -9 | 48 | 0.682494 |
| -18 | -15 | 75 | 0.6832 |
| 66 | -42 | -9 | 0.683347 |
| -57 | 6 | -33 | 0.683468 |
| -15 | -15 | 78 | 0.684247 |
| -15 | 72 | 3 | 0.684308 |
| -66 | -36 | 33 | 0.686052 |
| -18 | 63 | 12 | 0.691119 |
| 0 | 12 | 45 | 0.699883 |
| 27 | 3 | 72 | 0.702428 |
| -24 | 0 | 45 | 0.703221 |
| -33 | -30 | -36 | 0.704323 |
| -36 | 45 | 9 | 0.70499 |
| 57 | -63 | 9 | 0.7063 |
| -33 | -36 | -33 | 0.706739 |
| -33 | 15 | 60 | 0.710866 |
| 21 | -69 | -36 | 0.714145 |
| -9 | 72 | 0 | 0.720993 |
| 6 | 42 | 33 | 0.723104 |
| 39 | 18 | -45 | 0.723354 |
| -33 | 30 | -9 | 0.724241 |
| -27 | 27 | 3 | 0.729689 |
| -18 | 72 | 3 | 0.731331 |
| 60 | -30 | 51 | 0.733229 |
| -12 | 42 | 30 | 0.737763 |
| 72 | -36 | -9 | 0.737902 |
| 12 | 42 | 18 | 0.739733 |
| 54 | 12 | -39 | 0.740839 |
| -36 | -57 | 18 | 0.741223 |
| -66 | -33 | 33 | 0.75166 |
| 54 | -63 | -12 | 0.753389 |
| -39 | -3 | -33 | 0.756804 |
| 60 | -66 | -12 | 0.7576 |
| -54 | 6 | -33 | 0.761181 |
| 12 | 30 | 39 | 0.761427 |
| -12 | 9 | 39 | 0.764783 |
| 0 | 24 | 57 | 0.765077 |
| 39 | -6 | -30 | 0.769011 |
| -30 | 15 | 57 | 0.77003 |
| -42 | -3 | -33 | 0.770841 |
| 63 | -45 | -18 | 0.773173 |
| -6 | -9 | 72 | 0.776389 |
| 51 | 12 | -39 | 0.77639 |
| -36 | 15 | -45 | 0.777246 |
| -39 | 30 | 42 | 0.77856 |
| -30 | -36 | -33 | 0.778755 |
| 18 | 57 | -21 | 0.782986 |
| -24 | -9 | -9 | 0.783473 |
| 33 | 30 | 3 | 0.784745 |
| -33 | 12 | 60 | 0.786003 |
| 24 | 9 | 60 | 0.793801 |
| 3 | 21 | 57 | 0.799703 |
| -18 | 39 | 48 | 0.801796 |
| -6 | -99 | 24 | 0.807161 |
| -30 | 45 | 36 | 0.808143 |
| 69 | -51 | -15 | 0.814226 |
| 63 | -30 | 51 | 0.82043 |
| 63 | -33 | 51 | 0.823973 |
| -69 | -45 | -12 | 0.825501 |
| -6 | 66 | 27 | 0.828494 |
| -18 | 18 | 57 | 0.829344 |
| 18 | 57 | -18 | 0.831398 |
| -42 | -45 | -51 | 0.831518 |
| -30 | 12 | 57 | 0.833617 |
| 0 | 15 | 42 | 0.835471 |
| 6 | 27 | 54 | 0.843359 |
| -33 | 30 | 45 | 0.845258 |
| -60 | -42 | -3 | 0.849306 |
| -21 | 39 | 48 | 0.851307 |
| -3 | -87 | -39 | 0.851382 |
| 69 | -42 | -12 | 0.85381 |
| -45 | -15 | -39 | 0.854913 |
| -33 | -27 | 21 | 0.854925 |
| -12 | -9 | 69 | 0.855678 |
| -9 | -84 | -39 | 0.858785 |
| 63 | -60 | 9 | 0.859979 |
| 9 | 42 | 18 | 0.861342 |
| -24 | -9 | -6 | 0.868528 |
| 9 | 39 | 33 | 0.872154 |
| -33 | 15 | 57 | 0.873147 |
| 15 | 63 | -18 | 0.875779 |
| 9 | 69 | -12 | 0.875938 |
| 24 | -69 | -39 | 0.876613 |
| 9 | -27 | 39 | 0.879443 |
| -6 | 72 | -3 | 0.880749 |
| -27 | -9 | -6 | 0.882712 |
| -66 | -33 | 30 | 0.884034 |
| 27 | -57 | -42 | 0.887894 |
| -30 | 30 | 45 | 0.88869 |
| 12 | 69 | -12 | 0.888866 |
| -21 | 36 | 48 | 0.893989 |
| -15 | 57 | 6 | 0.895012 |
| -30 | 9 | 60 | 0.89562 |
| 3 | -33 | 48 | 0.895641 |
| -63 | -15 | -30 | 0.89622 |
| 33 | -87 | 21 | 0.89881 |
| -15 | 63 | 6 | 0.900596 |
| -39 | 48 | 12 | 0.901081 |
| -21 | 42 | 51 | 0.903319 |
| -21 | 48 | 6 | 0.905667 |
| 30 | 42 | -21 | 0.908546 |
| 69 | -45 | -15 | 0.909419 |
| -33 | 15 | 3 | 0.913497 |
| -42 | -6 | -27 | 0.915717 |
| 18 | 60 | -21 | 0.916876 |
| -27 | 45 | -18 | 0.917597 |
| 15 | 66 | -21 | 0.918258 |
| 15 | 60 | -15 | 0.918605 |
| 33 | -12 | -27 | 0.919392 |
| 15 | -84 | -42 | 0.919724 |
| 54 | 3 | 48 | 0.921219 |
| -30 | 24 | 6 | 0.921256 |
| -6 | -81 | -36 | 0.925094 |
| 72 | -36 | -12 | 0.931713 |
| -63 | -33 | -6 | 0.931889 |
| -9 | 69 | 0 | 0.932648 |
| 66 | -42 | -12 | 0.934769 |
| 12 | -27 | 42 | 0.935188 |
| -54 | -75 | -9 | 0.935354 |
| 60 | -66 | 12 | 0.937751 |
| 63 | -57 | 12 | 0.940239 |
| -30 | -3 | -30 | 0.942604 |
| 57 | -66 | -12 | 0.942821 |
| -39 | -6 | -27 | 0.944911 |
| -30 | 15 | 60 | 0.949007 |
| 45 | -42 | 66 | 0.950152 |
| -63 | -33 | -3 | 0.951127 |
| -30 | 42 | -21 | 0.953997 |
| -6 | -84 | -36 | 0.954695 |
| -60 | -39 | -6 | 0.95482 |
| 12 | 18 | 42 | 0.955794 |
| -33 | 36 | 3 | 0.95924 |
| 54 | -78 | 3 | 0.959262 |
| -45 | -15 | -36 | 0.962341 |
| 54 | -60 | -12 | 0.962569 |
| 12 | 27 | 36 | 0.964456 |
| 66 | -48 | -15 | 0.965531 |
| 60 | -36 | 54 | 0.966559 |
| -3 | 72 | 0 | 0.966828 |
| -3 | -99 | 24 | 0.968278 |
| 15 | 60 | -21 | 0.969124 |
| 3 | 27 | 57 | 0.970684 |
| 57 | -63 | -9 | 0.971711 |
| 54 | 6 | 45 | 0.973867 |
| -60 | -36 | -6 | 0.975376 |
| -18 | -18 | 75 | 0.975439 |
| -6 | -90 | -42 | 0.979544 |
| 12 | -3 | 57 | 0.980329 |
| -69 | -42 | -15 | 0.980745 |
| -18 | 42 | 42 | 0.982244 |
| 66 | -45 | -15 | 0.982936 |
| -57 | -6 | 48 | 0.983421 |
| 0 | 63 | -21 | 0.985244 |
| -36 | 42 | 9 | 0.985432 |
| -15 | 42 | 33 | 0.986915 |
| -60 | -12 | 48 | 0.986983 |
| 12 | -84 | -42 | 0.987156 |
| 66 | -36 | -9 | 0.98808 |
| -18 | 60 | 12 | 0.989286 |
| 63 | -57 | -12 | 0.99094 |
| 27 | 0 | 69 | 0.991332 |
| 18 | 72 | 9 | 0.992666 |
| -21 | 36 | 42 | 0.99507 |
| 57 | -57 | 9 | 0.995344 |
| -12 | 6 | 39 | 0.995907 |
| -48 | -18 | -39 | 0.996218 |
| -9 | -84 | -42 | 0.996911 |
| 9 | 24 | -15 | 0.99804 |
| -27 | 36 | 39 | 0.998161 |
| -30 | 0 | -30 | 0.998369 |
| -48 | 12 | -36 | 0.998815 |
| 51 | 12 | -42 | 0.999129 |
| 0 | 15 | 51 | 0.999701 |
| 9 | 12 | 45 | 0.999708 |
| 69 | -45 | -9 | 0.999817 |

Notes: GCL, ganglion cell layer; IPL, inner plexiform layer.

**Table S4. There was no significant correlation between the cognition-RNFL- FALFF in HC and SCD patients.**

| X (mm) | Y (mm) | Z (mm) | P_FWE_ |
| --- | --- | --- | --- |
| -21 | -12 | 75 | 0.052699 |
| -6 | -87 | -39 | 0.057713 |
| -12 | -90 | -42 | 0.073957 |
| -9 | -87 | -39 | 0.075682 |
| -39 | -54 | 63 | 0.075949 |
| -21 | -15 | 75 | 0.077872 |
| -24 | -12 | 75 | 0.109583 |
| -30 | -15 | 72 | 0.113225 |
| -27 | -12 | 72 | 0.131282 |
| -18 | -18 | 75 | 0.140071 |
| -18 | -15 | 75 | 0.144325 |
| -60 | -12 | 48 | 0.146746 |
| -51 | -9 | 54 | 0.158353 |
| -6 | -84 | -42 | 0.162563 |
| -9 | -93 | -42 | 0.186275 |
| -57 | -12 | 48 | 0.187307 |
| -12 | -87 | -42 | 0.187999 |
| -9 | -84 | -42 | 0.189898 |
| 42 | -54 | -42 | 0.191798 |
| 51 | 12 | -39 | 0.193327 |
| 12 | -42 | 6 | 0.194347 |
| -12 | -24 | 9 | 0.196037 |
| -12 | -93 | -42 | 0.196708 |
| -60 | -15 | 45 | 0.197551 |
| 12 | -45 | 6 | 0.19784 |
| -15 | -24 | 9 | 0.199986 |
| -15 | -21 | 12 | 0.200609 |
| -60 | -15 | 48 | 0.205388 |
| -57 | -15 | 45 | 0.220762 |
| -57 | -15 | 48 | 0.225946 |
| -27 | -15 | 72 | 0.232184 |
| -12 | -24 | 6 | 0.242949 |
| -21 | -12 | 72 | 0.245173 |
| -60 | -6 | -39 | 0.253328 |
| -27 | -9 | 72 | 0.267944 |
| -12 | -21 | 15 | 0.276973 |
| -30 | -42 | -6 | 0.278774 |
| -24 | -12 | 72 | 0.279259 |
| -42 | -54 | 60 | 0.280416 |
| 42 | -51 | -42 | 0.292277 |
| -24 | -15 | 75 | 0.294101 |
| -30 | -42 | -9 | 0.302472 |
| -39 | 0 | -27 | 0.304626 |
| -30 | -12 | 72 | 0.311932 |
| -54 | -9 | 54 | 0.318411 |
| -57 | -12 | 45 | 0.323302 |
| -33 | -84 | -42 | 0.327002 |
| -15 | -24 | 12 | 0.330294 |
| -9 | -78 | -45 | 0.331693 |
| 12 | -24 | 15 | 0.335535 |
| -15 | -21 | 15 | 0.336435 |
| -15 | -87 | -42 | 0.342272 |
| -30 | -42 | -12 | 0.342333 |
| -18 | -12 | 75 | 0.352486 |
| -51 | -9 | 51 | 0.356709 |
| -12 | -87 | -39 | 0.360599 |
| -27 | -42 | -12 | 0.364878 |
| -39 | -54 | 60 | 0.367146 |
| -18 | -45 | 78 | 0.372961 |
| -33 | -42 | -6 | 0.374658 |
| 42 | -81 | -48 | 0.381888 |
| 42 | -54 | -39 | 0.387763 |
| -6 | -81 | -45 | 0.390935 |
| -15 | -90 | -42 | 0.395073 |
| -18 | -21 | 15 | 0.402179 |
| -12 | -21 | 12 | 0.405312 |
| -27 | -45 | -12 | 0.405746 |
| -54 | -6 | 54 | 0.408909 |
| -9 | -90 | -39 | 0.412476 |
| -33 | -45 | -6 | 0.416625 |
| -21 | -18 | 75 | 0.418499 |
| -48 | -12 | 57 | 0.433275 |
| 12 | -21 | 3 | 0.43964 |
| -33 | -69 | -33 | 0.440639 |
| 24 | -48 | -27 | 0.440927 |
| -15 | -27 | 9 | 0.444071 |
| 27 | -42 | 75 | 0.455518 |
| -30 | -45 | -9 | 0.455525 |
| 15 | -87 | -42 | 0.458136 |
| 0 | 63 | -21 | 0.466527 |
| -36 | -54 | 63 | 0.471968 |
| -39 | -12 | -18 | 0.485029 |
| -21 | -48 | 75 | 0.488346 |
| 51 | 15 | -39 | 0.490757 |
| 51 | -69 | -51 | 0.495952 |
| -9 | -72 | -48 | 0.500672 |
| -54 | -6 | 51 | 0.508232 |
| 60 | -6 | -6 | 0.512151 |
| 30 | -30 | -27 | 0.512479 |
| -57 | -9 | 48 | 0.512581 |
| 54 | 12 | -39 | 0.512614 |
| 9 | -18 | 3 | 0.516282 |
| -3 | -87 | -42 | 0.517787 |
| -12 | -18 | 12 | 0.522754 |
| -12 | -78 | -45 | 0.524139 |
| -24 | -84 | -39 | 0.530353 |
| 15 | -84 | -45 | 0.534459 |
| -36 | -84 | -42 | 0.538836 |
| 9 | -21 | 0 | 0.543253 |
| -15 | -15 | 75 | 0.543537 |
| -15 | -18 | 75 | 0.546007 |
| -12 | -24 | 12 | 0.546298 |
| 30 | -87 | -45 | 0.550016 |
| -15 | -18 | 12 | 0.557211 |
| -60 | -12 | 45 | 0.557466 |
| 51 | 12 | -42 | 0.560245 |
| -54 | -9 | 51 | 0.570541 |
| 30 | 66 | 0 | 0.571165 |
| -33 | -15 | 72 | 0.573686 |
| -15 | -51 | -21 | 0.574751 |
| -6 | -84 | -39 | 0.580091 |
| -30 | -9 | 72 | 0.585092 |
| -27 | -42 | -9 | 0.587375 |
| -33 | -45 | -9 | 0.590627 |
| -48 | -9 | 57 | 0.600542 |
| -39 | -3 | -27 | 0.602657 |
| 12 | -21 | -9 | 0.605508 |
| -9 | -21 | -6 | 0.608642 |
| -15 | -15 | 78 | 0.611475 |
| 12 | -39 | 6 | 0.614317 |
| -9 | -21 | -9 | 0.614498 |
| 6 | -87 | -42 | 0.619041 |
| 42 | -48 | -42 | 0.62379 |
| -21 | -15 | 72 | 0.624294 |
| -6 | -21 | -9 | 0.624987 |
| 15 | -87 | -45 | 0.6286 |
| 21 | -48 | -24 | 0.63109 |
| -15 | -21 | 9 | 0.631237 |
| 12 | -87 | -42 | 0.63152 |
| 9 | -45 | 6 | 0.631607 |
| -12 | -21 | 9 | 0.633734 |
| -30 | -84 | -42 | 0.639818 |
| -39 | 57 | 15 | 0.641519 |
| -15 | -24 | 6 | 0.643413 |
| 12 | -21 | -6 | 0.645154 |
| 9 | -21 | 3 | 0.648447 |
| -33 | -87 | -42 | 0.648963 |
| -9 | -75 | -45 | 0.650412 |
| -9 | -75 | -48 | 0.653594 |
| -24 | -9 | 72 | 0.654333 |
| -3 | -81 | -45 | 0.659801 |
| -6 | -90 | -39 | 0.659815 |
| 33 | -87 | -45 | 0.661044 |
| -27 | -15 | -3 | 0.669051 |
| -27 | -81 | -42 | 0.669117 |
| -21 | -39 | -3 | 0.671297 |
| -27 | -42 | -15 | 0.672233 |
| -15 | 66 | -12 | 0.6825 |
| -18 | -15 | 72 | 0.687701 |
| 15 | -21 | 9 | 0.698389 |
| -18 | -27 | 9 | 0.701189 |
| -60 | -18 | 45 | 0.702988 |
| 15 | -24 | 9 | 0.704375 |
| -9 | -18 | -6 | 0.70522 |
| 0 | 60 | -21 | 0.711798 |
| -18 | -30 | 9 | 0.714091 |
| 15 | -27 | 12 | 0.716222 |
| -18 | -51 | -21 | 0.717049 |
| -60 | -9 | -39 | 0.717482 |
| 15 | -24 | 12 | 0.718162 |
| 15 | -24 | 15 | 0.722743 |
| 15 | -81 | -45 | 0.724951 |
| -12 | -90 | -39 | 0.726406 |
| 9 | -81 | -45 | 0.727885 |
| 0 | 15 | 42 | 0.735789 |
| -36 | -57 | 63 | 0.737699 |
| 15 | -84 | -42 | 0.738511 |
| 18 | -84 | -45 | 0.739503 |
| 12 | -42 | 9 | 0.739934 |
| -12 | -18 | 15 | 0.740917 |
| -48 | -9 | 60 | 0.745596 |
| 45 | -63 | -51 | 0.746477 |
| 18 | -21 | 9 | 0.749464 |
| 12 | -3 | -6 | 0.749901 |
| -6 | -78 | -45 | 0.749996 |
| -36 | -87 | -42 | 0.752231 |
| -12 | -24 | 15 | 0.752813 |
| -9 | -69 | -48 | 0.755397 |
| 60 | -27 | 54 | 0.75927 |
| 6 | -87 | -45 | 0.760131 |
| -12 | -69 | -48 | 0.761292 |
| 15 | -6 | -6 | 0.764045 |
| -24 | -87 | -39 | 0.769794 |
| 48 | -63 | -51 | 0.776655 |
| -57 | -6 | -39 | 0.778383 |
| 6 | -84 | -45 | 0.778574 |
| -12 | -21 | 6 | 0.780938 |
| 39 | -84 | -45 | 0.783678 |
| -27 | -84 | -39 | 0.784085 |
| -18 | -39 | -3 | 0.78687 |
| -30 | -45 | -12 | 0.787633 |
| -6 | -72 | -48 | 0.787725 |
| -21 | 48 | 45 | 0.788686 |
| -42 | -54 | 57 | 0.788761 |
| -18 | -78 | 54 | 0.791331 |
| 3 | -87 | -42 | 0.797255 |
| 12 | -18 | 3 | 0.802018 |
| 15 | -6 | -9 | 0.802873 |
| -36 | -72 | -45 | 0.803199 |
| 21 | -51 | -24 | 0.807737 |
| -6 | 66 | 24 | 0.808918 |
| -51 | -12 | 54 | 0.812446 |
| -18 | -21 | 12 | 0.813073 |
| -12 | -18 | -6 | 0.81457 |
| -9 | -9 | 72 | 0.814669 |
| 0 | 12 | 48 | 0.818362 |
| -27 | -84 | -42 | 0.81909 |
| 12 | -24 | 12 | 0.820253 |
| -30 | -87 | -42 | 0.821457 |
| -30 | -81 | -42 | 0.822237 |
| -9 | -21 | 0 | 0.825746 |
| -54 | -75 | -12 | 0.832469 |
| -9 | -81 | -45 | 0.832602 |
| 30 | -42 | 72 | 0.835694 |
| 9 | -87 | -42 | 0.835783 |
| -6 | -21 | -6 | 0.836555 |
| -6 | -18 | -6 | 0.837213 |
| 12 | -84 | -45 | 0.846859 |
| -48 | -12 | 60 | 0.847161 |
| -36 | -69 | -33 | 0.847957 |
| -9 | -21 | 3 | 0.848365 |
| 12 | -45 | 9 | 0.849133 |
| -33 | -69 | -48 | 0.849625 |
| -30 | -45 | -6 | 0.851483 |
| 51 | -66 | -48 | 0.85358 |
| -15 | -24 | 15 | 0.855709 |
| -33 | -57 | -6 | 0.856677 |
| -6 | -84 | -45 | 0.857178 |
| -33 | -54 | -9 | 0.857415 |
| 12 | -87 | -45 | 0.859274 |
| -54 | -12 | 48 | 0.860289 |
| -24 | -81 | 51 | 0.861262 |
| 12 | -81 | -45 | 0.863146 |
| 51 | -69 | -48 | 0.863413 |
| 6 | -90 | -42 | 0.863558 |
| 0 | 12 | 45 | 0.865094 |
| -51 | -36 | -21 | 0.867999 |
| -33 | -54 | -12 | 0.868877 |
| 12 | -27 | 12 | 0.874125 |
| 45 | -54 | -42 | 0.878029 |
| -33 | 39 | 3 | 0.881632 |
| 9 | -84 | -45 | 0.885195 |
| 60 | -30 | 54 | 0.888288 |
| -9 | -24 | 6 | 0.889431 |
| -36 | -75 | -45 | 0.889756 |
| -60 | -15 | 42 | 0.89188 |
| -24 | -48 | -15 | 0.892709 |
| -15 | -93 | -45 | 0.893991 |
| 12 | -18 | -6 | 0.902718 |
| 48 | -60 | -51 | 0.903262 |
| 51 | -57 | -48 | 0.904102 |
| 60 | -24 | 54 | 0.904765 |
| -12 | -90 | -45 | 0.905894 |
| 15 | -18 | -6 | 0.907245 |
| -36 | -57 | -6 | 0.913134 |
| 39 | -81 | -48 | 0.915204 |
| -30 | -36 | -51 | 0.915562 |
| -12 | -27 | 9 | 0.918264 |
| -18 | -81 | 54 | 0.921052 |
| 12 | -90 | -45 | 0.923246 |
| -27 | -45 | -15 | 0.929287 |
| 6 | 30 | -21 | 0.933857 |
| -18 | -36 | 0 | 0.935284 |
| 6 | -84 | -42 | 0.939309 |
| -39 | 0 | -30 | 0.941704 |
| -9 | -84 | -39 | 0.942003 |
| -18 | -87 | -42 | 0.942246 |
| 51 | -78 | 15 | 0.942735 |
| -27 | -12 | -6 | 0.943356 |
| 12 | -21 | 6 | 0.949609 |
| -15 | -21 | 6 | 0.94998 |
| -9 | -24 | 3 | 0.950223 |
| -21 | -42 | -3 | 0.951006 |
| 30 | 33 | -9 | 0.951281 |
| 42 | -81 | -45 | 0.951635 |
| 51 | -78 | 18 | 0.951997 |
| -18 | -12 | 72 | 0.95294 |
| -18 | -18 | 72 | 0.954354 |
| -9 | -21 | 15 | 0.955969 |
| -15 | -18 | 78 | 0.956804 |
| -27 | -48 | -15 | 0.95738 |
| -12 | -84 | -42 | 0.958147 |
| -15 | -30 | 9 | 0.958866 |
| 33 | 33 | 3 | 0.959278 |
| 12 | -27 | 15 | 0.961104 |
| 48 | -69 | -51 | 0.961516 |
| -15 | -90 | -45 | 0.962695 |
| 57 | 3 | 45 | 0.962769 |
| -21 | -81 | 54 | 0.96303 |
| -21 | -9 | 75 | 0.96331 |
| 60 | -60 | 9 | 0.965033 |
| -21 | -36 | 0 | 0.965659 |
| 3 | 12 | 48 | 0.968057 |
| 45 | -51 | -42 | 0.968362 |
| -24 | -51 | -30 | 0.968514 |
| -51 | -6 | 51 | 0.969522 |
| 12 | -24 | 3 | 0.971217 |
| -3 | -96 | 27 | 0.972178 |
| -18 | -48 | 78 | 0.972981 |
| -27 | -45 | -9 | 0.974369 |
| -39 | -3 | -30 | 0.975264 |
| 30 | 33 | -3 | 0.975995 |
| -36 | -6 | 15 | 0.97694 |
| 51 | -75 | 18 | 0.977065 |
| 12 | -84 | -42 | 0.980707 |
| -6 | -9 | 72 | 0.980716 |
| 18 | -18 | -6 | 0.981193 |
| 42 | -57 | -39 | 0.982324 |
| 48 | -66 | -48 | 0.984825 |
| -54 | -75 | -9 | 0.985943 |
| 21 | -48 | -27 | 0.986734 |
| -54 | -9 | 48 | 0.98798 |
| 12 | -24 | 9 | 0.989832 |
| -36 | 57 | 15 | 0.990059 |
| -3 | -99 | 24 | 0.991063 |
| -15 | -93 | -42 | 0.991648 |
| -15 | -27 | 12 | 0.992668 |
| 30 | 33 | -6 | 0.993547 |
| -6 | -75 | -48 | 0.994477 |
| 39 | -30 | -30 | 0.994592 |
| -21 | -78 | 54 | 0.994903 |
| 57 | -60 | 9 | 0.995243 |
| -12 | -93 | -39 | 0.995316 |
| -6 | 66 | 21 | 0.995386 |
| 18 | -87 | -45 | 0.996287 |
| 42 | -78 | -48 | 0.997225 |
| -36 | -69 | -48 | 0.997826 |
| 48 | -66 | -51 | 0.998012 |
| 21 | -54 | -21 | 0.998324 |
| -15 | 6 | 24 | 0.999254 |
| -27 | -51 | -30 | 0.999586 |
|  |  |  |  |

Notes: RNFL, retinal nerve fiber layer.

**Table S5. There was no significant correlation between the cognition-RBVC - FALFF in HC and SCD patients.**

| X (mm) | Y (mm) | Z (mm) | P_FWE_ |
| --- | --- | --- | --- |
| -39 | 27 | -21 | 0.051303 |
| -42 | 27 | -18 | 0.066759 |
| -45 | 30 | -21 | 0.068946 |
| -42 | 30 | -21 | 0.085427 |
| -39 | 27 | -18 | 0.091063 |
| -24 | 3 | -39 | 0.101318 |
| -45 | 27 | -18 | 0.103178 |
| -45 | -69 | -42 | 0.111657 |
| -30 | -30 | -15 | 0.115055 |
| -18 | -81 | -42 | 0.119061 |
| -48 | -69 | -42 | 0.127475 |
| -15 | -81 | -42 | 0.135348 |
| -33 | -69 | -30 | 0.141774 |
| -27 | 39 | 42 | 0.144582 |
| -30 | -30 | -18 | 0.147553 |
| 21 | -6 | -36 | 0.158183 |
| -6 | -102 | 18 | 0.158942 |
| -33 | -66 | -27 | 0.161942 |
| -24 | 0 | -39 | 0.169252 |
| 18 | 15 | -21 | 0.171815 |
| -24 | 3 | -42 | 0.18491 |
| -21 | -6 | -30 | 0.18827 |
| -36 | 9 | -45 | 0.192774 |
| -45 | -72 | -42 | 0.195916 |
| -18 | -9 | -30 | 0.206104 |
| -42 | 30 | -18 | 0.210724 |
| -33 | -72 | -30 | 0.216661 |
| -21 | -9 | -30 | 0.22186 |
| -15 | -81 | 51 | 0.229771 |
| -36 | -72 | -30 | 0.229786 |
| -33 | -63 | -30 | 0.230412 |
| -36 | 9 | -42 | 0.238405 |
| -24 | -6 | -6 | 0.241566 |
| -30 | -63 | -30 | 0.245421 |
| -15 | -81 | 48 | 0.246011 |
| -12 | -81 | 51 | 0.25931 |
| -21 | -6 | -33 | 0.26315 |
| -39 | 24 | -21 | 0.264505 |
| 36 | 42 | -15 | 0.26599 |
| -54 | -18 | -39 | 0.270267 |
| -33 | -33 | -15 | 0.281384 |
| -27 | 3 | -39 | 0.282113 |
| -60 | 6 | -27 | 0.284993 |
| -45 | 30 | -18 | 0.288536 |
| -33 | -66 | -30 | 0.290361 |
| -9 | -81 | 51 | 0.292058 |
| -15 | 72 | 3 | 0.306472 |
| 33 | -39 | -21 | 0.311986 |
| -30 | -33 | -15 | 0.315633 |
| 48 | 27 | -12 | 0.316351 |
| 12 | -75 | -18 | 0.321765 |
| -42 | -69 | -42 | 0.32548 |
| -6 | -99 | 18 | 0.329588 |
| -30 | -39 | -33 | 0.329859 |
| -33 | -30 | -15 | 0.334164 |
| -33 | -39 | -12 | 0.336781 |
| 27 | -12 | -18 | 0.343022 |
| 36 | 51 | -18 | 0.350092 |
| 21 | -18 | 12 | 0.356044 |
| -33 | -69 | -27 | 0.356776 |
| -36 | -72 | -33 | 0.357381 |
| -33 | -39 | -33 | 0.367067 |
| -27 | -36 | -12 | 0.368989 |
| -36 | -69 | -30 | 0.369111 |
| 33 | 51 | -18 | 0.37414 |
| -9 | -102 | 18 | 0.376474 |
| 42 | -48 | -45 | 0.381533 |
| 45 | -12 | -27 | 0.384097 |
| 54 | -12 | -30 | 0.388406 |
| -54 | 15 | -18 | 0.389435 |
| -21 | -51 | 72 | 0.392842 |
| -12 | -3 | 9 | 0.392921 |
| -36 | -63 | -30 | 0.403396 |
| 33 | -36 | -21 | 0.407955 |
| -30 | -60 | -30 | 0.414573 |
| -27 | 42 | 42 | 0.416735 |
| -36 | -39 | -15 | 0.419624 |
| -39 | 24 | -18 | 0.422567 |
| 18 | 18 | -24 | 0.427943 |
| 24 | -27 | 9 | 0.434089 |
| 51 | -12 | -30 | 0.43616 |
| 45 | -42 | -36 | 0.437321 |
| -6 | -84 | -39 | 0.437402 |
| 51 | 30 | -9 | 0.438277 |
| -42 | -69 | -45 | 0.441552 |
| 36 | 51 | -15 | 0.442486 |
| -9 | -102 | 15 | 0.462518 |
| 39 | 45 | -15 | 0.464333 |
| 48 | -12 | -27 | 0.467876 |
| -9 | -99 | 18 | 0.47401 |
| -57 | -18 | -39 | 0.477206 |
| -24 | 0 | -36 | 0.478569 |
| 45 | -9 | -27 | 0.480102 |
| -42 | -3 | -39 | 0.480303 |
| 21 | -6 | -33 | 0.481688 |
| -51 | -69 | -42 | 0.484788 |
| -15 | -84 | 48 | 0.490216 |
| 24 | -9 | -33 | 0.492336 |
| -6 | -87 | -39 | 0.494833 |
| 9 | -12 | 0 | 0.502377 |
| -39 | 0 | -39 | 0.515469 |
| 12 | 3 | -6 | 0.519191 |
| 48 | 12 | 48 | 0.519955 |
| 24 | -12 | -18 | 0.522618 |
| -36 | -27 | -27 | 0.525755 |
| -21 | -3 | -33 | 0.527104 |
| -39 | -3 | -39 | 0.529298 |
| -33 | -30 | -18 | 0.536808 |
| -57 | -18 | -24 | 0.538055 |
| 21 | -9 | -39 | 0.539391 |
| 9 | 24 | 6 | 0.540417 |
| 24 | -15 | -30 | 0.553034 |
| 18 | 18 | -21 | 0.554565 |
| 45 | 12 | 51 | 0.558033 |
| 60 | 6 | 39 | 0.558035 |
| 45 | -45 | -45 | 0.562018 |
| -36 | -66 | -30 | 0.563959 |
| 33 | 42 | -15 | 0.566027 |
| -15 | -54 | -15 | 0.566823 |
| 42 | -39 | -24 | 0.569488 |
| -33 | -39 | -15 | 0.571047 |
| -48 | -66 | -42 | 0.576513 |
| -12 | -81 | 48 | 0.579834 |
| -21 | -54 | 72 | 0.583145 |
| -24 | 6 | -39 | 0.584052 |
| 45 | 12 | 54 | 0.587194 |
| 51 | -12 | -33 | 0.598467 |
| 39 | -48 | -45 | 0.599436 |
| 36 | -36 | -21 | 0.60011 |
| -33 | -63 | -27 | 0.606516 |
| 6 | 21 | 6 | 0.609244 |
| -33 | -33 | -18 | 0.610167 |
| 24 | -9 | -36 | 0.611178 |
| -33 | 9 | -45 | 0.613662 |
| -24 | 6 | -42 | 0.614601 |
| -57 | -15 | -24 | 0.617441 |
| -24 | -3 | -36 | 0.618961 |
| -18 | -51 | 72 | 0.620731 |
| -15 | -60 | -21 | 0.620807 |
| -57 | -21 | -39 | 0.631124 |
| -54 | 12 | -18 | 0.631963 |
| -27 | -33 | -15 | 0.638006 |
| -45 | -42 | -18 | 0.640229 |
| -36 | 6 | -42 | 0.642112 |
| 48 | -12 | -30 | 0.644083 |
| 21 | -60 | -36 | 0.645872 |
| -30 | -66 | -30 | 0.6493 |
| 57 | 0 | 45 | 0.655246 |
| 48 | -12 | -33 | 0.657475 |
| 48 | 12 | 51 | 0.658162 |
| 48 | -12 | -24 | 0.658317 |
| -18 | -6 | -30 | 0.664579 |
| 36 | 54 | -18 | 0.669157 |
| -39 | -39 | -18 | 0.670847 |
| -30 | -63 | -27 | 0.675324 |
| 30 | 39 | -21 | 0.67566 |
| -39 | 30 | -18 | 0.676494 |
| -15 | 72 | 0 | 0.68231 |
| 57 | -12 | -30 | 0.693363 |
| 36 | 45 | -15 | 0.699042 |
| -27 | 42 | 39 | 0.699769 |
| -42 | 0 | -39 | 0.703817 |
| -30 | -30 | -21 | 0.705671 |
| -21 | -57 | 72 | 0.709133 |
| -33 | -99 | -12 | 0.712299 |
| 27 | -15 | -18 | 0.714664 |
| -60 | -18 | -39 | 0.714991 |
| -27 | -39 | -12 | 0.715914 |
| -18 | -81 | 51 | 0.722825 |
| -39 | -18 | -21 | 0.728196 |
| -42 | 18 | 54 | 0.728247 |
| -36 | -63 | -33 | 0.72982 |
| -33 | 33 | -12 | 0.731754 |
| 57 | -9 | -30 | 0.736847 |
| -24 | -36 | -12 | 0.736899 |
| -39 | 30 | -21 | 0.737293 |
| 33 | 42 | -21 | 0.740419 |
| -18 | -51 | -18 | 0.740823 |
| -6 | -102 | 15 | 0.742607 |
| -45 | -66 | -42 | 0.746255 |
| 48 | 24 | -12 | 0.749514 |
| 42 | -45 | -45 | 0.750056 |
| 30 | -36 | -24 | 0.752079 |
| -27 | -84 | 36 | 0.753474 |
| -42 | 30 | -15 | 0.753981 |
| 48 | -42 | -39 | 0.75589 |
| 36 | -39 | -21 | 0.756871 |
| -33 | 33 | -15 | 0.757939 |
| -33 | -39 | -30 | 0.758024 |
| -15 | -27 | 18 | 0.759454 |
| -39 | -69 | -48 | 0.76156 |
| -33 | 6 | -42 | 0.761718 |
| -30 | -99 | -12 | 0.763356 |
| 15 | 3 | -9 | 0.763852 |
| -42 | -39 | -18 | 0.768202 |
| -33 | -30 | -24 | 0.771893 |
| -39 | -18 | -24 | 0.77699 |
| -36 | -27 | -24 | 0.78005 |
| 54 | 21 | -9 | 0.780134 |
| -21 | -81 | 51 | 0.781021 |
| -24 | -87 | 33 | 0.78467 |
| -33 | -6 | -18 | 0.790766 |
| 21 | -6 | -39 | 0.79349 |
| -60 | -18 | -24 | 0.799455 |
| -36 | -36 | -18 | 0.79946 |
| 36 | 42 | -18 | 0.800933 |
| 36 | 48 | -15 | 0.801025 |
| -60 | -18 | -36 | 0.801583 |
| -57 | -18 | -36 | 0.802387 |
| 63 | 6 | 39 | 0.802421 |
| -42 | -42 | -18 | 0.805238 |
| 48 | 9 | 51 | 0.807916 |
| 15 | 6 | -9 | 0.808392 |
| 30 | 36 | -21 | 0.808747 |
| -24 | 0 | -42 | 0.808764 |
| -24 | 42 | 42 | 0.814084 |
| -27 | -30 | -18 | 0.818515 |
| -57 | 12 | -18 | 0.81861 |
| -15 | -63 | -21 | 0.819747 |
| -18 | -84 | 48 | 0.822678 |
| -57 | -15 | -27 | 0.822816 |
| 24 | -15 | -24 | 0.824903 |
| 33 | 51 | -21 | 0.830506 |
| 51 | -15 | -33 | 0.837002 |
| -24 | -3 | 0 | 0.839746 |
| 0 | -102 | -6 | 0.846879 |
| 21 | -9 | -36 | 0.849644 |
| 39 | -39 | -21 | 0.850023 |
| 36 | 51 | -21 | 0.850845 |
| -15 | 69 | 3 | 0.852832 |
| 27 | -3 | -12 | 0.861892 |
| 51 | 30 | -12 | 0.862932 |
| 48 | -78 | 30 | 0.86425 |
| 24 | -12 | -15 | 0.864577 |
| -54 | -21 | -39 | 0.865038 |
| -30 | -84 | 36 | 0.865381 |
| 24 | -6 | -36 | 0.866305 |
| -12 | 72 | 0 | 0.866526 |
| 30 | 9 | 0 | 0.873679 |
| -48 | -72 | -42 | 0.879341 |
| 21 | -63 | -36 | 0.88067 |
| -21 | -99 | 15 | 0.881515 |
| -27 | -30 | -15 | 0.888595 |
| -33 | 9 | -42 | 0.889608 |
| 33 | 42 | -18 | 0.892744 |
| -42 | 24 | -18 | 0.892839 |
| 30 | -39 | -21 | 0.894904 |
| -42 | -24 | -30 | 0.900219 |
| -30 | -27 | -18 | 0.901225 |
| -42 | -69 | -39 | 0.902709 |
| -39 | -54 | -6 | 0.905895 |
| 30 | 51 | -18 | 0.906037 |
| -36 | -66 | -27 | 0.90611 |
| 24 | -12 | -33 | 0.906112 |
| -36 | -9 | -21 | 0.915728 |
| 42 | -21 | -12 | 0.916242 |
| 24 | -9 | -15 | 0.916565 |
| -27 | 39 | 39 | 0.916922 |
| 33 | 39 | -21 | 0.917491 |
| 24 | -9 | -12 | 0.917659 |
| -27 | -84 | 33 | 0.918466 |
| -36 | -60 | -33 | 0.923136 |
| -33 | -72 | -33 | 0.924468 |
| 45 | -42 | -39 | 0.926419 |
| -21 | -3 | -36 | 0.927071 |
| 24 | -18 | -24 | 0.929498 |
| 12 | 6 | -6 | 0.932582 |
| 48 | -12 | -36 | 0.933504 |
| -33 | -66 | -24 | 0.93373 |
| -36 | -69 | -33 | 0.934373 |
| -42 | -66 | -42 | 0.936362 |
| -30 | -39 | -12 | 0.938259 |
| 27 | -3 | -15 | 0.938332 |
| -15 | -51 | -15 | 0.939405 |
| 36 | 39 | -15 | 0.939489 |
| 48 | -24 | -27 | 0.940319 |
| -33 | -60 | -30 | 0.942735 |
| 54 | 24 | -9 | 0.943678 |
| -39 | -36 | -21 | 0.945529 |
| -45 | -72 | -45 | 0.945727 |
| -39 | -66 | -48 | 0.948086 |
| 45 | -27 | -27 | 0.949011 |
| 33 | -36 | -33 | 0.949901 |
| -57 | -12 | -27 | 0.952873 |
| -60 | -15 | -24 | 0.957868 |
| -18 | -48 | 75 | 0.959549 |
| 21 | -9 | -33 | 0.96185 |
| -57 | 6 | -27 | 0.962046 |
| -24 | -39 | -12 | 0.963081 |
| -30 | -6 | -15 | 0.963325 |
| 30 | 45 | 42 | 0.965221 |
| -45 | -69 | -45 | 0.96563 |
| -39 | -69 | -45 | 0.967124 |
| -42 | -27 | -30 | 0.968901 |
| 24 | -12 | 3 | 0.97078 |
| -24 | -3 | -33 | 0.971259 |
| -27 | 0 | -39 | 0.972722 |
| 48 | 3 | -42 | 0.972814 |
| -36 | 12 | -45 | 0.972864 |
| 9 | 24 | 3 | 0.97436 |
| -30 | -36 | -12 | 0.974403 |
| 42 | 27 | 42 | 0.974618 |
| 39 | -36 | -21 | 0.975033 |
| 48 | -15 | -27 | 0.97691 |
| -36 | -6 | -21 | 0.978621 |
| -30 | -39 | -30 | 0.979699 |
| -33 | -42 | -12 | 0.980997 |
| 51 | 24 | -9 | 0.981899 |
| 15 | -69 | -39 | 0.982835 |
| 48 | 30 | -12 | 0.985077 |
| -27 | 6 | -39 | 0.985247 |
| 30 | 9 | -3 | 0.985309 |
| 51 | 33 | -9 | 0.986734 |
| -30 | -27 | -15 | 0.98775 |
| -51 | 15 | -18 | 0.988946 |
| -3 | -99 | -6 | 0.989418 |
| -42 | -66 | -45 | 0.989894 |
| -18 | -75 | -33 | 0.989978 |
| 45 | -15 | -36 | 0.991074 |
| -45 | -69 | -39 | 0.992113 |
| -57 | -27 | 0 | 0.993782 |
| -3 | -102 | -6 | 0.994359 |
| -18 | -12 | -30 | 0.995162 |
| -30 | -15 | -3 | 0.996243 |
| 39 | 42 | -15 | 0.996554 |
| -24 | -66 | -30 | 0.997812 |
| -33 | 36 | -12 | 0.998604 |
| -60 | -33 | 0 | 0.999598 |
| -36 | 12 | -42 | 0.999946 |
|  |  |  |  |

Notes: RBVC, retinal blood vessel curve.

**Table S6. There was no significant correlation between the cognition-FAZ area- ReHo in HC and SCD patients.**

| X (mm) | Y (mm) | Z (mm) | P_FWE_ |
| --- | --- | --- | --- |
| 36 | 21 | -24 | 0.052686 |
| -36 | 15 | 39 | 0.068539 |
| 51 | 12 | -15 | 0.082065 |
| 48 | 9 | -12 | 0.08453 |
| 51 | 6 | -12 | 0.109227 |
| -45 | -12 | -21 | 0.113588 |
| -48 | 0 | -9 | 0.115374 |
| 27 | -39 | -24 | 0.118554 |
| -36 | -60 | 36 | 0.118748 |
| 39 | 21 | -24 | 0.119623 |
| -45 | 3 | -9 | 0.120631 |
| 48 | 12 | -15 | 0.137417 |
| 36 | 9 | -9 | 0.138022 |
| 33 | 24 | -27 | 0.146035 |
| 48 | 6 | -12 | 0.153559 |
| -48 | 3 | -12 | 0.154411 |
| 30 | -60 | -30 | 0.157253 |
| -36 | -60 | 39 | 0.169513 |
| 48 | 9 | -15 | 0.205141 |
| 45 | -72 | -30 | 0.212926 |
| 51 | 9 | -15 | 0.228363 |
| 30 | -39 | -27 | 0.230722 |
| -45 | 6 | -9 | 0.236105 |
| 27 | -39 | -27 | 0.244587 |
| -51 | 0 | -12 | 0.248357 |
| -51 | 0 | -9 | 0.254506 |
| -36 | -57 | 36 | 0.276192 |
| -48 | 6 | -9 | 0.277156 |
| 27 | -33 | -27 | 0.279855 |
| -51 | 3 | -12 | 0.282092 |
| 27 | -63 | -30 | 0.285105 |
| -39 | 15 | 39 | 0.286466 |
| -9 | -12 | 33 | 0.288124 |
| 54 | 12 | -12 | 0.293153 |
| -12 | -42 | 45 | 0.297258 |
| 51 | 6 | -15 | 0.305246 |
| -21 | -54 | -57 | 0.323818 |
| 48 | 12 | -12 | 0.32532 |
| 27 | -36 | -24 | 0.32808 |
| 51 | 15 | -15 | 0.329783 |
| -15 | -51 | 51 | 0.333223 |
| 45 | -60 | -57 | 0.333315 |
| 36 | 24 | -27 | 0.342777 |
| -24 | 21 | 63 | 0.34433 |
| 15 | -42 | -60 | 0.344792 |
| -42 | -12 | -21 | 0.347361 |
| 33 | 21 | -27 | 0.351044 |
| 30 | -63 | -30 | 0.362601 |
| 27 | -36 | -30 | 0.363236 |
| -39 | -60 | 39 | 0.369052 |
| 30 | 39 | 45 | 0.388153 |
| 45 | -48 | -30 | 0.389266 |
| -45 | 3 | -6 | 0.389497 |
| -36 | 15 | 36 | 0.391712 |
| 54 | 9 | -9 | 0.398637 |
| 15 | -42 | -63 | 0.399558 |
| -48 | 6 | -12 | 0.401895 |
| -21 | -69 | 33 | 0.402272 |
| -36 | -57 | 39 | 0.403966 |
| 18 | -42 | -60 | 0.411009 |
| -9 | -9 | 33 | 0.414684 |
| 30 | -60 | -27 | 0.423523 |
| 33 | 42 | 42 | 0.427019 |
| 0 | -45 | -54 | 0.428984 |
| 45 | -57 | -57 | 0.437641 |
| -51 | 3 | -9 | 0.445198 |
| -12 | -39 | 57 | 0.446937 |
| 51 | 3 | -12 | 0.451576 |
| -45 | -12 | -18 | 0.452607 |
| -15 | -54 | 51 | 0.454546 |
| 36 | 21 | -27 | 0.456366 |
| -36 | -63 | 36 | 0.465683 |
| 42 | -60 | -54 | 0.467203 |
| -21 | -54 | -54 | 0.469214 |
| 39 | -60 | -57 | 0.477831 |
| -48 | 0 | -12 | 0.481152 |
| 39 | 21 | -21 | 0.493834 |
| 12 | -60 | -21 | 0.49554 |
| 15 | -60 | -21 | 0.497601 |
| 27 | -42 | -24 | 0.503285 |
| 42 | -60 | -57 | 0.51038 |
| -39 | -57 | 39 | 0.512103 |
| -12 | -51 | 51 | 0.514981 |
| -45 | -15 | -15 | 0.520185 |
| 39 | 24 | -24 | 0.533741 |
| 39 | -60 | -54 | 0.535255 |
| -9 | -39 | 57 | 0.538714 |
| -48 | 9 | -12 | 0.539098 |
| -21 | -57 | -54 | 0.545822 |
| 45 | 6 | -12 | 0.556201 |
| 27 | -39 | -30 | 0.559823 |
| 33 | 39 | 45 | 0.561118 |
| 3 | -45 | -54 | 0.563309 |
| -18 | -54 | -57 | 0.566432 |
| -42 | -15 | -15 | 0.566518 |
| 45 | -69 | -30 | 0.568528 |
| 51 | 15 | -12 | 0.575082 |
| -39 | 15 | 36 | 0.575654 |
| -42 | -15 | -18 | 0.577667 |
| 36 | -60 | -57 | 0.578162 |
| -3 | -60 | -3 | 0.587422 |
| 0 | -48 | -57 | 0.587889 |
| 0 | -45 | -57 | 0.588674 |
| 0 | -60 | -3 | 0.591306 |
| -6 | -48 | -54 | 0.596107 |
| 39 | -60 | -60 | 0.596433 |
| 54 | 9 | -12 | 0.600238 |
| 27 | -60 | -30 | 0.600779 |
| -3 | -48 | -57 | 0.603696 |
| 48 | 15 | -15 | 0.607447 |
| 36 | -60 | -60 | 0.612228 |
| -12 | -39 | 45 | 0.616417 |
| 33 | 39 | 42 | 0.617044 |
| 39 | 18 | -24 | 0.617857 |
| -45 | 6 | -6 | 0.621553 |
| -36 | -63 | 39 | 0.623533 |
| 33 | 24 | -24 | 0.625035 |
| 9 | -12 | 42 | 0.628559 |
| -15 | -51 | 48 | 0.629949 |
| -12 | -36 | 57 | 0.633018 |
| 36 | 24 | -21 | 0.638766 |
| 57 | 0 | 12 | 0.642702 |
| -3 | -60 | -6 | 0.647377 |
| 9 | -27 | -57 | 0.652746 |
| -12 | -45 | 45 | 0.655464 |
| 42 | -72 | -30 | 0.661892 |
| 36 | 9 | -6 | 0.671908 |
| 54 | 6 | -9 | 0.67339 |
| 45 | -51 | -30 | 0.683122 |
| 54 | 3 | -12 | 0.688327 |
| 42 | -57 | -54 | 0.689211 |
| 30 | -39 | -24 | 0.689516 |
| -27 | -99 | 3 | 0.692818 |
| 6 | -48 | -54 | 0.700003 |
| -6 | -48 | -57 | 0.708773 |
| -45 | 0 | -9 | 0.712915 |
| -18 | -57 | -54 | 0.71843 |
| 42 | -45 | -33 | 0.720313 |
| 42 | -57 | -57 | 0.729253 |
| -45 | -15 | -18 | 0.739844 |
| -42 | -12 | -18 | 0.741731 |
| 12 | 6 | -30 | 0.743009 |
| 3 | -45 | -57 | 0.744949 |
| -3 | -45 | -54 | 0.750271 |
| -21 | 21 | 60 | 0.751289 |
| 36 | -60 | -54 | 0.75647 |
| 3 | -48 | -57 | 0.764254 |
| 18 | -42 | -57 | 0.766907 |
| 33 | 42 | 45 | 0.774277 |
| 3 | -48 | -54 | 0.77991 |
| 51 | 9 | -9 | 0.783444 |
| 48 | 6 | -15 | 0.789217 |
| 15 | -57 | -21 | 0.790966 |
| -3 | -63 | -3 | 0.792573 |
| -48 | -12 | -21 | 0.80272 |
| 9 | -27 | -60 | 0.804408 |
| 54 | 3 | -9 | 0.804846 |
| -48 | 12 | -12 | 0.812635 |
| -9 | -36 | 57 | 0.812701 |
| -3 | -63 | -6 | 0.813747 |
| -42 | -15 | -21 | 0.816949 |
| 15 | 6 | -30 | 0.821407 |
| 27 | -60 | -27 | 0.826784 |
| -24 | -33 | 57 | 0.826797 |
| -3 | -66 | -54 | 0.831907 |
| -21 | 21 | 63 | 0.838788 |
| 30 | 39 | 42 | 0.839003 |
| -24 | -33 | 60 | 0.839049 |
| 45 | 9 | -12 | 0.840172 |
| -12 | -39 | 48 | 0.844153 |
| 39 | 21 | -3 | 0.849173 |
| -12 | -42 | 48 | 0.857382 |
| -33 | -57 | 36 | 0.859771 |
| -36 | -36 | 54 | 0.868527 |
| 27 | -66 | -30 | 0.871839 |
| 33 | 21 | -24 | 0.876596 |
| -42 | 3 | -6 | 0.88877 |
| -33 | -60 | 36 | 0.891284 |
| -12 | -48 | 48 | 0.896327 |
| -6 | -21 | 48 | 0.896696 |
| 39 | -42 | -36 | 0.900434 |
| -6 | -3 | 36 | 0.901667 |
| -12 | -51 | 54 | 0.904709 |
| -12 | -42 | 42 | 0.906815 |
| 48 | 3 | -12 | 0.913182 |
| -33 | -42 | -45 | 0.913519 |
| 39 | -45 | -36 | 0.91807 |
| 30 | -33 | -27 | 0.922418 |
| 30 | -36 | -30 | 0.922907 |
| -45 | -54 | -24 | 0.927515 |
| -12 | -36 | 51 | 0.927716 |
| 30 | -39 | -30 | 0.930252 |
| 24 | -36 | -30 | 0.9312 |
| 42 | -54 | 36 | 0.931565 |
| -18 | -54 | -54 | 0.932342 |
| -15 | -36 | 51 | 0.933513 |
| 30 | 42 | 42 | 0.934482 |
| -33 | -36 | 54 | 0.935472 |
| 12 | -27 | -60 | 0.935479 |
| 24 | -36 | -27 | 0.93747 |
| -39 | 18 | 36 | 0.938076 |
| 39 | 18 | -27 | 0.940362 |
| -24 | -54 | -57 | 0.942813 |
| 39 | -42 | -33 | 0.94497 |
| 12 | -63 | -21 | 0.94958 |
| -12 | -39 | 42 | 0.950231 |
| -9 | -12 | 36 | 0.951126 |
| -21 | 24 | 63 | 0.960143 |
| -6 | -66 | -54 | 0.960158 |
| -9 | -39 | 48 | 0.96023 |
| 30 | -66 | -30 | 0.964652 |
| 39 | -57 | 36 | 0.96642 |
| -24 | -30 | 57 | 0.96798 |
| -9 | -99 | 15 | 0.968279 |
| 45 | 6 | -9 | 0.969992 |
| 6 | -12 | 69 | 0.972849 |
| -15 | -54 | 54 | 0.973083 |
| -51 | -66 | 18 | 0.974426 |
| -24 | -36 | 60 | 0.980752 |
| -30 | -54 | -54 | 0.981253 |
| 9 | -12 | 39 | 0.981384 |
| 54 | 6 | -12 | 0.98171 |
| -9 | -99 | 12 | 0.981901 |
| 6 | -60 | -54 | 0.983284 |
| 54 | -36 | 21 | 0.983965 |
| -18 | -57 | -57 | 0.984757 |
| 51 | 3 | -15 | 0.984761 |
| -39 | -15 | -15 | 0.985407 |
| -42 | 6 | -6 | 0.985863 |
| 45 | 3 | -9 | 0.986166 |
| -24 | 21 | 60 | 0.991203 |
| -15 | -48 | 45 | 0.993386 |
| 30 | 36 | 45 | 0.9937 |
| 6 | -12 | 45 | 0.997763 |
| -9 | -15 | 36 | 0.999066 |
| -3 | -69 | -57 | 0.999319 |
| -48 | 12 | -15 | 0.999464 |
|  |  |  |  |

Notes: FAZ, Foveal Avascular Zone.

**Table S7. There was no significant correlation between the cognition-FAZ perimeter-ReHo in HC and SCD patients.**

| X (mm) | Y (mm) | Z (mm) | P_FWE_ |
| --- | --- | --- | --- |
| 12 | -60 | -21 | 0.068348 |
| -36 | -54 | 36 | 0.071014 |
| -39 | -60 | 39 | 0.080178 |
| -36 | -57 | 33 | 0.091789 |
| -39 | -57 | 39 | 0.097457 |
| -15 | -51 | 51 | 0.103824 |
| 18 | -45 | -63 | 0.118709 |
| -39 | -57 | 36 | 0.13328 |
| -36 | -63 | 36 | 0.134937 |
| 12 | -57 | -21 | 0.139046 |
| -33 | -57 | 36 | 0.141612 |
| 36 | 24 | -24 | 0.152091 |
| 36 | 21 | -24 | 0.159646 |
| 18 | -42 | -60 | 0.162994 |
| -36 | -54 | 33 | 0.177502 |
| -39 | -60 | 36 | 0.18551 |
| -15 | -54 | 51 | 0.196441 |
| -36 | -57 | 39 | 0.206216 |
| 39 | 21 | -24 | 0.207068 |
| -51 | 0 | -12 | 0.223396 |
| 33 | 39 | 45 | 0.224525 |
| 36 | 9 | -9 | 0.226488 |
| 39 | 21 | -21 | 0.244241 |
| -39 | -63 | 39 | 0.254724 |
| 27 | -33 | -27 | 0.254908 |
| 30 | 39 | 45 | 0.255852 |
| -36 | -60 | 39 | 0.263648 |
| 21 | -42 | -60 | 0.272166 |
| -51 | 3 | -12 | 0.274632 |
| -36 | -60 | 33 | 0.275497 |
| 15 | -42 | -60 | 0.286459 |
| 30 | -66 | -30 | 0.293428 |
| -39 | -54 | 36 | 0.312436 |
| -51 | -66 | 18 | 0.324342 |
| 3 | -48 | -57 | 0.324701 |
| 39 | -57 | 33 | 0.332439 |
| 18 | -45 | -60 | 0.335415 |
| 33 | 39 | 42 | 0.343401 |
| -12 | -42 | 45 | 0.349386 |
| -48 | 3 | -12 | 0.351233 |
| -12 | -51 | 51 | 0.36644 |
| 3 | -45 | -57 | 0.372231 |
| 0 | -45 | -57 | 0.37372 |
| 57 | 0 | 12 | 0.385055 |
| 30 | 36 | 45 | 0.396298 |
| 51 | 6 | -15 | 0.398013 |
| -33 | -60 | 36 | 0.401339 |
| -33 | -54 | 36 | 0.41163 |
| 42 | -57 | 36 | 0.414251 |
| -36 | -54 | 39 | 0.416969 |
| 51 | 9 | -12 | 0.429398 |
| 30 | -60 | -27 | 0.43095 |
| 33 | 42 | 42 | 0.434937 |
| 9 | -60 | -21 | 0.440563 |
| 36 | 24 | -21 | 0.446993 |
| 42 | -57 | 33 | 0.447866 |
| -48 | 3 | -9 | 0.448335 |
| 33 | -48 | 54 | 0.457866 |
| -15 | -54 | 54 | 0.470504 |
| -51 | 0 | -9 | 0.474413 |
| 15 | -60 | -21 | 0.498364 |
| 39 | -57 | 36 | 0.50136 |
| 33 | 36 | 45 | 0.510433 |
| -48 | 0 | -12 | 0.511703 |
| 3 | -45 | -54 | 0.513439 |
| -21 | -54 | -57 | 0.517925 |
| 42 | -60 | 33 | 0.519086 |
| 39 | -60 | 33 | 0.523723 |
| -15 | -51 | 54 | 0.525375 |
| -33 | -54 | 33 | 0.528925 |
| -12 | -51 | 54 | 0.533493 |
| 15 | -57 | -21 | 0.540348 |
| -15 | -51 | 48 | 0.545533 |
| -51 | -21 | 33 | 0.548294 |
| 15 | 3 | -24 | 0.551841 |
| 33 | 39 | 48 | 0.555918 |
| -39 | -54 | 39 | 0.555954 |
| 39 | 24 | -24 | 0.563186 |
| 3 | -48 | -54 | 0.568236 |
| 36 | 21 | -21 | 0.578287 |
| 51 | 12 | -12 | 0.578576 |
| 30 | -69 | -30 | 0.589768 |
| -33 | -57 | 33 | 0.590543 |
| 12 | -60 | -18 | 0.59334 |
| -39 | -60 | 42 | 0.606515 |
| 51 | -42 | 39 | 0.606903 |
| 54 | 12 | -12 | 0.612573 |
| 9 | 54 | 48 | 0.623901 |
| 12 | -63 | -21 | 0.625414 |
| 12 | -57 | -18 | 0.626361 |
| 33 | 36 | 48 | 0.635546 |
| 12 | 6 | -27 | 0.636687 |
| -48 | 0 | -9 | 0.642173 |
| 30 | 39 | 48 | 0.647955 |
| 15 | -42 | -63 | 0.654243 |
| 12 | -54 | -21 | 0.659577 |
| -36 | -63 | 39 | 0.665247 |
| 0 | -48 | -57 | 0.67168 |
| 51 | 6 | -12 | 0.672901 |
| 6 | -48 | -54 | 0.685121 |
| -39 | -63 | 36 | 0.686444 |
| -9 | -39 | 45 | 0.690562 |
| -15 | -69 | 39 | 0.69566 |
| 39 | -60 | 36 | 0.699392 |
| 51 | 9 | -15 | 0.702599 |
| 21 | -45 | -60 | 0.713612 |
| 0 | -45 | -54 | 0.717265 |
| 12 | -27 | -63 | 0.71834 |
| -21 | -69 | 30 | 0.719061 |
| -42 | -60 | 42 | 0.720007 |
| -51 | 3 | -9 | 0.720636 |
| -24 | -39 | 60 | 0.725699 |
| -18 | -51 | 51 | 0.729432 |
| 30 | -69 | -27 | 0.731764 |
| 30 | 36 | 48 | 0.734295 |
| 33 | -60 | -27 | 0.736147 |
| 24 | -33 | 60 | 0.739648 |
| 30 | -63 | -30 | 0.746613 |
| 9 | -60 | -18 | 0.752905 |
| 39 | -54 | 33 | 0.761856 |
| 42 | -54 | 36 | 0.764473 |
| 6 | -12 | 42 | 0.770918 |
| 45 | 42 | -21 | 0.776219 |
| -9 | -3 | 36 | 0.778002 |
| -12 | -39 | 45 | 0.783263 |
| -39 | -57 | 42 | 0.789707 |
| -51 | -66 | 15 | 0.790979 |
| 24 | -36 | 60 | 0.794718 |
| 36 | 9 | -6 | 0.796729 |
| 12 | 6 | -30 | 0.797008 |
| 39 | 21 | 0 | 0.803877 |
| 9 | -57 | -18 | 0.80392 |
| 30 | -33 | -27 | 0.806772 |
| 3 | -51 | -57 | 0.815571 |
| -57 | -57 | 15 | 0.816779 |
| -51 | -63 | 18 | 0.818851 |
| -48 | -66 | 18 | 0.823232 |
| 33 | -69 | -30 | 0.823559 |
| -12 | -54 | 54 | 0.829123 |
| 9 | -63 | -21 | 0.831449 |
| 30 | -60 | -24 | 0.831766 |
| 36 | -48 | 57 | 0.835621 |
| 33 | 36 | 51 | 0.835625 |
| -21 | -42 | 60 | 0.836842 |
| 27 | -66 | -30 | 0.84164 |
| 42 | -54 | 33 | 0.848543 |
| 51 | -39 | 39 | 0.848798 |
| -51 | 0 | -15 | 0.851877 |
| -66 | -33 | 6 | 0.85335 |
| -21 | -69 | 33 | 0.854845 |
| 33 | -66 | -30 | 0.861349 |
| -69 | -33 | 6 | 0.86201 |
| -18 | -54 | -57 | 0.863374 |
| 9 | -57 | -21 | 0.864364 |
| 6 | -81 | -51 | 0.865103 |
| 15 | -45 | -60 | 0.866959 |
| -18 | -57 | 57 | 0.867839 |
| -12 | -69 | 42 | 0.869563 |
| -54 | -57 | 15 | 0.869624 |
| -3 | -66 | -54 | 0.871355 |
| 15 | -54 | -21 | 0.87531 |
| -18 | -54 | -60 | 0.875424 |
| 27 | -57 | 42 | 0.876349 |
| 39 | 24 | -21 | 0.876499 |
| 51 | 12 | -15 | 0.877091 |
| -15 | -69 | 42 | 0.878366 |
| 33 | 42 | 39 | 0.878804 |
| -18 | -57 | 60 | 0.880966 |
| 30 | 39 | 42 | 0.890228 |
| 39 | 18 | -24 | 0.892979 |
| 12 | -60 | -24 | 0.896148 |
| 12 | -57 | -24 | 0.898544 |
| -30 | -63 | 66 | 0.900142 |
| 12 | 3 | -27 | 0.900313 |
| -21 | -51 | -57 | 0.902659 |
| 30 | -60 | -30 | 0.903749 |
| 27 | -57 | 45 | 0.905024 |
| -12 | -54 | 51 | 0.908887 |
| -42 | -15 | -15 | 0.910931 |
| -9 | -48 | -57 | 0.913837 |
| 51 | 3 | -15 | 0.918356 |
| 15 | -51 | -21 | 0.918829 |
| 27 | -33 | -24 | 0.918885 |
| -21 | -54 | -60 | 0.920286 |
| -63 | -33 | 6 | 0.920953 |
| 30 | -66 | -27 | 0.924319 |
| -24 | -36 | 60 | 0.924439 |
| -3 | -69 | -54 | 0.928082 |
| 9 | -15 | 42 | 0.928712 |
| 15 | -57 | -24 | 0.928835 |
| 39 | 18 | 18 | 0.931578 |
| 33 | 42 | 45 | 0.932903 |
| 36 | 21 | 0 | 0.933719 |
| 36 | 39 | 42 | 0.935224 |
| 36 | -48 | 54 | 0.935585 |
| -54 | -60 | 15 | 0.940965 |
| -57 | 0 | 42 | 0.94098 |
| 21 | -36 | 60 | 0.942085 |
| 42 | -72 | -57 | 0.944525 |
| -12 | -69 | 39 | 0.944856 |
| -57 | 3 | 42 | 0.945217 |
| 42 | -60 | 36 | 0.945295 |
| -9 | 0 | 33 | 0.948094 |
| 3 | -54 | -57 | 0.94949 |
| -51 | -24 | 36 | 0.950036 |
| -6 | -3 | 36 | 0.950886 |
| -3 | -45 | -57 | 0.950891 |
| 6 | -78 | -51 | 0.953597 |
| 27 | -69 | -27 | 0.956265 |
| 15 | -3 | -6 | 0.956806 |
| -12 | -45 | 45 | 0.957318 |
| -24 | -36 | 57 | 0.958129 |
| 6 | -51 | -54 | 0.958604 |
| -12 | -42 | 42 | 0.95925 |
| -57 | 3 | 39 | 0.960032 |
| 48 | 6 | -12 | 0.961355 |
| -48 | 6 | -9 | 0.962033 |
| 3 | -51 | -54 | 0.962046 |
| -54 | -63 | 18 | 0.963443 |
| 39 | 21 | -3 | 0.964827 |
| -36 | -36 | 54 | 0.967999 |
| -15 | -57 | 51 | 0.968478 |
| 45 | -72 | -30 | 0.971045 |
| 36 | 12 | -9 | 0.971615 |
| 24 | -36 | 63 | 0.971754 |
| 9 | -12 | 42 | 0.971983 |
| -9 | -3 | 33 | 0.976038 |
| -42 | -63 | 42 | 0.97721 |
| -9 | -36 | -30 | 0.978492 |
| -18 | -51 | -60 | 0.980356 |
| 57 | 0 | 15 | 0.983672 |
| 42 | 18 | 18 | 0.984216 |
| 54 | 9 | -12 | 0.984564 |
| -48 | 6 | -12 | 0.985179 |
| 36 | 12 | -6 | 0.98611 |
| 30 | -63 | -27 | 0.988453 |
| 33 | -48 | 57 | 0.989486 |
| 27 | -36 | -27 | 0.993931 |
| 51 | 3 | -12 | 0.994985 |
| -42 | -15 | -18 | 0.998938 |
|  |  |  |  |

Notes: FAZ, Foveal Avascular Zone.

**Table S8. There was no significant correlation between the cognition-GCL-IPL – ReHo in HC and SCD patients.**

| X (mm) | Y (mm) | Z (mm) | P_FWE_ |
| --- | --- | --- | --- |
| 69 | 3 | 12 | 0.057901 |
| 6 | 42 | 33 | 0.059632 |
| 12 | -63 | 48 | 0.063798 |
| -3 | 0 | 27 | 0.064353 |
| 9 | 33 | -33 | 0.069142 |
| 12 | -60 | 48 | 0.071804 |
| -3 | -63 | -48 | 0.08108 |
| 54 | -60 | -12 | 0.08289 |
| 9 | -63 | 48 | 0.083672 |
| 12 | -63 | 51 | 0.087534 |
| 54 | -63 | -12 | 0.099823 |
| -3 | -3 | 27 | 0.109254 |
| 54 | -63 | -9 | 0.109928 |
| 9 | -63 | 51 | 0.120946 |
| 12 | -60 | 51 | 0.152196 |
| 66 | 3 | 15 | 0.160877 |
| 54 | -60 | -9 | 0.187018 |
| 57 | -63 | 9 | 0.188218 |
| -48 | 9 | -27 | 0.192581 |
| -6 | -66 | -48 | 0.203318 |
| 30 | -24 | -33 | 0.213964 |
| 15 | -39 | -36 | 0.233266 |
| -9 | -63 | -48 | 0.238002 |
| 9 | -60 | 51 | 0.243002 |
| -51 | -63 | 6 | 0.243674 |
| 6 | 36 | -30 | 0.24667 |
| -6 | -72 | 48 | 0.250341 |
| 60 | -66 | 9 | 0.251689 |
| 60 | -66 | 6 | 0.265134 |
| 9 | -60 | 48 | 0.269782 |
| 3 | 45 | 33 | 0.276956 |
| -6 | -3 | 27 | 0.282231 |
| 54 | -9 | -9 | 0.282913 |
| -6 | 0 | 30 | 0.283473 |
| -42 | 12 | -33 | 0.283913 |
| -24 | 21 | -24 | 0.284248 |
| 6 | 30 | -30 | 0.289577 |
| -6 | -72 | 45 | 0.292733 |
| 69 | -42 | -18 | 0.298305 |
| 3 | 42 | 33 | 0.301577 |
| -24 | 21 | -27 | 0.309319 |
| 12 | -66 | 51 | 0.324681 |
| 60 | -63 | 9 | 0.334291 |
| -6 | -63 | -51 | 0.335562 |
| 57 | -60 | -9 | 0.354878 |
| 15 | -39 | -33 | 0.35651 |
| 54 | -66 | -12 | 0.35752 |
| 63 | -66 | 6 | 0.360852 |
| 30 | -24 | -36 | 0.374591 |
| -6 | -69 | 48 | 0.382528 |
| 6 | 30 | -33 | 0.387019 |
| -33 | 30 | 42 | 0.426701 |
| 33 | -24 | -36 | 0.426729 |
| 9 | 36 | -33 | 0.429663 |
| 54 | -66 | -9 | 0.437217 |
| 63 | -66 | 9 | 0.446781 |
| 51 | -63 | -12 | 0.446802 |
| 6 | 3 | 63 | 0.459017 |
| 12 | -36 | -45 | 0.471923 |
| 33 | -21 | -36 | 0.479191 |
| 33 | -24 | -33 | 0.483924 |
| 69 | -45 | -15 | 0.490475 |
| 66 | 3 | 12 | 0.492031 |
| 9 | -63 | 54 | 0.497707 |
| 57 | -66 | 6 | 0.501449 |
| -54 | -63 | 6 | 0.501951 |
| 57 | -66 | 9 | 0.504586 |
| 12 | -60 | 45 | 0.517885 |
| 57 | -60 | 9 | 0.527406 |
| -3 | -60 | -48 | 0.530323 |
| 18 | -39 | -36 | 0.535241 |
| 6 | 24 | -30 | 0.540396 |
| 9 | -66 | 48 | 0.543234 |
| 57 | -63 | -9 | 0.550796 |
| 9 | -66 | 51 | 0.55556 |
| -3 | -66 | -48 | 0.559766 |
| -45 | 12 | -33 | 0.56643 |
| 69 | 0 | 15 | 0.572551 |
| 9 | -60 | 54 | 0.578695 |
| -24 | 24 | -27 | 0.581639 |
| 9 | 33 | -30 | 0.613519 |
| -6 | -72 | 42 | 0.614881 |
| 60 | -63 | 6 | 0.616743 |
| -48 | 9 | -30 | 0.619768 |
| 6 | 45 | 33 | 0.623981 |
| -3 | 0 | 30 | 0.624468 |
| 69 | -42 | -15 | 0.634446 |
| 6 | 42 | 30 | 0.636895 |
| 3 | 3 | 63 | 0.640172 |
| 6 | 27 | -30 | 0.642631 |
| 3 | 45 | 30 | 0.645032 |
| -33 | 30 | 39 | 0.656385 |
| -51 | -66 | 6 | 0.660537 |
| -6 | -66 | -51 | 0.661055 |
| 51 | -60 | -12 | 0.670744 |
| -24 | 24 | -24 | 0.676063 |
| 21 | -21 | -6 | 0.68774 |
| -9 | -69 | 48 | 0.690615 |
| 69 | 0 | 12 | 0.691296 |
| 6 | 3 | 66 | 0.699964 |
| 69 | -27 | 6 | 0.700361 |
| 60 | -60 | 9 | 0.705344 |
| -45 | 12 | -30 | 0.709569 |
| -48 | 12 | -30 | 0.7103 |
| 12 | -66 | 48 | 0.717655 |
| 6 | 27 | -33 | 0.71771 |
| -3 | 3 | 27 | 0.719251 |
| 69 | -30 | 6 | 0.724813 |
| -30 | 30 | 42 | 0.72766 |
| 57 | -66 | -9 | 0.728286 |
| 57 | -63 | 6 | 0.73138 |
| 12 | -36 | -42 | 0.732133 |
| -54 | -66 | 6 | 0.735909 |
| 57 | -66 | -12 | 0.737057 |
| 6 | 45 | 30 | 0.737793 |
| 3 | 3 | 60 | 0.742535 |
| -48 | 6 | -27 | 0.746299 |
| -48 | 12 | -27 | 0.74709 |
| 60 | -69 | 6 | 0.749493 |
| 12 | -60 | 54 | 0.753648 |
| -6 | -3 | 30 | 0.756555 |
| -21 | 21 | -24 | 0.761311 |
| -12 | 63 | -12 | 0.761868 |
| -21 | 12 | 60 | 0.774528 |
| -12 | 63 | -15 | 0.775156 |
| -6 | -75 | 48 | 0.781331 |
| 51 | -72 | 3 | 0.781745 |
| 69 | -45 | -18 | 0.78938 |
| -51 | -63 | 3 | 0.79187 |
| -3 | -6 | 27 | 0.79341 |
| 57 | -57 | -9 | 0.794947 |
| -6 | -60 | -48 | 0.796014 |
| 21 | -24 | -6 | 0.803257 |
| -51 | -66 | 3 | 0.810361 |
| 57 | 42 | 6 | 0.811128 |
| 30 | -21 | -36 | 0.811558 |
| -24 | 18 | -24 | 0.818137 |
| 63 | -63 | 6 | 0.819279 |
| -3 | -63 | -51 | 0.828072 |
| -24 | 21 | -21 | 0.836039 |
| 57 | -69 | -12 | 0.836153 |
| 63 | -63 | 9 | 0.838923 |
| 12 | -84 | -45 | 0.84033 |
| -3 | -3 | 30 | 0.8514 |
| -42 | 15 | -33 | 0.854787 |
| 51 | -9 | -9 | 0.859367 |
| -6 | -75 | 45 | 0.860593 |
| 12 | -69 | 51 | 0.864006 |
| 15 | -42 | -33 | 0.865943 |
| -3 | -66 | -51 | 0.872433 |
| 30 | -21 | -33 | 0.873285 |
| 12 | -63 | 54 | 0.876149 |
| 72 | -27 | 6 | 0.879161 |
| 69 | -45 | -12 | 0.880508 |
| -9 | -72 | 48 | 0.884009 |
| 3 | 33 | -33 | 0.885165 |
| -54 | -66 | 3 | 0.886233 |
| 69 | -39 | -18 | 0.888193 |
| 66 | -42 | -15 | 0.889337 |
| -12 | 60 | -12 | 0.894227 |
| 18 | -24 | -6 | 0.896291 |
| -6 | -69 | 45 | 0.896446 |
| -12 | 66 | -18 | 0.903745 |
| 69 | -30 | 9 | 0.907166 |
| 54 | -6 | -12 | 0.908979 |
| -18 | -15 | -51 | 0.911523 |
| 57 | -60 | -12 | 0.911878 |
| -6 | -72 | 51 | 0.913586 |
| 57 | -63 | 12 | 0.913849 |
| 15 | -36 | -36 | 0.919454 |
| 60 | 0 | -39 | 0.920331 |
| 66 | 0 | 15 | 0.925853 |
| 54 | 42 | 6 | 0.926177 |
| -6 | -69 | 51 | 0.927342 |
| -12 | 66 | -15 | 0.927961 |
| -9 | -9 | 72 | 0.928723 |
| -3 | 48 | -15 | 0.9299 |
| -12 | 66 | -12 | 0.933273 |
| 3 | 6 | 60 | 0.934883 |
| 12 | 0 | -39 | 0.935187 |
| 12 | -57 | 48 | 0.937784 |
| 9 | 30 | -33 | 0.939834 |
| 57 | -63 | -12 | 0.941034 |
| -54 | 9 | -36 | 0.941819 |
| 54 | -78 | 0 | 0.942534 |
| -9 | -66 | -48 | 0.943854 |
| 9 | -60 | 45 | 0.946974 |
| -6 | -75 | 42 | 0.951181 |
| -51 | 9 | -36 | 0.95155 |
| 9 | -69 | 51 | 0.954302 |
| 54 | -75 | 0 | 0.954571 |
| -3 | -72 | 45 | 0.955457 |
| 15 | -84 | -45 | 0.955873 |
| 12 | -39 | -33 | 0.957712 |
| -6 | 3 | 27 | 0.960387 |
| 66 | -45 | -15 | 0.960854 |
| 48 | 6 | 15 | 0.962426 |
| 60 | -63 | 12 | 0.964785 |
| 12 | -33 | -45 | 0.966429 |
| -51 | -21 | -9 | 0.967795 |
| 57 | -60 | -6 | 0.967999 |
| 33 | -21 | -33 | 0.969202 |
| 18 | -27 | -6 | 0.969203 |
| -51 | 6 | -36 | 0.969587 |
| 60 | -69 | -12 | 0.971103 |
| 57 | -57 | -6 | 0.971304 |
| -30 | -78 | -24 | 0.971702 |
| 3 | 6 | 63 | 0.973662 |
| 69 | 3 | 9 | 0.974363 |
| 6 | 21 | -30 | 0.975471 |
| 0 | 3 | 60 | 0.976072 |
| 9 | -36 | -45 | 0.977046 |
| -18 | 15 | 63 | 0.977611 |
| 60 | -33 | 27 | 0.978538 |
| -36 | 12 | 42 | 0.979063 |
| 0 | -96 | -21 | 0.979086 |
| -27 | -36 | -45 | 0.979488 |
| 15 | -42 | -36 | 0.979666 |
| -3 | -72 | 48 | 0.980086 |
| -9 | -69 | 51 | 0.980504 |
| 0 | 6 | 60 | 0.981027 |
| 15 | -36 | -45 | 0.981856 |
| -48 | 9 | -33 | 0.983037 |
| -9 | -27 | -6 | 0.983403 |
| -36 | 9 | 42 | 0.98484 |
| 12 | -63 | 45 | 0.985079 |
| 12 | -84 | -42 | 0.986342 |
| -15 | -6 | -45 | 0.987141 |
| -51 | 6 | -33 | 0.991639 |
| 51 | -60 | -9 | 0.993971 |
| 69 | -48 | -15 | 0.995226 |
| -30 | 30 | 39 | 0.99689 |
| -54 | 6 | -36 | 0.997562 |
| -54 | -63 | 3 | 0.998039 |
| 60 | -69 | 9 | 0.998299 |
| -24 | 27 | 42 | 0.998484 |
| 60 | -57 | 9 | 0.998597 |
| 66 | -45 | -12 | 0.999193 |
|  |  |  |  |

Notes: GCL, ganglion cell layer; IPL, inner plexiform layer.

**Table S9. There was no significant correlation between the cognition-RNFL- ReHo in HC and SCD patients.**

| X (mm) | Y (mm) | Z (mm) | P_FWE_ |
| --- | --- | --- | --- |
| 21 | -60 | -51 | 0.052907 |
| 15 | -36 | -45 | 0.054376 |
| 12 | -84 | -45 | 0.060676 |
| -6 | -63 | -51 | 0.061499 |
| -9 | -81 | -42 | 0.061923 |
| -60 | -12 | 48 | 0.062912 |
| -6 | -63 | -48 | 0.070832 |
| -6 | -84 | -39 | 0.07225 |
| 54 | -3 | -15 | 0.078879 |
| 6 | 39 | -33 | 0.094427 |
| 54 | -63 | -9 | 0.103834 |
| -21 | 3 | -27 | 0.104601 |
| -30 | -45 | -12 | 0.111643 |
| 6 | 36 | -30 | 0.113036 |
| 12 | -36 | -45 | 0.113093 |
| -27 | -54 | -21 | 0.113721 |
| -30 | -42 | -12 | 0.124873 |
| -3 | 0 | -27 | 0.124894 |
| -57 | -12 | 48 | 0.144371 |
| 6 | 33 | -33 | 0.151165 |
| -30 | -45 | -9 | 0.156526 |
| -6 | -69 | -48 | 0.179005 |
| -6 | -60 | -51 | 0.193053 |
| -6 | -48 | -51 | 0.196825 |
| -30 | -42 | -15 | 0.19719 |
| -12 | -81 | -42 | 0.207874 |
| -12 | -84 | -39 | 0.226928 |
| -24 | -54 | -21 | 0.232309 |
| -6 | -66 | -48 | 0.233234 |
| -15 | -84 | -42 | 0.238537 |
| -12 | 66 | -15 | 0.240606 |
| 15 | -84 | -45 | 0.242606 |
| 21 | -63 | -51 | 0.251654 |
| 12 | -87 | -45 | 0.254094 |
| 12 | -87 | -42 | 0.263701 |
| -33 | -42 | -15 | 0.276775 |
| 54 | -63 | -12 | 0.279449 |
| -12 | -84 | -45 | 0.280991 |
| 18 | -57 | 18 | 0.281222 |
| -18 | -54 | -24 | 0.314453 |
| -6 | -66 | -51 | 0.324387 |
| 21 | -57 | 15 | 0.336055 |
| 15 | -33 | -45 | 0.344388 |
| -6 | -72 | -48 | 0.347329 |
| -9 | -63 | -48 | 0.3524 |
| -9 | -87 | -42 | 0.356675 |
| -15 | -57 | -21 | 0.358867 |
| 3 | 39 | -33 | 0.365642 |
| -18 | -39 | -45 | 0.366907 |
| -9 | -75 | -45 | 0.369324 |
| -18 | -57 | -24 | 0.375406 |
| -3 | -72 | 48 | 0.385833 |
| -3 | -69 | 45 | 0.403991 |
| -3 | -72 | 45 | 0.405276 |
| 21 | -57 | 18 | 0.405428 |
| -6 | -84 | -42 | 0.407599 |
| -9 | -69 | -48 | 0.428307 |
| 54 | -66 | -9 | 0.43172 |
| 54 | -60 | -12 | 0.46237 |
| 21 | -60 | -48 | 0.466595 |
| -18 | -78 | -39 | 0.473154 |
| 45 | -54 | -18 | 0.483132 |
| -15 | -54 | -21 | 0.48517 |
| -3 | -69 | 42 | 0.486799 |
| 6 | 39 | -30 | 0.489012 |
| -9 | -66 | -48 | 0.496883 |
| 9 | -84 | -45 | 0.501464 |
| 12 | -33 | -45 | 0.502916 |
| -6 | -45 | -54 | 0.504574 |
| -12 | 66 | -18 | 0.505483 |
| -15 | -81 | -39 | 0.507108 |
| -3 | -72 | 42 | 0.518929 |
| 33 | -3 | -18 | 0.522254 |
| 15 | -84 | -42 | 0.526515 |
| -6 | -81 | -42 | 0.527278 |
| -6 | -72 | -45 | 0.536749 |
| -18 | -57 | -21 | 0.540087 |
| -27 | -57 | -21 | 0.540644 |
| 33 | -9 | -21 | 0.545406 |
| 18 | -57 | 15 | 0.547471 |
| 33 | -3 | -21 | 0.551513 |
| -27 | 6 | -21 | 0.571097 |
| -9 | -81 | -39 | 0.586584 |
| -6 | -81 | -39 | 0.587697 |
| 51 | -60 | -12 | 0.591331 |
| 54 | -60 | -9 | 0.603645 |
| 24 | -60 | -51 | 0.608003 |
| -9 | -69 | -51 | 0.61934 |
| -9 | -72 | -45 | 0.632908 |
| -3 | -60 | -51 | 0.637527 |
| -15 | -81 | -42 | 0.641253 |
| -27 | -54 | -24 | 0.642375 |
| 6 | 33 | -30 | 0.643376 |
| -24 | 3 | -27 | 0.66681 |
| -3 | -69 | 48 | 0.671372 |
| -30 | -42 | -9 | 0.692966 |
| 9 | -84 | -42 | 0.692968 |
| -12 | -87 | -42 | 0.696976 |
| -6 | -48 | -54 | 0.701234 |
| 9 | -87 | -45 | 0.702078 |
| -18 | -54 | -21 | 0.702969 |
| -15 | -54 | -24 | 0.706877 |
| -6 | -87 | -42 | 0.712992 |
| -51 | -72 | -9 | 0.723651 |
| 18 | -63 | -51 | 0.731836 |
| -6 | -69 | -51 | 0.733536 |
| 18 | -60 | -51 | 0.736182 |
| -12 | 66 | -12 | 0.738431 |
| -3 | -63 | -51 | 0.755621 |
| -9 | -66 | -51 | 0.756045 |
| 3 | 36 | -33 | 0.757699 |
| -15 | -57 | -24 | 0.760577 |
| -21 | 6 | -27 | 0.764319 |
| -27 | 6 | -18 | 0.769089 |
| -12 | -81 | -45 | 0.776106 |
| -6 | -75 | -45 | 0.776293 |
| -3 | -78 | 42 | 0.776296 |
| -15 | -84 | -45 | 0.781633 |
| -6 | -60 | -48 | 0.783421 |
| -24 | -57 | -21 | 0.785144 |
| -15 | -3 | -45 | 0.792011 |
| 24 | -54 | -18 | 0.801555 |
| -3 | 3 | -30 | 0.806218 |
| 0 | 0 | -27 | 0.806235 |
| -9 | -72 | -48 | 0.810502 |
| -30 | -45 | -15 | 0.815308 |
| -9 | -87 | -39 | 0.816668 |
| -6 | -42 | -51 | 0.828324 |
| 54 | -63 | -6 | 0.831125 |
| 21 | -60 | 18 | 0.832261 |
| 54 | -66 | -6 | 0.848552 |
| 0 | -78 | -45 | 0.851132 |
| 9 | 36 | -33 | 0.851275 |
| 15 | -36 | -42 | 0.853694 |
| -9 | -84 | -45 | 0.853762 |
| 18 | -63 | -48 | 0.855023 |
| 36 | 0 | -21 | 0.856381 |
| -12 | 63 | -15 | 0.860865 |
| -15 | -84 | -39 | 0.861115 |
| -24 | -54 | -24 | 0.872344 |
| -9 | -63 | -51 | 0.87281 |
| 18 | -60 | 18 | 0.874533 |
| 9 | 33 | -33 | 0.878173 |
| -12 | 63 | -12 | 0.879566 |
| 33 | -6 | -18 | 0.880213 |
| 33 | -6 | -21 | 0.880359 |
| -3 | 3 | -27 | 0.882501 |
| -9 | -78 | -42 | 0.886945 |
| -54 | -12 | 48 | 0.887791 |
| -21 | -54 | -21 | 0.889084 |
| 3 | 39 | -30 | 0.890274 |
| -9 | -81 | -45 | 0.890351 |
| 45 | -18 | -45 | 0.890467 |
| -18 | -75 | -39 | 0.891161 |
| 18 | -36 | -45 | 0.891584 |
| -15 | -78 | -39 | 0.897666 |
| 18 | -60 | -48 | 0.909134 |
| 9 | -87 | -42 | 0.909543 |
| 12 | -90 | -45 | 0.910623 |
| -30 | -69 | -51 | 0.916987 |
| 45 | -18 | -42 | 0.921393 |
| 51 | -3 | -18 | 0.924648 |
| -30 | -3 | -21 | 0.924702 |
| 33 | 0 | -21 | 0.925767 |
| 15 | -39 | -45 | 0.92842 |
| 21 | -6 | 18 | 0.932233 |
| 0 | -93 | 21 | 0.93266 |
| 15 | -87 | -42 | 0.933808 |
| 15 | -39 | -36 | 0.934339 |
| -27 | -69 | -51 | 0.934491 |
| 0 | -93 | 18 | 0.936749 |
| 3 | -78 | -45 | 0.938128 |
| 54 | -66 | -12 | 0.940154 |
| -9 | -78 | -45 | 0.942316 |
| -21 | -54 | -24 | 0.942469 |
| -21 | -57 | -24 | 0.94588 |
| -30 | 9 | -18 | 0.94679 |
| -12 | -81 | -39 | 0.946996 |
| 0 | 3 | -27 | 0.950957 |
| 3 | -93 | 21 | 0.952284 |
| -39 | -54 | 60 | 0.954566 |
| -27 | 9 | -18 | 0.954962 |
| -18 | -15 | -48 | 0.955992 |
| 42 | -18 | -42 | 0.956317 |
| -9 | -60 | -51 | 0.95755 |
| -18 | -84 | -42 | 0.95858 |
| -3 | -75 | 42 | 0.958904 |
| 51 | -63 | -12 | 0.961884 |
| -9 | -33 | -30 | 0.962754 |
| -15 | -87 | -42 | 0.963312 |
| -24 | -57 | -24 | 0.965328 |
| 54 | -3 | -12 | 0.967263 |
| -6 | -87 | -39 | 0.968403 |
| -42 | -54 | 60 | 0.97053 |
| -24 | 6 | -21 | 0.97125 |
| 12 | -36 | -42 | 0.971772 |
| -3 | -75 | 48 | 0.976162 |
| 57 | -63 | 9 | 0.977029 |
| -39 | -54 | 63 | 0.977797 |
| -36 | -3 | -51 | 0.978886 |
| -54 | 9 | -36 | 0.981631 |
| -33 | -42 | -12 | 0.981635 |
| 12 | -24 | -54 | 0.982559 |
| 3 | -93 | 18 | 0.982962 |
| -3 | -60 | -48 | 0.986185 |
| -9 | -69 | -45 | 0.986213 |
| 51 | -6 | -18 | 0.987221 |
| -24 | 6 | -24 | 0.987836 |
| 3 | -78 | -48 | 0.987924 |
| 57 | -60 | 9 | 0.98935 |
| -48 | -72 | -9 | 0.989778 |
| -27 | -45 | -15 | 0.991441 |
| 42 | -18 | -45 | 0.994192 |
| -21 | -57 | -21 | 0.994509 |
| 9 | 36 | -30 | 0.995309 |
| -3 | -72 | 51 | 0.995831 |
| 3 | -81 | -48 | 0.9971 |
| 51 | -6 | -15 | 0.999406 |
|  |  |  |  |

Notes: RNFL, retinal nerve fiber layer.

**Table S10. There was no significant correlation between the cognition-RBVC- ReHo in HC and SCD patients.**

| X (mm) | Y (mm) | Z (mm) | P_FWE_ |
| --- | --- | --- | --- |
| -3 | -81 | 51 | 0.051079 |
| -27 | 15 | -45 | 0.090356 |
| 9 | -15 | -33 | 0.116377 |
| -6 | -81 | 51 | 0.12129 |
| -39 | -54 | 60 | 0.13385 |
| -3 | -78 | 48 | 0.147115 |
| 9 | -12 | -33 | 0.158579 |
| -3 | -78 | 51 | 0.161255 |
| -6 | -78 | 51 | 0.225719 |
| -24 | 12 | -45 | 0.230493 |
| -39 | -48 | 57 | 0.243142 |
| -27 | 12 | -45 | 0.248717 |
| -30 | 15 | -45 | 0.265401 |
| -3 | -81 | 48 | 0.266939 |
| -39 | -54 | 57 | 0.270919 |
| -36 | -51 | 57 | 0.279538 |
| 33 | 0 | -6 | 0.29231 |
| 12 | -15 | -33 | 0.294088 |
| -36 | -51 | 60 | 0.316759 |
| -6 | -78 | 48 | 0.330561 |
| -6 | -81 | 48 | 0.332371 |
| 6 | -12 | -33 | 0.333313 |
| -30 | 12 | -45 | 0.333616 |
| -48 | -45 | 27 | 0.348457 |
| 21 | -36 | -6 | 0.360851 |
| -27 | 15 | -42 | 0.369927 |
| -36 | -48 | 60 | 0.374823 |
| -9 | -99 | 15 | 0.375439 |
| -9 | -102 | 15 | 0.375867 |
| -45 | -54 | 54 | 0.377883 |
| -27 | 12 | -42 | 0.381922 |
| -3 | -75 | 48 | 0.384747 |
| 18 | -36 | -6 | 0.392563 |
| 66 | -15 | -12 | 0.393209 |
| -36 | -54 | 57 | 0.410722 |
| -39 | -78 | 33 | 0.411116 |
| -36 | -48 | 57 | 0.42654 |
| -30 | 15 | -42 | 0.429718 |
| -45 | -54 | 51 | 0.448693 |
| 33 | -3 | -6 | 0.452811 |
| -36 | -54 | 60 | 0.452862 |
| -30 | 12 | -42 | 0.455035 |
| -6 | -99 | 15 | 0.455947 |
| -39 | -51 | 63 | 0.460706 |
| -30 | 9 | -42 | 0.462341 |
| -42 | -51 | 60 | 0.464348 |
| -3 | -78 | 45 | 0.475174 |
| -30 | 9 | -45 | 0.477086 |
| 6 | -15 | -33 | 0.481943 |
| -9 | -102 | 12 | 0.484472 |
| -42 | -51 | 57 | 0.511137 |
| -27 | 9 | -45 | 0.514002 |
| -6 | -96 | 15 | 0.516225 |
| 63 | -15 | -12 | 0.519921 |
| -9 | -78 | 51 | 0.521558 |
| -39 | -81 | 33 | 0.522124 |
| 69 | -15 | -12 | 0.522326 |
| -9 | -102 | 9 | 0.523521 |
| -12 | -102 | 15 | 0.524417 |
| -39 | -48 | 63 | 0.526626 |
| 12 | -12 | -33 | 0.536948 |
| -9 | -102 | 18 | 0.537698 |
| -33 | -78 | 45 | 0.538299 |
| -6 | -102 | 15 | 0.538893 |
| -24 | 39 | 39 | 0.543364 |
| -6 | -78 | 54 | 0.545214 |
| -42 | -54 | 54 | 0.548596 |
| -39 | -54 | 63 | 0.549243 |
| -39 | -81 | 30 | 0.549635 |
| -42 | -54 | 60 | 0.555724 |
| 0 | -72 | 48 | 0.556584 |
| -27 | -102 | -9 | 0.557726 |
| -39 | -51 | 54 | 0.565726 |
| 9 | -15 | -36 | 0.570301 |
| -27 | 9 | -42 | 0.573947 |
| -39 | -78 | 30 | 0.576221 |
| 33 | -39 | -15 | 0.579541 |
| -3 | -78 | 54 | 0.585317 |
| 63 | -18 | -12 | 0.589185 |
| -3 | -75 | 51 | 0.609077 |
| 36 | -3 | -6 | 0.612912 |
| -6 | -84 | 48 | 0.615201 |
| -9 | -99 | 12 | 0.61611 |
| -36 | -81 | 30 | 0.616432 |
| -9 | -81 | 51 | 0.620691 |
| -24 | 42 | 39 | 0.626377 |
| -9 | -99 | 18 | 0.629241 |
| -42 | -54 | 57 | 0.633971 |
| -3 | -9 | -36 | 0.640194 |
| -51 | -54 | -27 | 0.640353 |
| -36 | -81 | 33 | 0.652163 |
| -33 | -81 | 48 | 0.652665 |
| -6 | -75 | 51 | 0.652824 |
| -48 | -54 | 54 | 0.653026 |
| -36 | -84 | 33 | 0.65936 |
| 3 | -45 | -57 | 0.662781 |
| -42 | -78 | 33 | 0.665833 |
| -6 | -102 | 18 | 0.677248 |
| -48 | -54 | 51 | 0.678299 |
| -24 | -99 | 18 | 0.678957 |
| 0 | -75 | 48 | 0.679791 |
| -3 | -81 | 45 | 0.681296 |
| -51 | -54 | -24 | 0.681359 |
| 6 | -9 | -33 | 0.682498 |
| 3 | -48 | -57 | 0.689097 |
| -33 | -84 | 33 | 0.698447 |
| -33 | -81 | 45 | 0.700025 |
| -3 | -9 | -33 | 0.701839 |
| 27 | 24 | 48 | 0.704903 |
| -6 | -96 | 18 | 0.706015 |
| 42 | -63 | 45 | 0.707208 |
| -6 | -99 | 18 | 0.708864 |
| -33 | -84 | 36 | 0.709912 |
| -9 | -81 | 54 | 0.71005 |
| -39 | -45 | 60 | 0.7136 |
| -24 | 39 | 42 | 0.715439 |
| -36 | -84 | 30 | 0.715563 |
| -42 | -78 | 30 | 0.72161 |
| 36 | 0 | -6 | 0.723387 |
| 0 | -78 | 51 | 0.73059 |
| 27 | 24 | 45 | 0.732984 |
| -24 | -99 | 15 | 0.737921 |
| -12 | -99 | 15 | 0.737956 |
| 0 | -3 | -6 | 0.744077 |
| -24 | 12 | -42 | 0.745528 |
| -39 | -54 | 54 | 0.745597 |
| -30 | -84 | 33 | 0.749612 |
| -42 | -75 | 30 | 0.752076 |
| -12 | -102 | 12 | 0.754813 |
| -36 | -75 | 45 | 0.755044 |
| -6 | -75 | 48 | 0.755362 |
| -42 | -51 | 63 | 0.76211 |
| -33 | -75 | 45 | 0.765006 |
| 0 | -45 | -57 | 0.769296 |
| 15 | -69 | 57 | 0.770805 |
| -6 | -9 | -36 | 0.771344 |
| 24 | 24 | 45 | 0.771567 |
| 15 | -63 | 72 | 0.772881 |
| 57 | -3 | 3 | 0.774343 |
| -42 | -51 | 54 | 0.777218 |
| 63 | -12 | 3 | 0.777539 |
| -48 | -54 | 48 | 0.779794 |
| 63 | -15 | 6 | 0.780669 |
| 9 | -12 | -36 | 0.782293 |
| -30 | 6 | -42 | 0.782765 |
| -3 | -72 | 48 | 0.787462 |
| -48 | -54 | -27 | 0.787867 |
| -9 | -78 | 48 | 0.788112 |
| -3 | -75 | 45 | 0.800278 |
| -6 | -81 | 45 | 0.802528 |
| -3 | -96 | 15 | 0.806144 |
| -33 | -78 | 48 | 0.806531 |
| -21 | -99 | 15 | 0.807238 |
| -9 | -99 | 9 | 0.810211 |
| -6 | -3 | -12 | 0.816694 |
| 63 | -12 | 6 | 0.822909 |
| -27 | -84 | 33 | 0.825964 |
| -36 | -78 | 45 | 0.82643 |
| -33 | -87 | 36 | 0.827907 |
| -30 | 9 | -48 | 0.829291 |
| -45 | -51 | 51 | 0.83386 |
| -48 | -45 | 24 | 0.837351 |
| 24 | 24 | 48 | 0.843324 |
| -36 | -81 | 36 | 0.843374 |
| -12 | -99 | 12 | 0.843993 |
| 63 | -60 | 27 | 0.848002 |
| 54 | 0 | 3 | 0.849133 |
| -6 | -105 | 9 | 0.849548 |
| -15 | -102 | 15 | 0.850706 |
| -36 | -84 | 36 | 0.860074 |
| -45 | -51 | 54 | 0.861252 |
| -6 | -102 | 9 | 0.869459 |
| 18 | -36 | -3 | 0.871327 |
| 27 | 21 | 48 | 0.872059 |
| -24 | -96 | 18 | 0.872942 |
| 33 | -12 | -51 | 0.874638 |
| 45 | -63 | 45 | 0.874998 |
| 48 | 12 | 45 | 0.875554 |
| -33 | -87 | 33 | 0.876337 |
| -6 | -78 | 45 | 0.878597 |
| 54 | -30 | 12 | 0.880974 |
| 24 | 45 | 48 | 0.883939 |
| 66 | -18 | -12 | 0.884637 |
| -51 | -75 | 30 | 0.88815 |
| -33 | -84 | 30 | 0.888899 |
| -9 | -81 | 48 | 0.891533 |
| -48 | -75 | 30 | 0.893283 |
| -9 | -96 | 18 | 0.896783 |
| -24 | -96 | 15 | 0.89779 |
| 36 | -3 | -3 | 0.898594 |
| 54 | -33 | 12 | 0.899096 |
| 60 | -18 | -12 | 0.899445 |
| -36 | -48 | 63 | 0.902395 |
| 27 | 21 | 51 | 0.903222 |
| -27 | -84 | 36 | 0.906552 |
| -21 | 18 | -27 | 0.906841 |
| 36 | 36 | -18 | 0.909342 |
| 36 | 33 | -18 | 0.911249 |
| 42 | -63 | 48 | 0.913823 |
| 33 | -39 | -12 | 0.914053 |
| -45 | -75 | 30 | 0.914687 |
| 60 | -60 | 27 | 0.91616 |
| -9 | -105 | 9 | 0.916804 |
| -39 | -81 | 36 | 0.917203 |
| 57 | 0 | 3 | 0.917503 |
| 72 | -18 | -9 | 0.918989 |
| -30 | -84 | 36 | 0.921872 |
| 36 | -93 | 21 | 0.923263 |
| -39 | -81 | 27 | 0.924927 |
| -21 | -102 | 15 | 0.928895 |
| -27 | 9 | -48 | 0.930067 |
| 66 | -18 | -9 | 0.935799 |
| -9 | -78 | 54 | 0.937929 |
| -18 | -102 | 15 | 0.940045 |
| 12 | -69 | 54 | 0.944447 |
| -27 | -102 | -6 | 0.946689 |
| -39 | -84 | 33 | 0.950922 |
| -21 | 18 | -24 | 0.951818 |
| 0 | -78 | 48 | 0.957354 |
| 63 | -63 | 27 | 0.958457 |
| 0 | -48 | -57 | 0.959762 |
| 21 | -30 | -15 | 0.962079 |
| 12 | -87 | -18 | 0.962585 |
| -27 | -99 | -9 | 0.962814 |
| -36 | -81 | 27 | 0.963472 |
| -3 | -6 | -6 | 0.964143 |
| -12 | -102 | 9 | 0.96432 |
| 24 | 27 | 42 | 0.966971 |
| -51 | -57 | -27 | 0.967837 |
| 51 | 12 | 45 | 0.967938 |
| -6 | -102 | 12 | 0.969409 |
| 69 | -18 | -9 | 0.970692 |
| 3 | -45 | -54 | 0.970876 |
| 24 | 45 | 45 | 0.971261 |
| -30 | -81 | 48 | 0.971386 |
| -12 | -99 | 9 | 0.972127 |
| 42 | -60 | 45 | 0.972686 |
| -3 | 9 | 60 | 0.973849 |
| -6 | -99 | 12 | 0.974315 |
| -24 | 42 | 42 | 0.974332 |
| -30 | -63 | 60 | 0.975015 |
| -30 | 6 | -39 | 0.980926 |
| 18 | -69 | 60 | 0.981757 |
| -27 | 18 | -39 | 0.982245 |
| -27 | 39 | 42 | 0.986441 |
| -27 | -99 | 18 | 0.988279 |
| -27 | 15 | -39 | 0.988289 |
| -12 | -102 | 18 | 0.993906 |
| -9 | -96 | 15 | 0.993936 |
| -51 | -54 | 45 | 0.994926 |
| -30 | -84 | 30 | 0.995086 |
| -39 | -45 | 63 | 0.995559 |
| -36 | -87 | 33 | 0.996156 |
| -6 | -105 | 12 | 0.998095 |
| -30 | -66 | 63 | 0.998574 |
| -33 | 9 | -42 | 0.999326 |
| -51 | -60 | 48 | 0.999326 |
|  |  |  |  |

Notes: RBVC, retinal blood vessel curve.

**Table S11. There was no significant correlation between cortical thickness of the left hemisphere and RBVC in HC and MCI.**

| Variable | Coefficient | P-value |
| --- | --- | --- |
| lh_caudalanteriorcingulate_thickness | -0.001 | 0.991 |
| lh_caudalmiddlefrontal_thickness | 0.025 | 0.772 |
| lh_cuneus_thickness | 0.013 | 0.924 |
| lh_entorhinal_thickness | 0.008 | 0.827 |
| lh_fusiform_thickness | 0.074 | 0.46 |
| lh_inferiorparietal_thickness | -0.031 | 0.748 |
| lh_inferiortemporal_thickness | -0.048 | 0.544 |
| lh_isthmuscingulate_thickness | -0.011 | 0.863 |
| lh_lateraloccipital_thickness | -0.02 | 0.886 |
| lh_lateralorbitofrontal_thickness | 0.065 | 0.484 |
| lh_lingual_thickness | 0.063 | 0.561 |
| lh_medialorbitofrontal_thickness | 0.017 | 0.866 |
| lh_middletemporal_thickness | 0.031 | 0.735 |
| lh_parahippocampal_thickness | 0.054 | 0.243 |
| lh_paracentral_thickness | -0.128 | 0.111 |
| lh_parsopercularis_thickness | 0.018 | 0.899 |
| lh_parsorbitalis_thickness | 0.102 | 0.303 |
| lh_parstriangularis_thickness | 0.087 | 0.439 |
| lh_pericalcarine_thickness | -0.163 | 0.07 |
| lh_postcentral_thickness | 0.106 | 0.238 |
| lh_posteriorcingulate_thickness | -0.044 | 0.604 |
| lh_precentral_thickness | -0.042 | 0.473 |
| lh_precuneus_thickness | 0.019 | 0.892 |
| lh_rostralanteriorcingulate_thickness | 0.024 | 0.797 |
| lh_rostralmiddlefrontal_thickness | 0.165 | 0.103 |
| lh_superiorfrontal_thickness | -0.006 | 0.96 |
| lh_superiorparietal_thickness | 0.083 | 0.471 |
| lh_superiortemporal_thickness | -0.04 | 0.726 |
| lh_supramarginal_thickness | -0.022 | 0.827 |
| lh_transversetemporal_thickness | 0.13 | 0.115 |
| lh_insula_thickness | 0.121 | 0.179 |

Notes: RBVC, retinal blood vessel curve. lh, left hemisphere.

**Table S12. There was no significant correlation between cortical thickness of the right hemisphere and RBVC in HC and MCI.**

| Variable | Coefficient | P-value |
| --- | --- | --- |
| rh_caudalanteriorcingulate_thickness | 0.027 | 0.72 |
| rh_caudalmiddlefrontal_thickness | 0.098 | 0.461 |
| rh_cuneus_thickness | 0.064 | 0.545 |
| rh_entorhinal_thickness | -0.012 | 0.741 |
| rh_fusiform_thickness | -0.047 | 0.681 |
| rh_inferiorparietal_thickness | 0.107 | 0.262 |
| rh_inferiortemporal_thickness | -0.112 | 0.185 |
| rh_isthmuscingulate_thickness | 0.056 | 0.447 |
| rh_lateraloccipital_thickness | -0.031 | 0.798 |
| rh_lateralorbitofrontal_thickness | 0.049 | 0.534 |
| rh_lingual_thickness | 0.052 | 0.632 |
| rh_medialorbitofrontal_thickness | -0.028 | 0.802 |
| rh_middletemporal_thickness | -0.093 | 0.364 |
| rh_parahippocampal_thickness | 0.005 | 0.947 |
| rh_paracentral_thickness | -0.004 | 0.97 |
| rh_parsopercularis_thickness | -0.053 | 0.683 |
| rh_parsorbitalis_thickness | 0.096 | 0.301 |
| rh_parstriangularis_thickness | 0.11 | 0.339 |
| rh_pericalcarine_thickness | -0.036 | 0.676 |
| rh_postcentral_thickness | 0.006 | 0.945 |
| rh_posteriorcingulate_thickness | -0.033 | 0.702 |
| rh_precentral_thickness | -0.087 | 0.136 |
| rh_precuneus_thickness | -0.137 | 0.254 |
| rh_rostralanteriorcingulate_thickness | -0.011 | 0.922 |
| rh_rostralmiddlefrontal_thickness | 0.216 | 0.08 |
| rh_superiorfrontal_thickness | 0.044 | 0.742 |
| rh_superiorparietal_thickness | 0.091 | 0.432 |
| rh_superiortemporal_thickness | -0.128 | 0.297 |
| rh_supramarginal_thickness | -0.072 | 0.371 |
| rh_transversetemporal_thickness | 0.009 | 0.911 |
| rh_insula_thickness | 0.115 | 0.19 |

Notes: RBVC, retinal blood vessel curve. rh, right hemisphere.

**Table S13. There was no significant correlation between cortical thickness of the left hemisphere and RBVC in HC and AD.**

| Variable | Coefficient | P-value |
| --- | --- | --- |
| lh_caudalanteriorcingulate_thickness | 0.013 | 0.85 |
| lh_caudalmiddlefrontal_thickness | 0.033 | 0.649 |
| lh_cuneus_thickness | -0.061 | 0.65 |
| lh_entorhinal_thickness | -0.003 | 0.908 |
| lh_fusiform_thickness | -0.016 | 0.859 |
| lh_inferiorparietal_thickness | 0.116 | 0.171 |
| lh_inferiortemporal_thickness | 0.014 | 0.85 |
| lh_isthmuscingulate_thickness | -0.066 | 0.285 |
| lh_lateraloccipital_thickness | 0.094 | 0.446 |
| lh_lateralorbitofrontal_thickness | 0.068 | 0.467 |
| lh_lingual_thickness | -0.045 | 0.679 |
| lh_medialorbitofrontal_thickness | 0.021 | 0.791 |
| lh_middletemporal_thickness | 0.029 | 0.721 |
| lh_parahippocampal_thickness | -0.026 | 0.46 |
| lh_paracentral_thickness | -0.125 | 0.108 |
| lh_parsopercularis_thickness | 0.026 | 0.842 |
| lh_parsorbitalis_thickness | 0.066 | 0.354 |
| lh_parstriangularis_thickness | 0.074 | 0.543 |
| lh_pericalcarine_thickness | -0.116 | 0.141 |
| lh_postcentral_thickness | 0.129 | 0.107 |
| lh_posteriorcingulate_thickness | -0.136 | 0.125 |
| lh_precentral_thickness | -0.049 | 0.455 |
| lh_precuneus_thickness | -0.089 | 0.373 |
| lh_rostralanteriorcingulate_thickness | 0.038 | 0.6 |
| lh_rostralmiddlefrontal_thickness | 0.018 | 0.849 |
| lh_superiorfrontal_thickness | -0.003 | 0.978 |
| lh_superiorparietal_thickness | 0.09 | 0.308 |
| lh_superiortemporal_thickness | 0.095 | 0.449 |
| lh_supramarginal_thickness | 0.085 | 0.348 |
| lh_transversetemporal_thickness | 0.019 | 0.79 |
| lh_insula_thickness | 0.133 | 0.057 |

Notes: RBVC, retinal blood vessel curve. lh, left hemisphere.

**Table S14. There was no significant correlation between cortical thickness of the right hemisphere and RBVC in HC and AD.**

| Variable | Coefficient | P-value |
| --- | --- | --- |
| rh_caudalanteriorcingulate_thickness | -0.017 | 0.753 |
| rh_caudalmiddlefrontal_thickness | -0.044 | 0.609 |
| rh_cuneus_thickness | 0.044 | 0.654 |
| rh_entorhinal_thickness | -0.027 | 0.298 |
| rh_fusiform_thickness | -0.047 | 0.472 |
| rh_inferiorparietal_thickness | -0.037 | 0.645 |
| rh_inferiortemporal_thickness | -0.042 | 0.484 |
| rh_isthmuscingulate_thickness | -0.113 | 0.133 |
| rh_lateraloccipital_thickness | -0.013 | 0.909 |
| rh_lateralorbitofrontal_thickness | -0.022 | 0.727 |
| rh_lingual_thickness | -0.052 | 0.538 |
| rh_medialorbitofrontal_thickness | 0.042 | 0.594 |
| rh_middletemporal_thickness | -0.056 | 0.422 |
| rh_parahippocampal_thickness | -0.04 | 0.335 |
| rh_paracentral_thickness | -0.035 | 0.736 |
| rh_parsopercularis_thickness | -0.053 | 0.569 |
| rh_parsorbitalis_thickness | 0.025 | 0.678 |
| rh_parstriangularis_thickness | 0.016 | 0.873 |
| rh_pericalcarine_thickness | 0 | 0.995 |
| rh_postcentral_thickness | -0.024 | 0.686 |
| rh_posteriorcingulate_thickness | -0.069 | 0.389 |
| rh_precentral_thickness | -0.036 | 0.617 |
| rh_precuneus_thickness | -0.074 | 0.36 |
| rh_rostralanteriorcingulate_thickness | 0.031 | 0.57 |
| rh_rostralmiddlefrontal_thickness | 0.03 | 0.72 |
| rh_superiorfrontal_thickness | 0.002 | 0.981 |
| rh_superiorparietal_thickness | 0.006 | 0.935 |
| rh_superiortemporal_thickness | -0.07 | 0.366 |
| rh_supramarginal_thickness | -0.101 | 0.239 |
| rh_transversetemporal_thickness | 0.008 | 0.898 |
| rh_insula_thickness | 0.058 | 0.439 |

Notes: RBVC, retinal blood vessel curve. rh, right hemisphere.

**Table S15. There was no significant correlation between cortical thickness of the left hemisphere and RNFL in HC and AD.**

| Variable | Coefficient | P-value |
| --- | --- | --- |
| lh_caudalanteriorcingulate_thickness | -0.241 | 0.783 |
| lh_caudalmiddlefrontal_thickness | -0.712 | 0.425 |
| lh_cuneus_thickness | 1.178 | 0.474 |
| lh_entorhinal_thickness | -0.342 | 0.3 |
| lh_fusiform_thickness | -0.1 | 0.929 |
| lh_inferiorparietal_thickness | -0.241 | 0.823 |
| lh_inferiortemporal_thickness | -0.706 | 0.453 |
| lh_isthmuscingulate_thickness | 0.691 | 0.369 |
| lh_lateraloccipital_thickness | 0.01 | 0.995 |
| lh_lateralorbitofrontal_thickness | 0.28 | 0.812 |
| lh_lingual_thickness | 0.116 | 0.931 |
| lh_medialorbitofrontal_thickness | -0.775 | 0.433 |
| lh_middletemporal_thickness | 0.917 | 0.359 |
| lh_parahippocampal_thickness | 0.103 | 0.812 |
| lh_paracentral_thickness | -0.331 | 0.74 |
| lh_parsopercularis_thickness | -0.933 | 0.566 |
| lh_parsorbitalis_thickness | -0.23 | 0.796 |
| lh_parstriangularis_thickness | -0.929 | 0.537 |
| lh_pericalcarine_thickness | -0.401 | 0.69 |
| lh_postcentral_thickness | 0.332 | 0.746 |
| lh_posteriorcingulate_thickness | 0.262 | 0.817 |
| lh_precentral_thickness | -0.865 | 0.282 |
| lh_precuneus_thickness | 1.033 | 0.401 |
| lh_rostralanteriorcingulate_thickness | 0.954 | 0.277 |
| lh_rostralmiddlefrontal_thickness | -0.056 | 0.961 |
| lh_superiorfrontal_thickness | 0.871 | 0.475 |
| lh_superiorparietal_thickness | 0.492 | 0.656 |
| lh_superiortemporal_thickness | -0.296 | 0.85 |
| lh_supramarginal_thickness | -1.894 | 0.081 |
| lh_transversetemporal_thickness | 2.042 | 0.017 |
| lh_insula_thickness | 0.363 | 0.691 |

Notes: RNFL, retinal nerve fiber layer. lh, left hemisphere.

**Table S16. There was no significant correlation between cortical thickness of the right hemisphere and RNFL in HC and AD.**

| Variable | Coefficient | P-value |
| --- | --- | --- |
| rh_caudalanteriorcingulate_thickness | 0.239 | 0.719 |
| rh_caudalmiddlefrontal_thickness | 0.782 | 0.462 |
| rh_cuneus_thickness | -0.506 | 0.677 |
| rh_entorhinal_thickness | -0.231 | 0.468 |
| rh_fusiform_thickness | 0.153 | 0.851 |
| rh_inferiorparietal_thickness | 0.482 | 0.629 |
| rh_inferiortemporal_thickness | 0.025 | 0.973 |
| rh_isthmuscingulate_thickness | 1.049 | 0.268 |
| rh_lateraloccipital_thickness | 0.298 | 0.83 |
| rh_lateralorbitofrontal_thickness | 0.264 | 0.739 |
| rh_lingual_thickness | 0.804 | 0.438 |
| rh_medialorbitofrontal_thickness | 0.931 | 0.34 |
| rh_middletemporal_thickness | 0.069 | 0.936 |
| rh_parahippocampal_thickness | 0.247 | 0.633 |
| rh_paracentral_thickness | 1.175 | 0.353 |
| rh_parsopercularis_thickness | 0.792 | 0.488 |
| rh_parsorbitalis_thickness | -0.16 | 0.833 |
| rh_parstriangularis_thickness | 0.446 | 0.721 |
| rh_pericalcarine_thickness | 0.035 | 0.971 |
| rh_postcentral_thickness | 0.47 | 0.526 |
| rh_posteriorcingulate_thickness | 0.402 | 0.686 |
| rh_precentral_thickness | -0.218 | 0.808 |
| rh_precuneus_thickness | 0.85 | 0.4 |
| rh_rostralanteriorcingulate_thickness | 0.843 | 0.208 |
| rh_rostralmiddlefrontal_thickness | 0.949 | 0.358 |
| rh_superiorfrontal_thickness | 1.271 | 0.218 |
| rh_superiorparietal_thickness | 0.344 | 0.691 |
| rh_superiortemporal_thickness | 0.854 | 0.371 |
| rh_supramarginal_thickness | 0.488 | 0.652 |
| rh_transversetemporal_thickness | 0.146 | 0.856 |
| rh_insula_thickness | 1.303 | 0.153 |

Notes: RNFL, retinal nerve fiber layer. rh, right hemisphere.

**Table S17. There was no significant correlation between cortical thickness of the left hemisphere and GCL-IPL in HC and AD.**

| Variable | Coefficient | P-value |
| --- | --- | --- |
| lh_caudalanteriorcingulate_thickness | -0.367 | 0.945 |
| lh_caudalmiddlefrontal_thickness | -4.09 | 0.455 |
| lh_cuneus_thickness | 0.758 | 0.94 |
| lh_entorhinal_thickness | -2.443 | 0.223 |
| lh_fusiform_thickness | -2.134 | 0.756 |
| lh_inferiorparietal_thickness | -1.805 | 0.785 |
| lh_inferiortemporal_thickness | -1.119 | 0.847 |
| lh_isthmuscingulate_thickness | 1.824 | 0.702 |
| lh_lateraloccipital_thickness | -2.352 | 0.803 |
| lh_lateralorbitofrontal_thickness | 1.537 | 0.831 |
| lh_lingual_thickness | 3.727 | 0.651 |
| lh_medialorbitofrontal_thickness | -7.786 | 0.191 |
| lh_middletemporal_thickness | 6.189 | 0.311 |
| lh_parahippocampal_thickness | 0.986 | 0.71 |
| lh_paracentral_thickness | -2.909 | 0.634 |
| lh_parsopercularis_thickness | 8.05 | 0.416 |
| lh_parsorbitalis_thickness | 0.402 | 0.941 |
| lh_parstriangularis_thickness | 2.274 | 0.806 |
| lh_pericalcarine_thickness | -9.018 | 0.131 |
| lh_postcentral_thickness | 0.625 | 0.921 |
| lh_posteriorcingulate_thickness | -4.092 | 0.554 |
| lh_precentral_thickness | -4.834 | 0.328 |
| lh_precuneus_thickness | 0.917 | 0.904 |
| lh_rostralanteriorcingulate_thickness | -1.234 | 0.821 |
| lh_rostralmiddlefrontal_thickness | 3.817 | 0.582 |
| lh_superiorfrontal_thickness | 5.181 | 0.488 |
| lh_superiorparietal_thickness | -1.562 | 0.818 |
| lh_superiortemporal_thickness | 1.591 | 0.868 |
| lh_supramarginal_thickness | -11.336 | 0.089 |
| lh_transversetemporal_thickness | 12.341 | 0.019 |
| lh_insula_thickness | 8.873 | 0.099 |

Notes: GCL, ganglion cell layer; IPL, inner plexiform layer. lh, left hemisphere.

**Table S18. There was no significant correlation between cortical thickness of the right hemisphere and GCL-IPL in HC and AD.**

| Variable | Coefficient | P-value |
| --- | --- | --- |
| rh_caudalanteriorcingulate_thickness | -6.375 | 0.104 |
| rh_caudalmiddlefrontal_thickness | 3.44 | 0.599 |
| rh_cuneus_thickness | -12.86 | 0.07 |
| rh_entorhinal_thickness | -2.296 | 0.232 |
| rh_fusiform_thickness | -4.332 | 0.38 |
| rh_inferiorparietal_thickness | 0.119 | 0.985 |
| rh_inferiortemporal_thickness | 0.628 | 0.889 |
| rh_isthmuscingulate_thickness | 4.314 | 0.462 |
| rh_lateraloccipital_thickness | -7.035 | 0.403 |
| rh_lateralorbitofrontal_thickness | 1.637 | 0.736 |
| rh_lingual_thickness | 0.722 | 0.91 |
| rh_medialorbitofrontal_thickness | 3.491 | 0.562 |
| rh_middletemporal_thickness | 1.691 | 0.75 |
| rh_parahippocampal_thickness | -2.685 | 0.393 |
| rh_paracentral_thickness | -0.227 | 0.977 |
| rh_parsopercularis_thickness | 3.054 | 0.664 |
| rh_parsorbitalis_thickness | 1.631 | 0.725 |
| rh_parstriangularis_thickness | 7.446 | 0.324 |
| rh_pericalcarine_thickness | -9.887 | 0.081 |
| rh_postcentral_thickness | 4.085 | 0.366 |
| rh_posteriorcingulate_thickness | -5.579 | 0.354 |
| rh_precentral_thickness | -6.98 | 0.194 |
| rh_precuneus_thickness | 3.466 | 0.577 |
| rh_rostralanteriorcingulate_thickness | 0.8 | 0.849 |
| rh_rostralmiddlefrontal_thickness | 5.987 | 0.344 |
| rh_superiorfrontal_thickness | 4.474 | 0.486 |
| rh_superiorparietal_thickness | -1.149 | 0.829 |
| rh_superiortemporal_thickness | -1.457 | 0.805 |
| rh_supramarginal_thickness | -6.318 | 0.336 |
| rh_transversetemporal_thickness | 0.639 | 0.897 |
| rh_insula_thickness | 9.715 | 0.077 |

Notes: GCL, ganglion cell layer; IPL, inner plexiform layer. rh, right hemisphere.
